# Identification of intestinal enteroendocrine cell subtypes and their associated hormones in zebrafish

**DOI:** 10.1371/journal.pbio.3003522

**Published:** 2025-12-18

**Authors:** Margaret Morash, Richard G. Kay, Erik J. Soderblom, Grace H. MacLean, Jia Wen, Peyton J. Moore, Colin R. Lickwar, Mujahid Ali Shah, Julia Ganz, Fiona M. Gribble, Frank Reimann, Rodger A. Liddle, John F. Rawls

**Affiliations:** 1 Department of Molecular Genetics and Microbiology, Duke University School of Medicine, Durham, North Carolina, United States of America; 2 Institute of Metabolic Science, Addenbrooke’s Hospital, University of Cambridge, Cambridge, United Kingdom; 3 Proteomics and Metabolomics Shared Resource, Duke University School of Medicine, Durham, North Carolina, United States of America; 4 Department of Integrative Biology, Michigan State University, East Lansing, Michigan, United States of America; 5 Division of Gastroenterology, Department of Medicine, Duke University School of Medicine, Durham, North Carolina, United States of America; University of Pennsylvania School of Medicine, UNITED STATES OF AMERICA

## Abstract

Enteroendocrine cells (EECs) are rare sensory cells in the intestinal epithelium that coordinate digestive physiology by secreting a diverse repertoire of peptide hormones. These hormones are the main effectors of EEC function, and their characterization requires direct observation by mass spectrometry due to the specialized protein cleavage and posttranslational modifications that yield their mature forms. Based on the distinct subset of hormones they predominantly secrete, EECs can be categorized into subtypes. How each EEC subtype is specified, however, remains poorly understood. Here, we describe EEC subtype differentiation and hormone production in the zebrafish. Using single-cell RNA sequencing data, we identified EEC progenitors and six EEC subtypes in zebrafish and revealed that their expression profiles are consistent across larval and adult stages. Mass spectrometry analysis of isolated zebrafish EECs identified highly processed peptides derived from 19 of 23 hormone-coding genes expressed by EECs, including a previously undescribed zebrafish *secretin* ortholog. We assembled reporters for zebrafish EEC subtypes to test the lineage relationships between EEC subtypes and the EEC progenitor population, which expresses *neurogenin 3 (neurog3)*. Despite its essential role in mammalian EEC differentiation, we found that selective cytotoxic ablation of *neurog3*+ cells in zebrafish only reduced a subset of EEC subtypes and loss of the *neurog3* gene had no impact on EEC numbers. Finally, we discovered that selective ablation of *ghrelin*+ EECs reduced a different subset of EEC subtypes, together suggesting that *neurog3*+ and *ghrelin*+ cells serve as distinct precursors for separate EEC subtypes. We anticipate these observations and resources will facilitate future studies in the zebrafish to discern the developmental biology, physiology, and endocrinology of EEC subtypes.

## Abstract 2

Enteroendocrine cells (EECs) in the gut epithelium secrete diverse hormones that regulate digestion, energy use, and behavior. This study identifies EEC subtypes and their hormone profiles in zebrafish, exploring their lineage and determining that neurogenin3+ and ghrelin+ cells are distinct progenitors for different subtypes.

## Introduction

Enteroendocrine cells (EECs) are specialized intestinal epithelial cells that help coordinate digestive physiology in response to ingested food, resident microorganisms, and other stimuli. EECs are an ancient and conserved feature of the digestive tract in bilaterian animals [1–3], with evidence of cells with similar function in distantly related placozoa [4,5]. EECs respond to stimuli by releasing secretory vesicles containing at least one of over a dozen different peptide hormones they produce. These EEC peptide hormones govern diverse physiologic functions like nutrient digestion, gut motility, satiety, and insulin signaling [6,7]. Each EEC peptide hormone is produced through the transcription and translation of its preprohormone protein, which is then subjected to successive cleavage and posttranslational modification steps prior to secretion from the cell [8]. These highly specialized processing steps make it largely impossible to predict the final functional peptide’s form from the primary amino acid sequence alone, necessitating direct observation of peptides through sensitive peptidomic studies to shed light on their biology.

As the functions of different EEC peptide hormones are highly varied and sometimes antagonistic, ensuring the release of the appropriate hormone is essential for mounting the appropriate physiologic response to a given stimulus. This is largely accomplished through the differentiation of EECs into distinct subtypes that each secrete a single or small subset of hormones [6,7]. EEC differentiation begins with secretory progenitor cells within the intestinal epithelium, which can give rise to any secretory lineage, such as enteroendocrine, goblet, tuft, and Paneth cells [9]. In mouse and humans, transient expression of the transcription factor *Neurogenin 3* (*Neurog3*) commits these secretory progenitors to an EEC fate, and *Neurog3* expression is therefore used as a marker of EEC progenitors [10–14]. Select transcription factors including *Neuronal differentiation 1* (*Neurod1)* are subsequently broadly expressed to aid in the differentiation of *Neurog3*+ EEC progenitors into mature EEC subtypes [15–18]. More recent studies in mammalian organoid models have proposed successive factors expressed only in a subset of EECs that drive EECs towards a single specific subtype fate [19,20]. This work has begun to shed light on how EEC subtype identity—and thus the cell’s hormone profile and function—is determined, but further studies and in vivo testing are needed to elucidate this important process.

While EEC subtypes were historically believed to be terminally differentiated and therefore insulated from one another once committed, recent work in organoids indicated that some EEC subtypes can transition into other subtypes as they travel up the crypt-villus axis [21]. This new concept of EEC subtype plasticity raises interesting questions about the lineage relationships between EEC subtypes. While a new concept in the intestine, there are examples of plastic lineage relationships between endocrine subtypes in the stomach and pancreatic islet where ghrelin-producing cells have been shown to give rise to other endocrine populations [22]. Multiple single-cell RNA sequencing (scRNA-seq) studies in mouse have hinted at a similar role for *Ghrelin* (*Ghrl*) expressing cells in the small intestine as they identified a *Ghrl*-expressing EEC progenitor population [23–25]. RNA velocity and partition-based graph abstraction of one of those datasets predicted a differentiation trajectory through the *Ghrl*+ progenitor cluster to many EEC subtypes [24], but a role for *Ghrl*+ cells in EEC differentiation has never been directly tested. Together, these studies suggest that establishment of EEC subtype identity may be more dynamic than previously thought.

The zebrafish is an ideal vertebrate model to study EEC subtype dynamics. We previously revealed extensive similarities between EEC morphology, sensitivity, and neural connectivity in zebrafish and mammals [26,27]. Additionally, many important aspects of EEC differentiation are conserved between zebrafish, mammals, and insects [28–33], although there are slight differences. Specifically, *neurog3*, the zebrafish ortholog of *Neurog3*, is expressed in some zebrafish EECs, but is thought to play a smaller role in EEC differentiation, although its exact function remains unclear [28,30]. Instead, *neurod1*, the zebrafish ortholog of *Neurod1*, has been shown to be necessary and sufficient for EEC differentiation [26,27,31]. Similar to mammals, *neurod1* is expressed across all EECs in zebrafish and is used as a pan-EEC reporter [26]. Critically, the optical transparency of zebrafish enables in vivo imaging of gut anatomy and physiology through early adulthood. Combined with the ease of generating genetic reporters, this provides a powerful system for investigating EEC subtype development and dynamics in their native context. However, zebrafish EEC subtypes and their processed hormone products have not been previously described.

In this study, we aimed to define EEC subtypes in zebrafish and establish an experimental system in which to study their development and function. To do this, we leveraged scRNA-seq data from larval and adult zebrafish to reveal seven distinct EEC populations, six of which we characterized in vivo across larval development. We also deployed peptidomic analysis to demonstrate that these EECs produce a wide array of highly processed and conserved peptide hormones. To better understand how these EEC subtypes develop, we probed their lineage relationships by genetically restricting certain cell states and assessing the impacts on the remaining subtype populations. In this way, we were able to reveal that both predicted contributors, such as *neurog3*+ cells, as well as untested factors, such as *ghrl*+ cells, play major roles in development of EEC subtypes in zebrafish.

## Results

### Zebrafish EEC transcriptional signatures identify EEC progenitors and six distinct EEC subtypes

**Fig 1:**
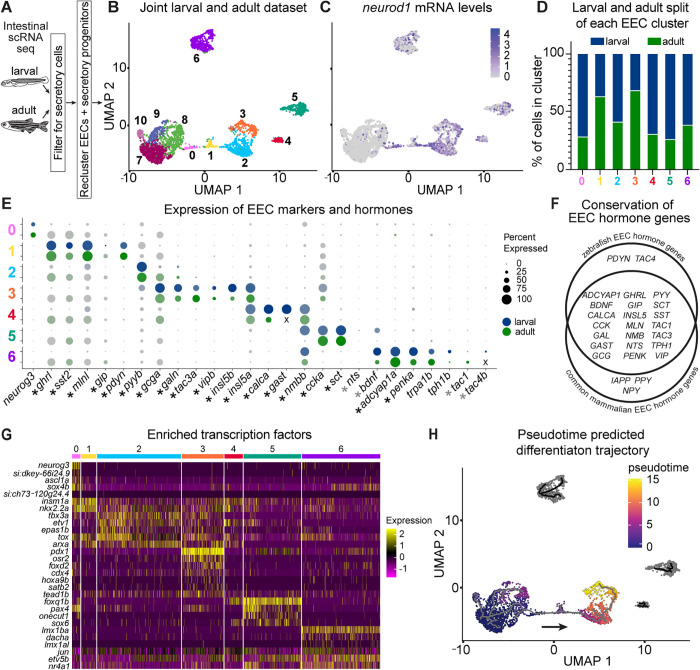
scRNA-seq of larval and adult EECs identifies 7 distinct clusters. **(A)** Schematic overview of identifying secretory cells from published larval [48] and adult [49] datasets. **(B)** UMAP of secretory progenitors and EECs from larval and adult zebrafish identifies 11 clusters. **(C)** Normalized expression of the zebrafish pan-EEC transcription factor and reporter *neurod1* labels clusters 0–6. **(D)** The percentage of cells derived from either the larval (blue) or adult (green) datasets in each of the 7 EEC clusters. **(E)** Scaled expression dotplot of relevant marker genes of the 7 EEC clusters. Dots represent gene expression in the specified cluster. Dot radius corresponds to the percent of cells expressing the gene and dot transparency corresponds to expression intensity. Expression is reported for both the larval (blue) and adult (green) cells in the cluster, and genes not annotated in a given dataset are noted with an X. EEC hormone coding genes are marked with an asterisk next to their gene name on the *x* axis. Black asterisks indicate genes we identified peptides from and gray asterisks mark those with no detected peptides (Figs 2B and S4). Note, annotations of *gast* (see scRNA-seq methods), *tac4b* (see scRNA-seq methods, S15 Fig), and *sct* (see Results and Figs 2 and S6) were not previously identified and were defined in this paper. **(F)** Venn diagram of EEC hormone coding gene homologs found in zebrafish and mammals. **(G)** Heatmap of expression of transcription factors significantly enriched in each EEC cluster in both larval and adult cells. **(H)** UMAP colored by pseudotime as determined by Monocle 3 when cluster 7 was provided as the origin. Brighter coloring indicates “older” cell age, and the gray line is the predicted differentiation trajectory. Underlying data can be found in S1 Data.

To understand EEC subtype diversity and differentiation in zebrafish, we used established markers of secretory cells [9,28,29,34–47] to identify and subset these populations from published intestinal scRNA-seq datasets derived from larval [48] and adult [49] zebrafish (S1A and S1B Fig). Integration of these secretory cells from larval and adult datasets showed the expected structure of a hub of secretory progenitor cells with spokes of distinct secretory populations (S1C and S1D Fig). With this confirmation, we then subsetted and reintegrated just EECs and secretory progenitors into a new combined larval and adult dataset (Fig 1A and 1B). Within this dataset, we identified clusters 0–6 as EECs based on *neurod1* expression (Fig 1C) and clusters 7–10 as secretory progenitors (S2A Fig). We found that each EEC cluster contained both larval and adult cells with no apparent age dependency for any EEC subtype (Figs 1D, S2B, and S2C), suggesting that EEC subtypes and their underlying gene expression programs are surprisingly consistent between larval and mature adult stages in zebrafish.

To identify the shared features of larval and adult EECs, we assessed the expression of EEC markers and hormone coding genes in the combined dataset (Fig 1E). Hormone coding genes (marked with an asterisk in Fig 1E) are often used to separate EECs into subtypes. In accord, we found that expression of each EEC hormone coding gene is largely partitioned into a single cluster with remarkable consistency across larval and adult stages. All *neurod1*+ clusters expressed EEC hormone coding genes except for cluster 0, which instead expressed *neurog3*. This pattern is consistent with features of EEC progenitors in mammals, which are marked by *Neurog3* expression and have not yet begun to express hormone coding genes [10–14,50]. As such, we operationally defined cluster 0 as a putative EEC progenitor population and clusters 1–6 as mature EEC subtypes. We observed substantial overlap between the EEC hormone coding genes expressed in our zebrafish dataset and those reported in published mammalian datasets (Fig 1F) [19,23,24,51–55], highlighting the critical role of these signaling molecules during vertebrate evolution.

To test if the EEC subtype populations we identified are consistent across studies, we examined the EECs in another published larval zebrafish scRNA-seq dataset (S3A Fig) [56]. After selecting and re-clustering the EECs in that additional dataset, we found that the EEC hormone genes we highlighted in Fig 1E partitioned into clusters remarkably similarly to what we observed in our dataset (S3B Fig). The one exception was that *ghrl* and *pyyb* were expressed in two separate clusters in our combined dataset, but one in the additional dataset. However, the expression of *ghrl* and *pyyb* was non-overlapping in the additional dataset with *ghrl* primarily expressed at the boundary between the *pyyb*+ cluster and a cluster enriched for secretory progenitor markers (S3C Fig). This indicates that the zebrafish EEC subtypes described here are relatively consistent across independent studies.

To explore the broader transcriptional programs driving EEC subtype identity, we identified enriched genes specific to each cluster (S1 Table, S2D Fig). Using existing ontologies [57], we identified transcription factors that were significantly enriched in both larval and adult cells in each cluster (Fig 1G). We also performed pseudotime analysis [58], a computational method for ordering cells along a hypothetical timeline based on transcriptomic similarity that enables the study of cell differentiation dynamics. Using the secretory progenitor cluster 7 as the start point, this analysis predicted a differentiation trajectory from secretory progenitor clusters to EEC subtype clusters (Fig 1H). These complementary approaches highlighted certain patterns in EEC populations. For example, we note that putative EEC progenitor cluster 0 is enriched in *ascl1a* and *sox4b* in addition to *neurog3*. While mammalian *Neurog3* is the most canonical EEC progenitor marker, studies have also shown that *Ascl1/ascl1a* and *Sox4/sox4b* homologs label endocrine progenitor populations in mammals [19,24,41,59–61] and zebrafish [28,31,62,63]. EEC subtype cluster 1 is enriched for *insm1a* and *nkx2.2a* whose mammalian homologs *Insm1* and *Nkx2.2* are known to play a role in the differentiation of *Neurog3*+ EEC progenitors into mature EEC subtypes [64–67]. Together with the pseudotime data, these findings support a classification of cluster 0 as EEC progenitors and subtype cluster 1 as potentially an early EEC state. Interestingly, subtype cluster 1 also expresses the EEC hormone coding genes *ghrelin* (*ghrl*), *somatostatin* (*sst2*), *motilin-like* (*mlnl*), and *gastric inhibitory peptide* (*gip*), which are canonically thought to mark a mature EEC population [50,68]. EEC subtype clusters 2 and 3 appear to have a close relationship given their adjacent positions in pseudotime and the high expression of subtype cluster 2 enriched transcription factors in subtype cluster 3. EEC subtype cluster 3 is uniquely enriched for the known endocrine transcription factor *pdx1* [69–71] as well as *osr2*, which has been shown in mammals and zebrafish to be more highly expressed in the mid and distal small intestine [23,72], indicating a potential regional distribution of this subtype. Finally, subtype cluster 6, which expresses the rate-limiting enzyme in serotonin synthesis *tph1b* (Fig 1E), is enriched for transcription factors *lmx1ba* and *lmx1al* whose orthologs in mammals have been shown to be important for the differentiation of serotonergic neurons and EECs, respectively [66,73,74]. Serotonergic EECs, termed enterochromaffin cells, separate from other EECs in mammalian scRNA-seq studies [19,24,25,75]. Likewise, in our dataset, we find that subtype cluster 6 most clearly separates from the remaining EEC populations (Fig 1B). Altogether, this identifies cluster 6 as zebrafish enterochromaffin cells and suggests the divergence between enterochromaffin cells and other EECs is an ancestral trait to fishes and mammals. This *in silico* analysis of scRNA-seq data highlights a limited set of transcription factors that might play a role in promoting differentiation from secretory progenitors into discrete EEC subtypes.

### Zebrafish EECs produce fully processed and modified peptide hormones in vivo

**Fig 2:**
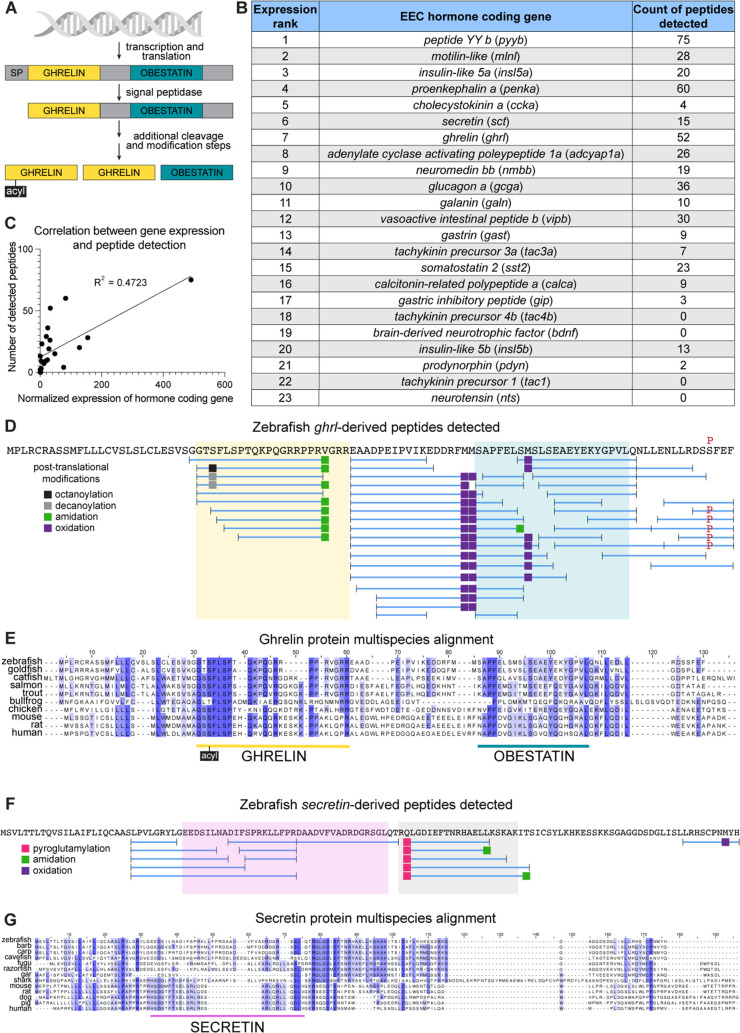
Peptidomic analysis of zebrafish EECs reveals production of conserved and novel peptides. **(A)** Schematic of peptide hormone processing using the human EEC hormone coding gene *GHRELIN* as an example. **(B)** Number of peptides identified from each zebrafish hormone coding gene ranked by normalized mRNA expression level as assessed by scRNA-seq (S1 Table). Note, annotations of *gast* (see scRNA-seq methods), *tac4b* (see scRNA-seq methods, S15 Fig), and *sct* (see Results and Figs 2 and S6) were not previously identified and were defined in this paper. **(C)** Correlation between the normalized mRNA expression of zebrafish EEC hormone coding genes and the number of peptides detected. **(D)** Representation of *ghrl-*derived peptides detected in zebrafish EECs. The primary amino acid sequence is shown at the top in black with observed missense variants indicated in red above. Blue horizontal lines below the amino acid sequence represent the individual peptides detected in our study with small black vertical lines denoting the stop and start of each peptide. Abutting peptides share a single vertical line to represent the stop of the preceding peptide and the start of the subsequent one. Different colored squares represent various posttranslational modifications detected. Yellow shading marks the region aligning to the functional ghrelin peptide in humans and teal shading marks the region aligning to the functional obestatin peptide in humans. **(E)** Alignment of the primary amino acid sequence of the Ghrelin protein in zebrafish (Uniprot F1QKX9), goldfish (Uniprot Q8AUU1), catfish (Ensembl ENSIPUT00015057246.1), salmon (Uniprot B3IYK1), trout (Uniprot A0A060WHA4), bullfrog (Uniprot Q90W22), chicken (Uniprot Q8AV73), mouse (Uniprot Q9EQX0), rat (Uniprot Q9QYH7), human (Uniprot Q9UBU3). Amino acids are color-coded based on their percent identity match across all the reported species with darker coloring indicating a more conserved residue. Human functional peptide annotations are labeled below, including the conserved acylation at the serine 3 residue. **(F)** Representation of peptides detected from the zebrafish *sct* gene. Annotation is as described in D, with pink shading marking the region aligning to the functional secretin peptide in humans and gray shading marking the region highly conserved across fishes. **(G)** Alignment of the primary amino acid sequence of the Secretin protein in zebrafish (Ensembl ENSDARG00000099233), Sumatra barb (NCBI LOC122330700), grass carp (NCBI LOC127507777), Pachon cavefish (Ensembl ENSAMXG00005000672), fugu (Ensembl enstrug00000026212), razorfish (NCBI CAJ1056004), gar (NCBI LOC107075768), elephant shark (NCBI LOC121850333), mouse (Uniprot Q08535), rat (Uniprot P11384), dog (Uniprot A0A813PAI0), pig (Uniprot P63298), human (Uniprot P09683). Coloring and annotation as described in **E**. Underlying data can be found in S1 Data.

EEC peptide hormones are derived from the initial translated preprohormone through several steps of specialized cleavage and posttranslational modification (Fig 2A). As a point of nomenclature, in this text we refer to the peptide-coding gene and transcript in italics (*GHRELIN* for human, *Ghrl* for mouse, and *ghrl* for zebrafish and other model organisms), the pro-peptide protein with a leading capitalization (Ghrelin), and the secreted peptide in lowercase (ghrelin). To understand what processed peptides are produced by zebrafish EECs, we performed mass spectrometry on sorted EECs from larval and adult samples. Due to technical limitations, adult samples contained roughly twice as many cells as larval samples, and peptides derived from adult samples are thus overrepresented in our peptidomic dataset (see Methods). In S2 Table, we report each peptide and the quantitation (area under the peak value) from each of the 3 larval and 3 adult biological replicate samples. However, we did not perform comparisons between values from larval versus adult samples as the lower cell content in larval samples meant that low or absent signal in larval samples could be due to the limit of detection of the instrument and not reflective of the biology of the system. Additionally, the EEC peptide data presented in Figs 2 and S4 only reports the detection of unique individual peptides and should not be interpreted as reflective of their natural relative abundance in EEC secretory granules.

Of the 23 hormone-coding genes transcriptionally expressed by zebrafish EECs (Fig 1E), we identified peptide fragments aligning to 19 of them (Fig 2B). Genes with no detected peptides included *neurotensin* (*nts*), *tachykinin precursor 1* (*tac1*), and *tachykinin 4b* (*tac4b*), which were very lowly expressed at the transcript level, and *brain-derived neurotrophic factor* (*bdnf*), whose peptide product is too large for optimal detection with our methodology. Of note, while expression of *tph1b* is used as a proxy for serotonin because it catalyzes the rate-limiting step in its formation, serotonin itself is derived from a single amino acid and is not suited to detection with our methodology. The EEC peptides we identified include products of hormone coding genes specifically expressed by EEC subtypes 1–6, suggesting each of those subtypes produce peptide hormones. We found a modest correlation between the expression level of a gene in scRNA-seq data (S1 Table) and the number of unique peptides detected that align to that gene (Fig 2C). We also identified peptides from other common components of the hormone-containing secretory vesicles released by EECs. Namely, we detected peptides from *proprotein convertase subtilisin/kexin type 1* and *2* (*pcsk1* and *pcsk2*), known for their role in specialized cleavage of peptide hormones [76], and *secretogranin 2a*, *2b*, *3*, and *5*, which help form the secretory vesicles and can be processed into hormones themselves (S4Q–S4X Fig) [77–80]. Together, these observations indicate that our EEC peptidomic approach effectively isolated and defined peptide contents of zebrafish EEC secretory vesicles.

Using this dataset, we generated an atlas of zebrafish EEC peptides including their sequence, posttranslational modifications, and alignment to homologous proteins in humans and other vertebrates. This is provided as a resource in the supplemental materials supporting this article (S4 Fig), and we highlight Ghrelin-derived peptides as an example (Fig 2D). In mammals, two main peptide hormone products from the *GHRELIN* gene have been described, ghrelin and obestatin [81,82]. Mammalian ghrelin is a 28-amino acid peptide that is often fatty acylated at the serine 3 residue [83]. While both acylated and deacylated ghrelin peptides are produced, fatty acylation improves binding to ghrelin’s receptor and thus is critical to its function [81,83]. As such, the serine 3 residue is highly conserved across species with evidence of acylation at this site in each vertebrate examined (Fig 2E) [81,84,85]. Indeed, studies in goldfish, a close relative of zebrafish, identified a functional 17-amino acid ghrelin peptide that is acylated at the serine 3 residue [84,86], and intraperitoneal injection of acylated (but not desacylated) ghrelin stimulated increased feeding in goldfish and grass carp [86,87]. Mammalian obestatin is a 22-amino acid peptide that was identified more recently by bioinformatic prediction [82], and its function is still debated [82,88]. We detected 52 Ghrelin-derived peptides in zebrafish, including 10 that align with the portion of the protein shown to encode a functional ghrelin peptide in humans and goldfish (Fig 2D and 2E) [86,89,90]. As fatty acylation is key to ghrelin’s function and not included in the posttranslational modification parameters of our initial search, we performed a second search for just ghrelin peptides and included octanoyl and decanoyl modifications in our parameters. Peptides with both octanoyl- and decanoyl-modified serine 3 residues were detected, demonstrating remarkable conservation of the processing and posttranslational modification of this peptide hormone (Figs 2D, S5A, and S5B). While functional studies remain to be performed, the alignment and modification data suggest these peptides could be endogenous zebrafish ghrelin. In contrast, while we detected peptides aligning to portions of the reported obestatin region, we did not detect any peptide spanning that entire region. Our data suggests obestatin may not be produced in fishes, although co-injection of a synthetic predicted obestatin-like peptide has been shown to inhibit the appetite-stimulating effects of injected ghrelin in grass carp [87].

While our data on Ghrelin-derived peptides confirmed our predictions based on studies in closely related organisms, we were also able to leverage our peptidomic dataset for novel discoveries. For example, we detected several peptides aligned to the largely unannotated gene *si:zfos-2372e4.1* (Fig 2F). Like most of the known peptide-coding genes, *si:zfos-2372e4.1* is highly expressed in a specific EEC cluster, namely cluster 5, which is also enriched for *ccka* expression (Fig 1E). Notably, in other zebrafish scRNA-seq datasets, expression of *ccka* is most highly correlated with expression of *si:zfos-2372e4.1* (*r* = 0.74) [91], demonstrating a consistency of this pattern across laboratories. However, *si:zfos-2372e4.1* had no gene or protein annotations. On our analysis, predictive software Signal P [92,93] assigned a high likelihood (0.81) that Si:zfos-2372e4.1 contains a signal peptide sequence at amino acid 1–23, indicating the protein is secreted. The prediction of a signal peptide is supported by the lack of detected peptides aligning to the N-terminus of the prepropeptide (Fig 2F).

To identify the potential functions of this gene, we performed a TBLASTN search using the full prepropeptide amino acid sequence, and the only similar sequences were in other fish species—largely in unannotated or predicted genes. Intriguingly, however, there was an approximately 20 amino acid sequence in Si:zfos-2372e4.1 that consistently aligned with those proteins in other species, suggesting that portion of the protein may be conserved and therefore perhaps functional. We looked specifically for any homology in gar, a holostean fish whose lineage diverged from teleost before the teleost-specific third round (3R) whole genome duplication (S6A Fig) [94]. Our search identified a gene in gar that, while also unannotated, allowed us to bridge to additional vertebrate species [95], including an elephant shark gene labeled as prosecretin with homology to *SECRETIN* (*SCT*) genes in humans and other tetrapods (S6B Fig) [96].

To further investigate the possibility that zebrafish *si:zfos-2372e4.1* may be a *SCT* ortholog, we performed a syntenic analysis (S6B Fig). We identified genes in the ~250,000 base pair region upstream and downstream of the human *SCT* gene. Over half of those genes we identified had orthologs in zebrafish as annotated by Ensembl [97], and we traced the chromosomal arrangement of those genes in the Chondrichthyes elephant shark (*Callorhinchhus milii*), the Osteichthyes gar (*Lepisosteus oculatus*), and the Osteichthyes zebrafish (*Danio rerio*). While some rearrangements did occur, the immediate neighborhood remained remarkably consistent across all species (S6B Fig, S3 Table). This syntenic conservation across bony fish, cartilaginous fish, and tetrapods supports our identification of *si:zfos-2372e4.1* as *sct* and suggests that this organization was present in the last common ancestor of all jawed vertebrates (S6A Fig) [98]. Given the evidence of homology from our TBLASTN and synteny assessments, we conclude that *si:zfos-2372e4.1* is the zebrafish *sct* ortholog.

We aligned both known Secretin prepropeptides and unannotated proteins that were returned from our TBLASTN search (Fig 2G). We found the homology within fishes and between fishes and mammals in the region annotated as human secretin was relatively limited. However, we did detect several peptides aligning to this region in zebrafish (shaded in pink in Fig 2F). Moreover, we detected several additional peptides that aligned to a region that showed strong homology in fishes (shaded in gray in Fig 2F). All of these peptides had an N-terminal pyroglutamylated glutamine residue, supporting that these are genuine processed peptides, and not just degradation products. Functional studies are needed to test which, if any, of the zebrafish Secretin-derived peptides have secretin-like or potentially novel function. While we highlight Ghrelin- and Secretin-derived peptides as examples of conservation and potential novelty, we anticipate that the many additional zebrafish EEC peptides included in S4 Fig will serve as a valuable resource for future studies.

### Zebrafish EEC subtypes show distinct expression patterns in vivo

**Fig 3:**
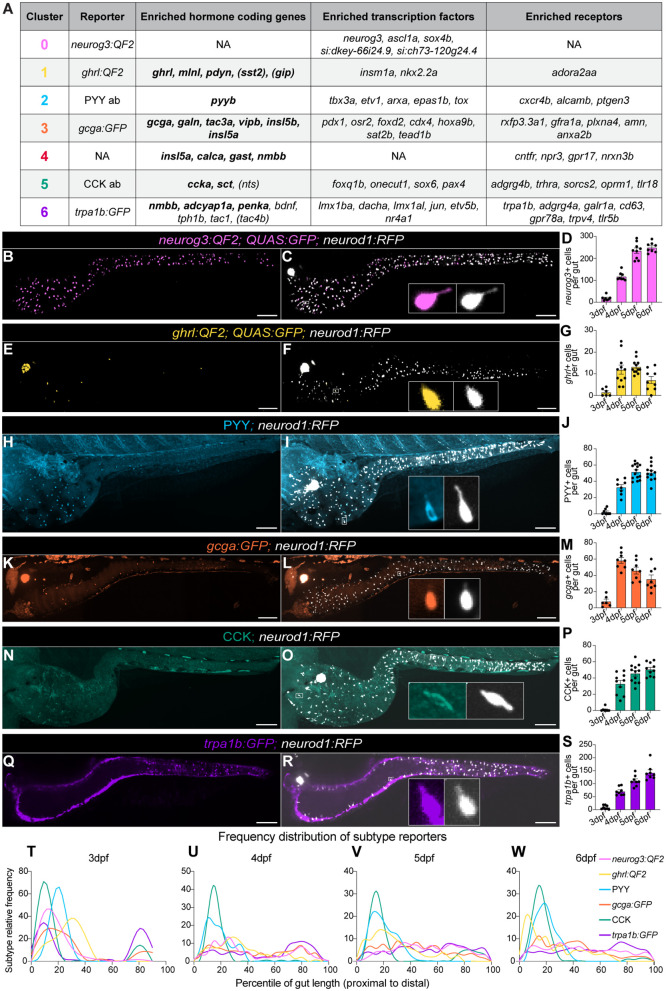
In vivo imaging of EEC subtypes shows distinct spatial and temporal patterns during larval development. **(A)** Summary of the hormones, transcription factors, and receptors significantly enriched in each EEC cluster and the reporter used to characterize that cluster. Bolding indicates genes from which we detected peptides. Parentheses indicate hormone-coding genes of interest expressed in that cluster that did not meet significance threshold for enrichment. Note, annotations of *gast* (see scRNA-seq methods), *tac4b* (see scRNA-seq methods, S15 Fig), and *sct* (see Results and Figs 2 and S6) were not previously identified and were defined in this paper. **(B–S)** Representative images from 6 days post fertilization (dpf) fish show subtype distribution and overlap with pan-EEC reporter *neurod1.* Individual cells are shown in inset. Quantitation of EEC subtype cell numbers from 3 to 6 dpf fish are also shown with each dot representing an individual fish. All scale bars are 100 μm. **(T)** 3 dpf, **(U)** 4 dpf, **(V)** 5 dpf, and **(W)** 6 dpf subtype localization data shown as frequency distributions of cells’ locations along the length of the gut divided into percentiles from proximal to distal. Distributions are smoothed for visualization. Underlying data can be found in S1 Data.

To understand how the seven zebrafish EEC populations we identified develop and function in vivo, we assembled reporters for the EEC progenitor population and five of the EEC subtypes (Fig 3A): *neurog3* and its transgene reporter *neurog3:QF2* for cluster 0, *ghrl* and its transgene reporter *ghrl:QF2* for cluster 1, *peptide YYb* (*pyyb*) and anti-PYY antibody for cluster 2, *glucagon a* (*gcga*) and its transgene reporter *gcga:GFP* for cluster 3, *cholecystokinin a* (*ccka*) and anti-CCK antibody for cluster 5, and *transient receptor potential cation channel, subfamily A, member 1b* (*trpa1b*) and its transgene reporter *trpa1b:GFP* for cluster 6. Transgenic reporters *gcga:GFP* and *trpa1b:GFP* and anti-PYY and anti-CCK antibodies were already available [99–102], but we developed new promoter-driven QF2 transgenic lines for *neurog3* (using a 2114 bp *neurog3* promoter) [103] and *ghrl* (using a 647 bp *ghrl* promoter) (S7 Fig and Methods). These reagents leverage the QF2-QUAS binary expression system, enabling us to combine our QF2 reporters with different QUAS tools [104,105]. As expected, our *ghrl* reporter co-labels with the *neurod1* reporter and labels sparse EECs in larval and adult fish (S7E–S7I Fig). Our *ghrl* reporter also co-labels with cells that stain with anti-Ghrelin antibody and cells that label with *ghrl* mRNA using hybridization chain reaction (S7J–S7M Fig). Our *neurog3* reporter likewise co-labels with the *neurod1* reporter and labels a similar number of cells in the intestine as previously reported lines generated with the same promoter (S7S and S7T Fig) [106].

To understand the dynamics of these EEC subtype reporters during intestinal development, we crossed each of them to the pan-EEC reporter *neurod1:RFP* [26] and imaged them daily from 3 to 6 days post fertilization (dpf) (S8 and S9 Figs). Cell counts from each day and representative images at 6 dpf are shown (Fig 3B–3S). For clarity, we refer below to cells labeled by a transgenic reporter or antibody marker as “+” for that respective gene (e.g., *ghrl*+). Subtype reporters showed a variety of prevalence and regional distribution patterns (Fig 3T–3W), which may contribute to their sub-specialized functions. *ghrl+,* PYY+, and CCK+ cells—labeling subtype clusters 1, 2, and 5, respectively—were all concentrated proximally within the intestine. Mammalian orthologs *Ghrl/GHRL* and *Cck/CCK* are also proximally skewed in the small intestine, but *Pyy/PYY* is more prevalent in the distal small intestine and colon in mammals [1,107]. *gcga+* cells from subtype cluster 3 peaked in the midgut, consistent with the enrichment of the transcription factor *osr2* in this cluster [72]. In accordance with mammalian literature, *trpa1b*+ cells labeling subtype cluster 6 (enterochromaffin cells) were the most prevalent subtype and were relatively evenly distributed along the length of the intestine [108]. To further explore the regional distribution of EEC subtypes, we used Seurat module scores to look at the expression of regionally enriched genes [109]. Consistent with our imaging results, midgut genes were enriched in cluster 3 and anterior gut markers were enriched across many subtype clusters (S10 Fig). Overall, the subtype reporters appeared as early as 3 dpf but became more widely expressed at 4 dpf, in alignment with *neurod1* reporter activity, growth of the intestine, and mRNA in situ hybridization data [29,30]. After 4 dpf, EEC subtype numbers increased with age except for *gcga+* and *ghrl*+ cells, which showed a trend towards decreasing after their initial increase in cell number at 4 dpf.

Importantly, while we apply these antibodies and reporters as useful markers of distinct EEC populations, EEC subtype definitions are operational and can vary across modalities and resolutions. For example, while expression of *sst2* and *ghrl* is highest in cluster 1 (S1 Table), cluster 1 contains cells that express only *sst2*, cells that express only *ghrl*, cells that express both *ghrl* and *sst2*, and cells that express neither *ghrl* nor *sst2* (S7N–S7R Fig). With greater clustering resolution and sequencing depth, these populations could be considered distinct subtypes. In this study, we operationally define cluster 1 as an EEC subtype and use the *ghrl* reporter as a label for this subtype.

### Ablation of *neurog3+* cells leads to reduction in EECs limited to select subtypes

We predicted that cells in scRNA-seq cluster 0 were EEC progenitors based on their enriched expression of *neurog3* and other EEC progenitor transcription factors and their early position on the pseudotime-predicted differentiation trajectory (Fig 1G and 1H). As cluster 0 made up a relatively small proportion of total EECs by scRNA-seq (Fig 1B), we were surprised to see our *neurog3* reporter label so many EECs in vivo (Fig 3B–3D). It is unclear if this is due to promiscuity of the *neurog3:QF2* transgene or perdurance of protein expressed from the transgene reporter. The mouse ortholog, *Neurog3*, is known to have transient expression in EEC progenitors [110], thus the perdurance of both QF2 and GFP may contribute to a long-lasting labeling of cells that transiently expressed *neurog3* in their developmental past. Thus, the unexpectedly high number of *neurog3*+ cells at 6 dpf may indicate that a larger portion of EECs transiently express *neurog3* at earlier developmental stages.

**Fig 4:**
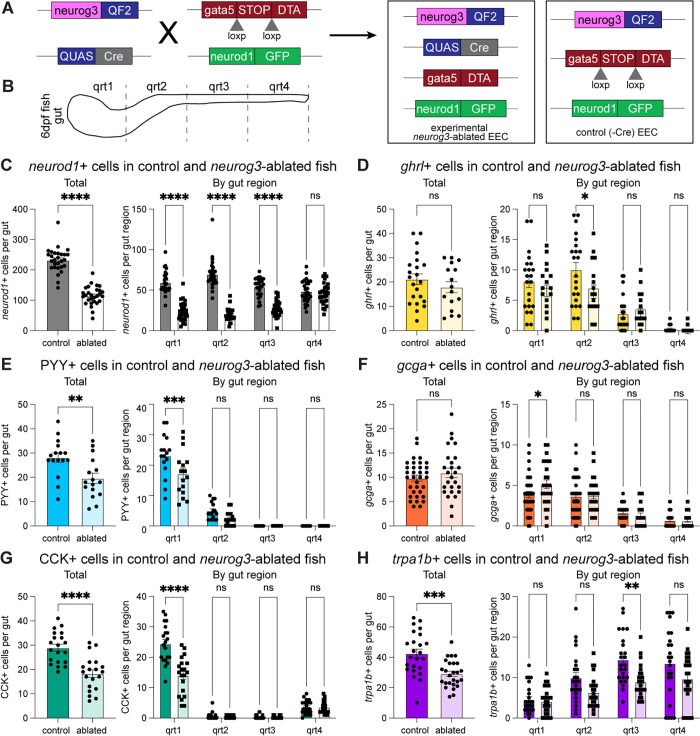
Selective ablation of *neurog3*+ cells in the intestine reduced total EECs and several EEC subtypes. **(A)** Schematic of *neurog3*+ cell ablation. **(B)** Schematic of regional scoring system. Total and regional counts of **(C)** *neurod1*+ cells, **(D)** *ghrl*+ cells, **(E)** PYY+ cells, **(F)** *gcga*+ cells, **(G)** CCK+ cells, and **(H)** *trpa1b*+ cells in control and *neurog3+* ablated fish are shown. Each dot represents a 6-day post-fertilization fish. Statistical significance was calculated by unpaired *t* test for total cell numbers and by two-way ANOVA for regional analysis. Significance annotations are as follows: ns (*p* > 0.05), * (*p* < 0.05), ** (*p* < 0.01), *** (*p* < 0.001), **** (*p* < 0.0001). Underlying data can be found in S1 Data.

To test this hypothesis, we used a combination of reporters to genetically induce Diphtheria toxin subunit A (DTA) expression, and thus cell death, in any *neurog3*+ cell in the intestine (Fig 4A). Specifically, we leveraged a transgene reporter that uses *gata5* regulatory sequences to drive intestinal epithelial expression of a loxP-flanked mCherry upstream of DTA (*gata5:RSD*) [27]. When we combined this reporter with our *neurog3:QF2* and *QUAS:Cre* alleles, the mCherry is recombined out in *neurog3*+ cells, leading to expression of DTA and cell death, which we operationally call “*neurog3+* cell ablation.” This strategy affects cluster 0, which has enriched *neurog3* expression, and effectively prevents the emergence of any further mature subtypes that ever pass through a *neurog3*+ state.

**Fig 5:**
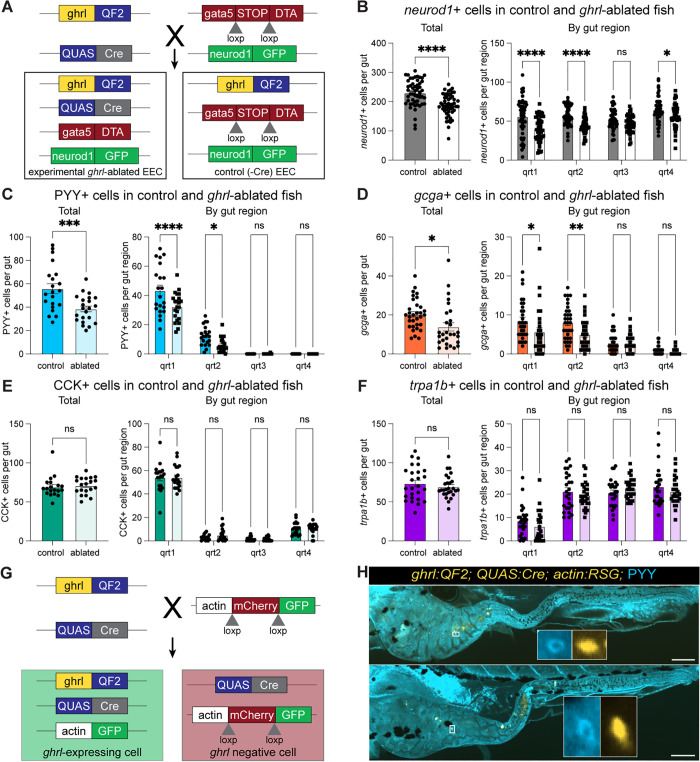
Selective ablation of *ghrl*+ cells suggests lineage relationships between *ghrl*+ cells and other EEC subtypes. **(A)** Schematic of *ghrl*+ cell ablation. Total and regional counts for **(B)** *neurod1*+ cells, **(C)** PYY+ cells, **(D)** *gcga*+ cells, **(E)** CCK+ cells, and **(F)** *trpa1b*+ cells in control and *ghrl*-ablated fish are shown. Each dot represents a 6-day post-fertilization fish. **(G)** Schematic of *ghrl+* cell lineage tracing. **(H)** Representative images of *ghrl* lineage tracing with PYY staining showing rare double-positive cells. Scale bars are 100 μm. Statistical significance was calculated by unpaired *t* test for total cell numbers and by two-way ANOVA for regional analysis. Significance annotations are as follows: ns (*p* > 0.05), * (*p* < 0.05), ** (*p* < 0.01), *** (*p* < 0.001), **** (*p* < 0.0001). Underlying data can be found in S1 Data.

We evaluated the total numbers and regional distribution of *neurod1*+ cells (Fig 4B) in this *neurog3+* cell ablation model and found that roughly 50% of all EECs are lost with the effect being more pronounced in the proximal gut (Fig 4C). A previous study showed that expression of the *gata5:RSD* transgene is relatively higher in the proximal region of the larval intestine and that combination with *neurod1:Cre* (*neurod1*+ cell ablation) led to ablation of EECs in the proximal and mid intestine while retaining some EECs in the most distal region [27]. To test if this regional activity of the *gata5:RSD* transgene contributed to our observed patterns of regional EEC loss following *neurog3*+ cell ablation, we performed a series of control experiments. First, we quantified the number of *neurog3*+ cells in the *neurog3*-ablated fish and found that while almost no *neurog3*+ cells remained in the first three quarters of the intestine (qrt1–3), levels of *neurog3*+ cells were comparable to unablated control fish in the fourth quarter of the gut (qrt4; S11A and S11B Fig). We then likewise evaluated the effects of *neurod1* ablation on *neurod1*+ cells and observed a similar near complete loss of *neurod1*+ cells in qrt1–3 and preservation of *neurod1*+ cells in qrt4, consistent with previous studies [27]. In comparing the *neurod1*+ cell numbers in control, *neurog3*-ablated, and *neurod1*-ablated fish, there were significantly fewer *neurod1*+ cells in the proximal three-quarters of the gut in the *neurod1*-ablated fish as compared to the *neurog3*-ablated fish (S11C Fig). Similarly, *trpa1b*+ cells, while preserved in qrt4 in all animals, were significantly more reduced in *neurod1-*ablated than *neurog3*-ablated fish (S11D Fig). Together, these experiments show that combining a Cre driver with *gata5:RSD* efficiently kills cells in the first three quarters of the larval gut. They furthermore demonstrate that *neurog3*+ ablation significantly reduces EEC numbers, but not to the same extent as *neurod1* ablation, supporting the conclusion that, in contrast to mammalian systems [10], a *neurog3+* cell state is not required for all EEC development in zebrafish. These results also demonstrate that ablation using the *gata5:RSD* transgene is inefficient in the final quarter of the gut, likely due to lower *gata5* expression in that region. To account for that lack of ablation in qrt4, we calculated total EEC numbers in our ablation experiments both including qrt4 (Figs 4C–4H and 5B–5F) and excluding qrt4 (S11E and S11F Fig) and observed the effect of ablation was the same regardless of whether qrt4 cells were included or not.

We next combined our cell ablation strategy with EEC subtype reporters to ask if certain EEC subtypes were particularly affected by *neurog3*+ cell ablation. Because we could not combine the *neurog3:QF2* and *ghrl:QF2* reporters, we generated a *neurog3:Cre* transgenic line to test the effects of *neurog3*+ cell ablation on *ghrl*+ cells. We confirmed that *neurog3+* cell ablation induced with the *neurog3:Cre* reporter similarly reduced *neurod1*+ cell numbers as compared to the *neurog3:QF2; QUAS:Cre* reporter (S12 Fig). When evaluating EEC subtype populations in the context of *neurog3*+ cell ablation, we found that PYY+, CCK+, and *trpa1b+* cells were all reduced by roughly 30% while *gcga+* and *ghrl*+ cells were unaffected (Fig 4D–4H). Consistent with our reporter characterization (Fig 3), reductions in EEC subtypes were observed in the region of the gut where they were most prevalent—proximally for PYY+ and CCK+ cells and distally for *trpa1b*+ cells. This suggests that a portion of PYY+, CCK+, and *trpa1b+* subtypes pass through a *neurog3+* cell state during their differentiation.

To test if the effects of *neurog3+* ablation were related to the loss of the *neurog3* gene product, we quantified the number of EECs in *neurog3* mutants [29] and control animals and observed no difference (S13D Fig). This suggests that the *neurog3* gene is not required for zebrafish EEC differentiation, which is consistent with what has been reported in zebrafish pancreatic islet differentiation [29], but contrasts with the known essential role of *Neurog3* in mammalian EEC development [10]. In combination with the results of the *neurog3+* ablation experiments, we conclude that many zebrafish EECs pass through a *neurog3*+ cell state, but do not require *neurog3* function for their overt development.

### Ablation of *ghrl+* cells leads to reduction in EECs in subtypes distinct from *neurog3*+ ablation

The persistence of *ghrl+* cells despite *neurog3+* cell ablation is reminiscent of mammalian data that shows *Ghrl* expression and *Ghrl*+ cell numbers were maintained or increased upon deletion of several transcription factors important for EEC differentiation, such as *Nkx2.2a* and *Pax4* [19,65,66,111–114]. In addition, our pseudotime analysis predicted a differentiation trajectory moving through *ghrl*+ cells, strikingly similar to several mammalian scRNA-seq datasets that suggested a *Ghrl*+ EEC progenitor population [23–25,115]. Older lineage tracing data also supports a role for *Ghrl*+ cells as progenitors of other endocrine populations in the stomach and pancreatic islet [22]. However, the hypothesis that *Ghrl*+ cells in the intestine give rise to other EEC subtype populations remained untested.

Using a similar genetic approach as above, we used *gata5:RSD* to selectively kill any *ghrl*+ cell in the intestinal epithelium, which we operationally define as “*ghrl+* cell ablation” (Fig 5A). Despite *ghrl*+ cell numbers peaking at approximately 15 cells per animal during larval development (Fig 3G), we found *ghrl*+ cell ablation resulted in a loss of roughly 40 EECs, indicating other EEC subtypes might be affected (Fig 5B). Indeed, we found a 30% reduction in PYY+ and *gcga*+ EECs in *ghrl*-ablated fish while CCK+ and *trpa1b*+ cell numbers remained unaffected (Fig 5C–5F). Notably, this is distinct from the *neurog3+* ablation result where PYY+, CCK+, and *trpa1b*+ cells were reduced and *ghrl+* and *gcga+* cell numbers were unaffected (Fig 4). These data suggest a *neurog3*-independent role for *ghrl*+ cells in the differentiation of PYY+ and *gcga*+ EEC subtypes. This could be explained by PYY+ and *gcga*+ cells passing through a *ghrl*+ cell state or by the loss of a secreted factor from *ghrl*+ cells that promotes differentiation of these subtypes.

To distinguish these possibilities, we first used lineage tracing tools to indelibly label a cell with GFP if it has ever expressed *ghrl* (Fig 5G). We then performed PYY staining and found rare occurrences of double-positive cells, confirming that some PYY+ cells have indeed passed through a *ghrl*+ cell state (Fig 5H). This suggests that the impact of *ghrl+* cell ablation on PYY+ cells is at least in part cell autonomous. Due to overlapping fluorophores, we were not able to perform a similar study with *gcga*+ cells.

Given the small number of *ghrl*-derived PYY+ cells in our lineage tracing analysis, we hypothesized that *ghrl*+ cells could also be regulating other EEC subtypes non-cell autonomously through secreted products. As ghrelin peptides were prevalent in our peptidomics dataset (Fig 2D) and have been shown to stimulate growth of intestinal cells [116,117], we tested if loss of the *ghrl* gene impacted EEC development. We used CRISPR/Cas9 to generate *ghrl* mutant zebrafish and evaluated the effects on *neurod1*+ EECs. We found that EEC numbers were equivalent across *ghrl* mutant and wildtype fish (S13 Fig), suggesting that loss of *ghrl* gene function does not overtly impact EEC development.

### Ablation of *neurod1*+ cells, but not *ghrl+* or *neurog3+* cells, impacts adult survival and growth

To test the ramifications of each of our EEC ablation models on zebrafish growth and survival, we raised ablated fish with their sibling controls to adulthood. We maintained at least five tanks of each control and ablated condition for each experimental replicate and checked survival at several timepoints. At the end point of each study, we also randomly selected fish in each group for measurement of standard length (S14A and S14B Fig) as a metric of animal growth [118]. As we anticipated, *neurod1*+ ablated fish showed the most severe phenotype, with evidence of reduced survival as early as 2 weeks post fertilization (S14C Fig). Additionally, at the 6-week endpoint, *neurod1+* ablated fish were also observed to be smaller than their sibling controls (S14C Fig). In contrast, neither *neurog3+* nor *ghrl*+ ablated fish showed survival or growth defects over 6 weeks (S14D, S14F Fig). To further interrogate any possible differences, we raised a second set of *neurog3*+ and *ghrl*+ ablated and control fish to 12 weeks post fertilization, a commonly accepted time point for full maturity [118]. However, we still did not observe any differences in survival or growth (S14E, S14G Fig), suggesting *neurog3*+ and *ghrl*+ ablation have no significant impact on feeding behavior and adult development.

## Discussion

Here, we report seven distinct EEC populations in zebrafish and characterized reporters for six of them. Our characterization of EEC subtype reporters is consistent with previously published larval zebrafish in situ hybridization data, building confidence in our proposed EEC subtype identities [28,30]. These studies agree with our data that first onset of these genes is around 3 dpf (72 hpf) with expression increasing by 4 dpf [28,30]. While PYY and CCK antibody staining cells were rare at 3 dpf in our study, *pyyb* and *ccka* in situ hybridization data showed a greater number of positive cells [30], suggesting that protein expression lags slightly behind transcript presence, as might be anticipated. Despite this slight difference, the regional localization of these cells across all timepoints and the number of positive cells at 4 dpf were consistent between our observations and published studies [28,30]. Intriguingly, prior in situ hybridization analysis of genes that we report here as expressed in the same EEC subtype showed the same prevalence and regional patterns, consistent with them being present in the same cells. For example, EEC subtype cluster 1 in our scRNA-seq analysis expressed *ghrl*, *gip*, *mlnl*, and *sst2* and was labeled in this study with a *ghrl* transgene reporter. Both our *ghrl* transgene reporter and published in situ data for *ghrl*, *gip*, *mlnl*, and *sst2* labeled <10 cells in the proximal gut at 4 dpf. Furthermore, previous studies showed *ghrl* and *mlnl* in situ probes overlap, as would be predicted from our data [30]. Additionally, our identification of EEC cluster 6 as enterochromaffin cells is consistent with previous reports of colocalization of enterochromaffin markers *trpa1b*, *tph1b*, serotonin, and enkephalin (a product of *penka*) in a subset of zebrafish EECs that communicate with the nervous system [27,119]. Finally, our analysis of zebrafish EECs in a separate scRNA-seq dataset showed subtypes with very similar EEC hormone distributions (S3 Fig). This consistency in EEC subtypes across many datasets and modalities builds confidence in our findings.

We show larval and adult EECs have remarkably consistent subtype signatures (Figs 1D, 1E, 1G, and S2D–S2F), suggesting that EEC subtype programing is established in larvae prior to feeding and persists through adulthood. Similar studies across ages in other animals are limited. One scRNA-seq study of the human intestine looked at EECs from adult, pediatric, and fetal tissue samples. Although a comparison across subject age was limited by 90% of the EECs coming from fetal tissue, the cells did not clearly separate by age. Instead, as in our study, clustering was largely driven by hormone expression [75]. Additional studies of mouse and human fetal EECs have likewise shown hormone expression patterns similar to those reported from adult tissue [51,115,120,121]. This suggests that the establishment of durable EEC subtype programs during early development may be a common feature of EECs in fishes as well as mammals.

The repertoire of hormone coding genes expressed in EECs is very similar between zebrafish and mammals (Fig 1F), despite over 400 million years having elapsed since their last common ancestor [122]. We found that orthologs of most commonly reported mammalian EEC hormone coding genes were expressed in our scRNA-seq dataset, with the exceptions of *PPY*, *IAPP*, and *NPY*. *PPY* does not appear to have an ortholog in zebrafish. *IAPP* has an orthologous novel gene that has not been well described in zebrafish, and *NPY* similarly has a zebrafish ortholog, but has largely been characterized in the brain [123,124]. Conversely, two EEC hormone coding genes were found in our zebrafish dataset that have not been commonly reported in mammalian EECs, namely *pdyn* and *tac4b*. *PDYN* and *TAC4* are present in mammals but have not yet been reported in EECs. Despite these small differences, the remarkable conservation of the overall EEC hormone gene repertoire between zebrafish and mammals highlights the critical roles of these signaling molecules during vertebrate evolution.

Additionally, zebrafish orthologs for *GASTRIN* (*GAST*) and *SCT* were previously not identified and were annotated in this study. Despite Bayliss and Starling reporting in 1903 that extracts from salmon intestine have secretin activity [125], studies since then have repeatedly failed to identify secretin orthologs in fishes via sequence homology or synteny analyses [126–130]. Secretin is a member of the PACAP/GCG family of peptides that likely originated from a single exon present over 600 million years ago before the two rounds of whole genome duplication that occurred during the vertebrate radiation [128,129,131–135]. Interestingly, many genes in that family encode two non-overlapping peptides, but mammalian *SCT* is believed to have lost its second C-terminal peptide [135]. Here, we show that there is a region of the Secretin protein C-terminal to the annotated secretin peptide that is highly conserved across fishes and produces highly processed peptides in zebrafish (gray shading in Fig 2F). This suggests that mammals and fishes each maintained only one peptide from the ancestral homolog, with the fish peptide sequence differing enough from the ancestral sequence as to be missed on previous homology searches [126–130]. The recent efforts to sequence genomes of species like gar (*Lepisosteus oculatus*) [95] and elephant shark (*Callorhinchus milii*) [96] allowed us to connect the unannotated zebrafish *sct* gene to more ancient sequences and ultimately identify it as a *sct* ortholog. Intriguingly, *sct* in chicken and other birds has been shown to encode two peptides, both of which were able to bind to and stimulate the chicken secretin receptor SCTR in cultured mammalian cells [136]. The possible in vivo role of these *sct*-derived peptides in birds is unclear, however, as chicken *SCTR* is lowly expressed in the pancreas (where it is known to be enriched in mammals) but more highly expressed in intestine and testes [136], offering other possible sites of action. Together, these data suggest that the second C-terminal Secretin-derived peptide presumed lost in mammals may still be expressed and functional in other vertebrate species. Further studies investigating the roles of both possible peptides encoded by zebrafish *sct* are warranted.

Our peptidomic analysis identified peptides aligning to 19 of the 23 hormone coding genes expressed in our scRNA-seq data, including the newly-identified zebrafish *sct*. Of note, the liquid chromatography and mass spectrometry settings used to generate our EEC peptidomic dataset may have slightly biased the peptides we were able to detect. For example, as in many standard protocols, singly charged peptides were not selected for fragmentation, meaning that we would not identify many Leu and Met enkephalin peptides that might be derived from *proenkephalin a* (*penka*) or *pdyn*. *CCK*-derived peptides have also been shown to be singly charged in human studies, and, for that and several other reasons, have proven exceedingly difficult to identify by LC–MS/MS [137]. Furthermore, the complexity of peptidomic samples, including the high charge states and complex fragmentation, complicate the detection of longer peptides, although peptides up to 60 amino acids long have been reported using similar techniques [138,139]. Additionally, as noted in the Results, the atlas here reports diversity of unique peptides produced from each gene, not their relative natural abundance in EEC secretory granules. Finally, while we analyzed both larval and adult samples, we did not perform quantitative comparison between the two groups. While peptides from adult samples are over-represented in our results (S2 Table), this is likely because the total cell content in adult samples was almost twice that of larval samples (50,000 versus 30,000 cells) due to limitations in sorting and collecting EECs from larvae. As such, for peptides not detected in larval samples, we cannot conclude that they were absent as they may have been below the limit of detection. For this reason, we did not perform direct comparisons between larval and adult samples here. This and other questions raised by our work here would be assisted by the development of antibodies against zebrafish EEC peptides.

We hope the zebrafish EEC peptides reported here will be leveraged for further discovery related to EEC hormone endocrinology, and we refer the reader to the supplemental materials for a complete catalog of the detected peptides (see S4 Fig and S2 Table). There are exciting possibilities for discovery related to the peptides we report here that show homology to known human hormones. Historically, studies of homologous peptide hormones in non-mammalian models have provided critical insights into hormone function and even informed therapeutic design. For example, the first identification of GLP-1 occurred in anglerfish [140], and exendin-4, a peptide hormone produced by gila monster lizards that is analogous to human GLP-1, contributed to the development of long-acting GLP-1 analogs now broadly used for treatment of diabetes and obesity in humans [141–143]. We detected several zebrafish peptides that map to the regions of *gcga* and *gcgb* that align with the GLP-1 encoding portion of human *GCG* (S4B Fig). The arginine 36 residue in GLP-1 is commonly amidated in mammals [144,145], as is seen with one of the *gcgb*-derived peptides detected in zebrafish. However, there are also notable differences between zebrafish *gcga/gcgb* and human *GCG* and their corresponding peptides. Firstly, *gcgb* and isoform 2 of *gcga* appear to have lost the GLP-2 coding region, a phenomenon also reported in other fishes [146,147]. Additionally, the GLP-1 region in zebrafish Gcga lacks the highly conserved dibasic PCSK1/2 cleavage site at its C-terminus [148], suggesting alternative processing of this peptide, perhaps at the dibasic RR site immediately upstream of the GLP-2 region. This would result in a 45 amino acid peptide, which would be more challenging to detect and might explain why, although there are many detected peptides aligning to the GLP-1 region in zebrafish, there is not one that perfectly overlaps with the predicted GLP-1 sequence. These nuances in peptide structure and sequence could provide valuable insight into the evolution, function, and regulation of these hormones.

Beyond the few peptides discussed in the Results above, we also detected peptides aligning to regions annotated in humans as coding for peptide hormones like pituitary adenylate cyclase-activating polypeptide (PACAP), glucagon, glicentin-related polypeptide, calcitonin, katacalcin, galanin, insulin-like 5 (INSL5) A and B chain, motilin, neuromedin B, proenkephalin, Leu and Met enkephalins, synenkephalin, neuronostatin, and somatostatin. These could be exciting candidates for future studies into EEC peptide hormone biology. There are also opportunities to potentially uncover novel signaling molecules among the peptides that are abundant in zebrafish but align to a region with no annotated function in humans. For example, recent studies have illustrated how new technologies and datasets such as this can continue to reveal novel peptide hormones [77,78,149,150]. While these are a few examples, we hope the entire zebrafish EEC peptide catalog will be a resource for future discovery related to EEC hormones (S4 Fig, S2 Table).

We also detected peptides derived from *glucagon b, somatostatin 1.2,* and *insulin* (S4B, S4M, and S4P Fig), hormone genes that are commonly thought to be enriched in the pancreatic islet [30,151,152]. While this could be evidence of pancreatic contamination in our sorted EEC samples, those genes do show some limited expression in the joint larval and adult EEC dataset (S5E Fig) and have been reported in zebrafish intestinal bulk RNA-seq datasets before [30,153], suggesting these peptides might be produced in zebrafish EECs.

Despite striking conservation of EEC hormone coding genes and peptides, the partitioning of those genes into EEC subtypes shows notable differences between zebrafish and mammals. Specifically, while *gip* and *nts* are present in zebrafish EECs, *gip* or *nts*-expressing cells do not separate into their own clusters as they often do in mammalian datasets, perhaps because they are expressed at relatively lower levels in zebrafish [19,52,53]. Additionally, while *PYY* and *GCG* are found together in what have traditionally been called “L cells” in mammals, we see their orthologs *pyyb* and *gcga* separate into clusters 2 and 3, respectively, in our dataset. Of note, cluster 2 and 3 are somewhat intermingled in the UMAP plot and have the most overlapping marker profile of any two clusters (Figs 1G and S2D–S2F), suggesting a close relationship between the two populations. Furthermore, scRNA-seq reports from mouse colon, where *Pyy* is more highly expressed, have separated *Pyy* and *Gcg* into separate clusters [107]. Finally, we also observed high expression of *insulin-like 5* paralogs *insl5a* and *insl5b* in our dataset. The mammalian homolog *INSL5* is mostly restricted to colonic EECs [51,54,107,154]. As there is limited homology between posterior regions of the zebrafish intestine and the mammalian colon [155–157], *insl5a+* and *insl5b*+ cells likely have a different distribution in zebrafish than they do in mammals. These findings suggest that some differences in EEC subtype identities between zebrafish and mammals may be due to sub-specialization of intestinal regions in mammals.

Our results indicate that *neurog3* has a smaller role in EEC differentiation in zebrafish than in mouse. *Neurog3* has been shown to be necessary and sufficient for committing secretory progenitors to an endocrine fate in both pancreatic islet endocrine cells and EECs in mammals [10–14,158–160]. Although *neurog3* was shown to be dispensable for islet development in zebrafish, its role in EECs had not been formally tested [29]. We observed a more minor role for *neurog3+* cells in EEC differentiation in zebrafish where only some EEC subtypes—namely *trpa1b+,* CCK+, and PYY+ cells—seem to pass through a *neurog3*+ cell state. This contrasts with our pseudotime data, which predicted that most EEC subtypes, and specifically *ghrl*+, PYY*+,* and *gcga+* populations, pass through an EEC progenitor population enriched for *neurog3* expression (Fig 1H). This discrepancy could be because *neurog3* labels only a portion of our putative EEC progenitor population, such that some EEC progenitor cells in cluster 0 are unaffected by *neurog3* ablation and are still able to differentiate into EEC subtypes. Alternatively, the more limited role of *neurog3*+ cells we observed in vivo could be because cell intermediates of alternative differentiation trajectories from secretory progenitors to differentiated EEC subtypes might have been poorly captured in our scRNA-seq data. For example, a few secretory progenitor cluster 8 cells cluster with *ghrl*+ cluster 1 cells, raising the possibility that there could be a path from cluster 8 to cluster 1 that does not pass through *neurog3*+ cells in cluster 0. Finally, cell ablation studies always carry the potential caveat of compensation, in which removing one population makes room for another to at least partially take its place. Despite these methodological limitations, our in vivo studies demonstrate that ablation of *neurog3*+ cells only impacts a subset of EEC subtype populations. This partial effect of *neurog3+ *cell ablation is consistent with published *neurog3* in situ hybridization data [28] and findings that support *neurod1* as a major fate-determining transcription factor in zebrafish EECs [27,31].

Prior studies in mammals raised the intriguing possibility that *Ghrl-*expressing EECs may have a role in the differentiation of EEC progenitors into mature EEC subtypes, but this had not been directly explored. Reports in mice showed *Ghrl* expression and *Ghrl*+ cell numbers were uniquely resistant to deletion of transcription factors important for EEC differentiation, such as *Nkx2.2* [65–67], *Arx* [112,161], *Pax4* [112,114], *Sox4*, *Tox3*, [19] *Myt1* [19], and *Isl1* [113]. While *Ghrl*+ cell numbers were maintained or often increased upon loss of those transcription factors, almost all other EEC subtype markers were reduced. Notably, our zebrafish data showing *ghrl*+ cells were unaffected by *neurog3*+ cell ablation is consistent with this pattern. Altogether, these data support two possible models to explain why *Ghrl*+ cells are uniquely enriched upon loss of various transcription factors important for EEC differentiation: 1) *Ghrl*+ cells are an early-arising mature EEC fate that becomes the default destination for progenitors in the absence of key transcription factors; or 2) *Ghrl*+ cells themselves give rise to other subtypes through the subsequent expression of key transcription factors, and, when those transcription factors are lost, EECs become arrested in a *Ghrl*+ state. Data from different scRNA-seq studies in mammalian EECs are consistent with both models. Computational analysis of time series scRNA-seq data from mouse organoids show that *Ghrl*+ cells are some of the first to arise [19], supporting the first model. However, additional scRNA-seq studies have identified “*Ghrl*+ EEC progenitors.” These cells express progenitor markers, including transcription factors important for EEC differentiation, but also express *Ghrl*, thought to be a mature EEC marker [23–25]. As in our study, one of these studies found a predicted pseudotime differentiation trajectory moving through a *Ghrl*+ EEC progenitor state [24], supporting the second model. Interestingly, studies reporting *Ghrl*+ EEC progenitors also report *Ghrl* expression in later, more mature EEC populations, suggesting the possibility of a hybrid model with two distinct *Ghrl*+ populations, only one of which serves as a progenitor. Despite the preponderance of suggestive evidence, however, a role for *Ghrl*+ cells in EEC differentiation in the intestine has not, to our knowledge, ever been formally tested.

In this study we show that ablation of *ghrl+* cells impacts other EEC subtypes, namely *gcga*+ and PYY+ cells, and that *ghrl*+ and PYY+ cells have a lineage relationship, in line with model 2 proposed above. Lineage relationship between *ghrl*+ and *gcga*+ cells could not be tested here due to lack of an antibody labeling *gcga*+ cells. While we acknowledge that lineage-based approaches have limitations and can confuse lineage dependency and co-expression, these data are supportive of a role for *ghrl*+ cells in the differentiation of other subtypes. Interestingly, while lineage tracing of *ghrl*+ cells revealed rare instances of overlap with PYY, these events were not frequent enough to fully explain the reduction of PYY cells seen in *ghrl+* ablated fish. As EECs are known to signal to each other [162–166], it is possible that loss of the signals secreted from *ghrl*+ cells is the primary driver of reductions in other EEC subtypes upon *ghrl+* cell ablation. We did not see any effect on total EEC number following *ghrl* deletion, however, suggesting that loss of ghrelin signaling is not the cause.

**Fig 6:**
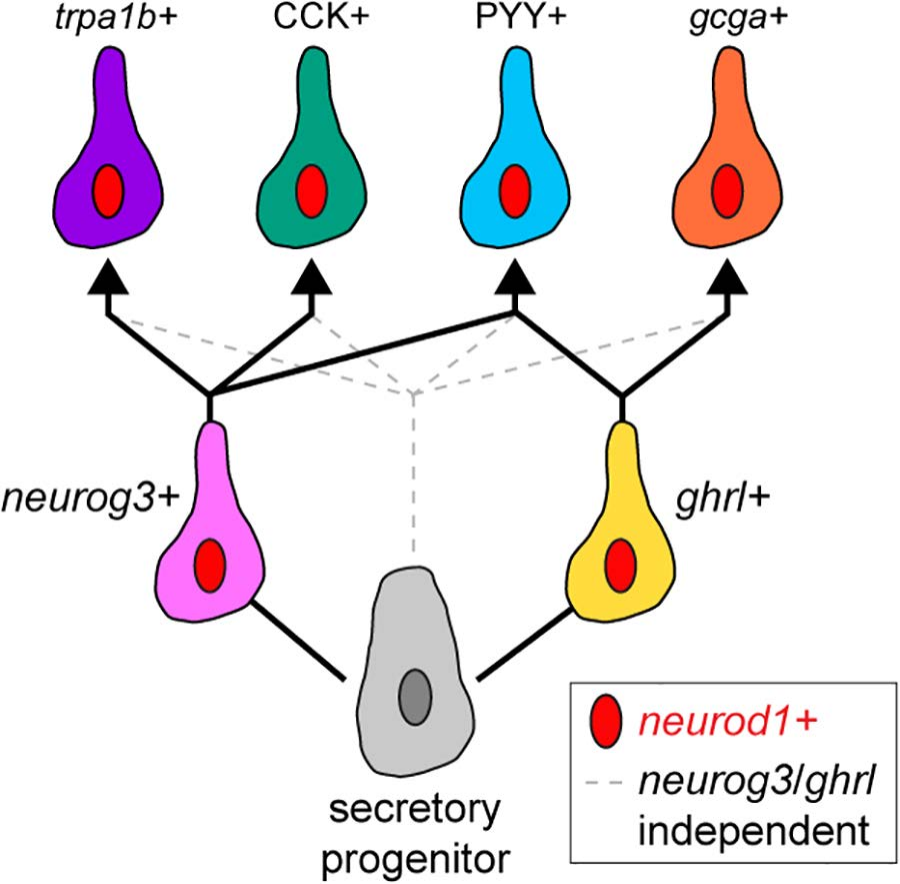
Working model of EEC subtype differentiation in zebrafish.

Our findings here together with the existing literature support a new model of zebrafish EEC differentiation (Fig 6). EECs are derived from secretory progenitors and are marked by the expression of *neurod1,* which is required for all EECs in zebrafish [27,31]. Our findings here support that a portion of *trpa1b+,* CCK+, and PYY+ EEC subtypes pass through a *neurog3+* cell state and a portion of *gcga*+ and PYY+ EECs pass through a *ghrl*+ cell state. Both *neurog3+* and *ghrl+* cell ablation had only partial impacts on other EEC subtype populations, indicating *neurog3-* and *ghrl-*independent differentiation pathways are also available. Multiple differentiation pathways may contribute to EEC subtype plasticity or compensation, but it is unclear if there are functional differences between cells of the same subtype that differentiate through varying pathways. We also acknowledge that ablation of *ghrl*+ or *neurog3*+ cells may have fundamentally shifted the transcriptional profile of EEC subtype populations. While we evaluated the numbers of cells labeling with each subtype reporter, we do not know if the subtype biology beyond that single reporter is preserved.

An important limitation of this study is its reliance on transgenic reporters to label and ablate EEC subtypes. Most of the transgenic lines used here, most notably those for *neurog3* and *ghrl*, use short promoter sequences from those loci that may not perfectly reflect the expression patterns of the respective endogenous genes. Further, use of the QF2-QUAS system is prone to perdurance of QF2 after the promoter driving its expression is no longer active. As mentioned in the Results, this caveat is particularly apparent in the disparity between the large number of cells labeled by our *neurog3:QF2* transgene and the relatively few cells that express *neurog3* in our scRNA-seq dataset (Figs 1E and 3B–3D). Thus, as with most lineage tracing studies, our findings using these reagents suggest developmental relationships that should be validated in the future using additional technologies.

In conclusion, the tools and datasets reported here can serve as a platform for future hypothesis testing to better understand fundamental EEC subtype and hormone biology. For example, our atlas of endogenous EEC peptides can serve as a starting point for testing their individual endocrine functions and structure-function relationships. The toolkit of EEC subtype reporters also provides a valuable resource for further in vivo studies. The modularity of the QF2-QUAS transgenic tools deployed here will allow future studies to not only label, but selectively activate or inhibit certain subtypes to understand their physiologic roles. Future work could test the subtype-specific impacts of various challenges such as dietary changes, microbial manipulations, or disease models.

## Materials and methods

### Ethics statement

All zebrafish studies were approved by the Institutional Animal Care and Use Committees of Duke University (protocol A061-22-03) and Michigan State University (protocol PROTO202200367).

### Zebrafish lines and husbandry

Zebrafish stocks were maintained on an Ekkwill (EK) background on a 14-hour/10-hour light/dark cycle at 28.5°C in a recirculating aquaculture system (Pentair). From 6 to 14 dpf, larvae were fed Zeigler AP100 <50-micrometer larval diet (Pentair, LD50-AQ) twice daily. Beginning at 14 dpf, larvae were fed *Artemia* (Brine Shrimp Direct, BSEACASE) once daily, supplemented with a daily feed of Skretting Gemma Micro 75 (Bio-Oregon, B5676). From 28 dpf, the Gemma Micro 75 diet was replaced with Gemma Micro 300 (Bio-Oregon, B2809). At the onset of breeding age or sexual maturity, adult fish were given a 50/50 mix of Skretting Gemma Micro 500 (Bio-Oregon, B1473) and Skretting Gemma Wean 0.5 (Bio-Oregon, B2818) and one feeding of *Artemia* daily.

For experiments involving zebrafish larvae, adults were bred naturally in system water and fertilized eggs were transferred to 100 mm petri dishes containing ~60 mL of egg water at approximately 6 hours post-fertilization. The resulting larvae were raised under a 14-hour light/10-hour dark cycle in an air incubator at 28°C at a density of 1 larvae/mL water. All the experiments performed in this study ended at 6 dpf unless specifically indicated. As sex determination in zebrafish occurs after 6 dpf, we do not report numbers of male or female fish in each experiment. The following engineered zebrafish lines were used in this study:

*TgBAC(cldn15la:EGFP)*^*pd1034*^ [167], *Tg(neurod1:TagRFP)*^*w69*^ [168], *TgBAC(trpa1b:EGFP)*^*a129*^ [102], *TgBAC(gata5:loxp-mcherry-stop-loxp-DTA)*^*pd315*^ [27], *TgBAC(neurod1:EGFP)*^*nl1*^ [169], *Tg(gcga:EGFP)*^*ia1*^ [170], Tg(*β-actin2:loxP-mCherry-loxP-GFP*)^*pd31*^ [171], *Tg(QUAS:GFP; cryaa:mCherry*)^*pd1199*^ [172], *neurog3*^*sa211*^ [29]*, Tg(neurog3:QF2; cmlc2:GFP)*^*rdu107*^ (generated in this study), *Tg(ghrl:QF2; cmlc2:GFP)*^*rdu108*^ (generated in this study), *Tg(QUAS:cre; cryaa:mCherry)*^*rdu109*^ (generated in this study), *Tg(neurog3:Cre; cmlc2:GFP)*^*rdu110*^ (generated in this study), *ghrl* ^*rdu111*^ (generated in this study), and *Tg(QUAS:TagBFP)*^*rdu113*^ (generated in this study).

### Construction of zebrafish lines

The Gateway Tol2 cloning approach was used to generate *Tg(neurog3:QF2; cmlc2:GFP), Tg(ghrl:QF2; cmlc2:GFP), Tg(QUAS:cre; cryaa:mCherry),* and *Tg(neurog3:Cre; cmlc2:GFP)* plasmids [103]. The 2114 base pair *neurog3* promoter was previously reported [106] and generously shared by Dr. Olov Andersson (Karolinska Institutet) and cloned into a p5E-MCS vector [103] using TAKARA In-Fusion Snap Assembly master mix (Takara, 638947) with HindIII-linearization of the backbone and amplification of the insert with primers CGGTATCGATAAGCTCCGCGGCCGCCGTACTCGA and ATTCGATATCAAGCTCGGCGCGCCCACCCTTTCTGTA. The pME-QF2 [105], p5E-QUAS [173], p3E-polyA [103], pDestTol2CG2 [103], and pDESTtol2pACrymCherry [174] plasmids were previously reported and obtained from Addgene. The pME-Cre plasmid was previously reported [175] and generously shared by Dr. Mark Cronan. The 647 base pair *ghrl* promoter was amplified from purified (Wizard Genomic DNA purification kit, A1120) EK DNA using primers cttaagcttTTCAGAATTCATATCAGATCACAGACACT, cttccgcggCACCTTAGTCTTAATTCTTTGCTACATAC. It was then cloned into the p5E-MCS [103] via digestion of insert and backbone with HindIII (NEB, R3104S) and SacII (NEB, R01575S) followed by phosphatase treatment (NEB, M0289S) and ligation (NEB, M0202S). The appropriate p5E, pME, and p3E entry vectors were cloned into destination vectors with either a green heart (*cmlc2:GFP;* pDestTol2CG2) or red eye (*cryaa:mCherry*; pDESTtol2pACrymCherry) as listed above using an LR-Clonase (ThermoFisher, 12538-120) reaction.

The *ghrl* mutant line was generated using CRISPR-Cas9. The guide RNAs (gRNAs) were designed using the “CRISPRscan” tool (www.crisprscan.org/) [176] and purchased fully synthesized from Integrated DNA Technologies (see S4 Table). At the one-cell stage, EK strain zebrafish embryos were injected with 1–2 nL of a cocktail consisting of Cas9 protein (1,000 ng/μL), gRNA (150 ng/μL), and 0.05% phenol red. Injected embryos were screened for mutagenesis with forward primer CACAGTGACAGTTGTAGACTTTAATGCTAAT and reverse primers GTCTCTAAGAAGATTCTCCAGAAGATTCTGA (mutant), AAACATGCTGCTGGCACGGCA (wildtype). The mutations were further determined through Sanger sequencing of the region encompassing the gRNA targeting sites. Ten F0 injected fish were out-crossed to wild-type fish and progeny were screened to test if mutations were transmissible. F1s from F0 founders were then raised to adulthood and sequenced. Two F1s were found to have identical lesions—the deletion started after the first nucleotide in the first exon and continued through the first 17 nucleotides of the third exon—and were bred to generate a stable line. F2s and F3s from this line were used in the reported experiments and were given the allele name *rdu111*. When testing *ghrl* mutants, genotyping of the *mttp* locus was used as a control to verify there was viable DNA in samples using forward primer AGAGACGGTGTCCAAGCAGG and GCTCAAAGACTTTCTTGC with an expected band of 137 base pairs [177].

### mVISTA alignment

To identify conserved regions of the *ghrl* promoter across *Danio* species, we exported the *ghrl* sequence and annotation from ensembl.org [97]. We blasted the *D. rerio* Ghrl amino acid sequence against other *Danio* species and extracted 5 kb upstream and downstream of the aligned region for each species. We then put each of these sequences into the mVISTA alignment [178,179], using the *D. rerio* sequence and annotation as the reference. We identified and amplified a 647 base pair region upstream of the *ghrl* transcriptional start site that was highly conserved across *Danio* species (S7B Fig).

### Larval immunofluorescence and live imaging

All larval imaging was performed on Andor Dragonfly Spinning Disc Confocal plus with a 20× lens in the Duke Light Microscopy Core Facility. For live imaging experiments, zebrafish larvae were anesthetized with tricaine and mounted in 1% low-melt agarose. For larvae imaged at 3 and 4 dpf, the yolk was mechanically removed with fine watchmaker forceps prior to mounting.

Whole-mount immunofluorescence staining was performed as previously described [26]. Larvae were euthanized with 5× tricaine and roughly 20 larvae per condition were transferred to a 1.5 mL tube. Excess media was removed and 1 mL of chilled 4% paraformaldehyde was added. Larvae were incubated in fixative overnight (>16 hours) at 4°C. Samples were then washed 2 × 5 min in PBS. For samples of 3 or 4 dpf larvae, yolk was then mechanically removed using fine watchmaker forceps before dehydrating in 50% methanol/50% PBS solution for 5 min. Larvae were then washed 3× in 100% methanol before incubation in 100% methanol at −20°C for at least 2 hours. Larvae were then rehydrated in serial 50% methanol/50% PBS, 100% PBS washes, and permeabilized at room temperature for at least 20 min with PBS with 0.1% Tween-20, 1% DMSO, and 0.3% Triton X-100. Larvae were then washed and blocked with 4% bovine serum albumin solution for at least 30 min at room temperature before washing and incubating in primary antibody solution for at least 24 hours at 4°C. Primary antibodies in this study were diluted at 1:100 [rabbit anti-PYY (custom antibody generated in [100]), rabbit anti-CCK (custom antibody generated in [99]), rabbit anti-Ghrl (Anaspec, 55529), goat anti-IgG (Jackson ImmunoResearch, 115-005-044)] or 1:500 [chicken anti-GFP (Aves, GFP-1010)]. Larvae were then washed every 15 min for 2 hours before incubation with Alexa Fluor Invitrogen secondary antibodies diluted at 1:250 [488 goat anti-chicken (ThermoFisher, A11039), 647 goat anti-rabbit (ThermoFisher, A21244), 568 goat anti-rabbit (ThermoFisher, A11011)]. Larvae were incubated with secondary antibodies overnight (>16 hours) at 4°C, washed every 15 min for 1 hour, and mounted for imaging.

### *neurod1* cell analysis in *neurog3* mutants

The *neurog3*^*sa211*^ mutant allele was obtained from the Zebrafish Mutation Project (Sanger Institute, UK). Adult heterozygous *neurog3*^*sa211*^ zebrafish were bred naturally in system water, and fertilized eggs were collected, transferred to 100 mm petri dishes containing embryo medium, and allowed to develop at 28.5°C. All steps of the staining were performed on nutator at room temperature except when indicated otherwise. Approximately 30 larvae were fixed in 750 µL of 4% PFA prepared in 1× sweet buffer (8% sucrose, 0.2 M CaCl₂, 0.2 M PO₄ buffer, pH 7.3) for 2 hours. Then, larvae were washed twice in 0.5% Phosphate buffer saline-Triton X-100 (PBSTX) for 15 min, and stored in PBSTX at 4°C. Prior to blocking, larvae were washed 5× in double distilled water for a minimum of 60 min each and then they were incubated for 1 hour in blocking solution [0.5% PBS-Triton X-100, 1% DMSO, 2% bovine serum albumin (Sigma-Aldrich, A3059), 5% normal growth serum (ThermoFisher, PCN5000). Next, larvae were incubated for 16–18 hours in anti-DsRed primary antibody (1:300; Takara Bio, 632496) diluted in blocking solution. Larvae were then washed 3× for a minimum of 3 hours each in 0.3% PBS Triton X-100 before incubating for 16–18 hours in Alexa Fluor 568 secondary antibody (1:1,500; ThermoFisher, A11035) diluted in blocking solution. Finally, larvae were washed 3× for a minimum of 3 hours each in dark and stored at 4°C in 0.3% PBS Triton X-100 before mounting.

Before imaging the immunostained intestines, each larva was tail clipped for genotyping and the body stored in 0.3% PBSTX at 4°C. For extraction of genomic DNA, the fin tissue was incubated in 25 µL of 1× lysis buffer [per 1 mL: 100 µL of 10× PCR buffer (BioLabs NEB, B9004S), 50 µL ∼20 mg/mL of proteinase K (ThermoFisher, 100005393), and 850 µL of nuclease-free water] at 55°C for 50 min, followed by 98°C for 10 min. PCR amplification was performed using 1 µL of genomic DNA, 19 µL master mix containing 10 µL of 2× GoTaq Green Master Mix, 7 µL of nuclease-free water, and 1 µL each of the forward (5′-CAAGATCCTCGTGCGCTTTG-3′) and reverse (5′-CCTGATCCTGGGCGTGATTT-3′) primers. The PCR protocol was as follows: 95°C for 2 min; 34 cycles of 95°C for 30 s, 66°C for 30 s, and 72°C for 60 s; final extension at 72°C for 5 min; hold at 12°C. PCR products were cleaned using ExoSAP-IT following the manufactures protocol (ThermoFisher, 78201.1.ML), then sent for Sanger sequencing. Larvae were classified as homozygous wild type or homozygous mutant based on the absence or presence of the mutant allele (ZMP, Zebrafish Mutation Project, Zebrafish Mutant Resource) [180,181]. Only intestines of homozygous wild-type or homozygous mutant were mounted and imaged.

For quantification of EECs, intestines were dissected with forceps and mounted on glass slides in 0.3% PBSTX or Fluoromount-G (SouthernBiotech, 0100-01) and covered with a coverslip. Confocal z-stacks images were captured using the 3i spinning disk confocal microscope with a 20× objective, operated through Slidebook6 software (Version 6.0.13).

### In situ hybridization chain reaction

Probes for *ghrl* RNA (S4 Table) were designed according to previously published parameters [182]. *cubn* probes were generously shared by Dr. Michel Bagnat. Hairpins and amplification, hybridization, and wash buffers were purchased from Molecular Instruments [183]. Our methods were adapted from previously published procedures in zebrafish [109,182]. 6 dpf larvae were fixed in 4% PFA for 3 hours at room temperature, followed by PBS and ice-cold acetone washes before being incubated in acetone at −20°C for 8 min. Larvae were then washed with PBS and incubated with Molecular Instruments probe hybridization buffer for 30 min rotating at 37°C. Larvae were then incubated in probe solution (8 nM of each probe) for 48 hours rotating at 37°C. To remove excess probes, larvae were washed 4 × 30 min each in wash buffer pre-warmed to 37°C followed by 2 × 10 min washes in 5× SSCT buffer at room temperature. Larvae were then incubated in amplification buffer for 30 min at room temperature while hairpins were prepared. Sixty pmol of each hairpin were heated separately to 95°C for 90 s and then snap cooled in the dark at room temperature for 30 min. Larvae were incubated in hairpin solution rotating in the dark overnight at room temperature. Excess hairpins were removed with 2 × 5 min, 2 × 30 min, and 1 × 5 min washes in SSCT buffer at room temperature. Larvae were then mounted and imaged with spinning disc confocal.

### Image analysis with ImageJ

Images were processed and analyzed in ImageJ [184]. Images were automatedly blinded such that the scorer did not know the condition (e.g., ablated or control) of each image. The start and end of the gut was manually marked using the Cell Counter plugin and *x*, *y* coordinates were extracted. *neurod1*+ cells were automatedly counted from maximum intensity projections using the Analyze Particles function with minimum object size of 10 pixels^2^. The number and location of cells was automatedly extracted in R and locations were zero-ed using corresponding manual gut markers. To account for overlapping cells being grouped into a single object, the median object size was used to assign a weight to each object. Objects twice as large as the median size given a weight of 2, objects three times as large as the median were given a weight of 3, and so on, with a minimum weight of 1. Weights were summed to count the total number of cells in an image.

*neurog3*+ cells were counted with a similar automated approach. A composite of *neurod1* and *neurog3* channels was used to identify objects using the Analyze Particles function and the fluorescence of each channel was extracted for each identified object. Objects with a ratio of *neurog3:neurod1* signal >2 were counted as *neurog3*+ only, objects with a ratio of *neurog3:neurod1* signal <0.5 were counted as *neurod1*+ only, and all objects with a ratio in between 2 and 0.5 were counted as double positive. The same adjustment for overlapping cells described above was also applied.

Because of increased background of various sources causing complications with automated thresholding and counting, images from *gcga, trpa1b,* and *ghrl* reporters along with PYY and CCK stains were manually counted using the Cell Counter plugin. Counts for reporter characterization in Fig 3 were taken for each slice of the captured image while maximum intensity projections were used for ablation image analysis for higher throughput. In all cases, the number and location of each manually counted cell were extracted for further analysis.

In addition to total counts, the location of automatedly or manually counted cells was determined relative to the manually annotated start and end points of the gut. In cases where a single object was given a weight >1 and counted as multiple cells, the x, y coordinates of the original object was used for each. Cells were binned into four evenly divided bins along the length of the intestine for visualization of counts per quarter of the gut in bar plots (see Figs 4, 5, S9, and S11) or into 100 evenly divided bins for visualization of density per percentile of the gut in density plots (see Fig 3T–3W).

### Adult sections

Adult zebrafish 3–4 months post fertilization of either sex expressing *Tg(neurod1:RFP); Tg(ghrl:QF2; cmlc2:GFP); Tg(QUAS:GFP; cryaa:mCherry)* were retrieved from the circulating aquaculture system before morning feeding and transferred to a separate tank to fast before dissection. Several hours later fish were euthanized in 0.090% 2-Phenoxyethanol and dissected to retrieve the intestine. The intestine was fixed in 4% PFA overnight at 4°C then washed 3× the next day with cold 1× PBS. The intestine was mounted in 4% low-melt agarose in cryomolds and cured at 4°C for 1 hour. 200 μm cross sections of the tissue were sectioned via Vibratome (Leica VT1000S) and mounted on slides with Vectashield containing DAPI (Vectashield H-1200). Z-stack images were collected on a Zeiss 780 inverted confocal microscope at either 20× or 40× magnification. Image processing was done in FIJI including maximum intensity projections of z-stacks from each channel.

### Adult Swiss rolls

Adult zebrafish (approximately one year post fertilization) expressing *Tg(neurod1:RFP); Tg(ghrl:QF2; cmlc2:GFP); Tg(QUAS:GFP; cryaa:mCherry)* were retrieved from the recirculating aquaculture system and euthanized with 0.090% 2-Phenoxyethanol. Intestines were dissected and flayed open to discard luminal debris using ice-cold 1× PBS. Once cleaned, a few drops of Carnoy’s fixative was used to stiffen the tissue prior to rolling and immediately washed with ice-cold 1× PBS. Intestine was rolled from the proximal to distal end, pinned through with a dissection pin (Fine Science Tools, 26002-10) to maintain shape, then transferred to 4% PFA overnight at 4°C. After overnight fixation, the tissue was transferred to a 30% sucrose solution with 0.02% sodium azide and stored at 4°C until tissue sank in preparation for cryosectioning. Intestines were then mounted in cryomolds (TissueTek, 4566) with FS 22 Clear (Leica, 3801480), frozen in a dry ice bath, and stored as blocks at −80°C until sectioning. Blocks were allowed to equilibrate to internal cryostat temperature for 1 hour prior to sectioning and 10 µm sections were collected via cryostat (Leica, CM1860). Slides were stored at −80°C until imaging. For imaging, sections were mounted with Vectashield containing DAPI (Vectashield, H-1200) and glass coverslips and imaged on an Andor Dragonfly confocal at 20x with z-stack and stitching.

### Survival assays

*neurod1, neurog3,* or *ghrl*-ablated larvae were generated alongside sibling controls, which were identical to their ablated counterparts but lacked the Cre transgene. Groups of 25–30 larvae were placed in housing tanks and put on a recirculating aquaculture system at 6 dpf and were fed and maintained according to standard husbandry methods described above. The number of fish remaining in each tank was counted at three evenly spaced timepoints (2, 4, and 6 weeks post-fertilization for initial experiments and 4, 8, and 12 weeks for extended experiments for *neurog3* and *ghrl* ablated conditions). Any tank that had >50% death rate at the first measurement was excluded from analysis as occasional tank die off is known to occur sporadically. Specifically, excluded tanks were as follows: one control tank in the 12-week *neurog3* ablation experiment and one control and two ablated tanks in the 12-week *ghrl* ablation experiment. At the last time point of each experiment (6 or 12 weeks), manual standard length measurements of roughly representative fish from each tank were performed.

### Single-cell RNA sequencing analysis

We imported data from previously published adult [49] and larval [48] zebrafish intestinal scRNA-seq datasets into R (version 4.3.1). Using established markers of intestinal secretory cells, we selected just secretory cells from each dataset (S1 Fig) and integrated them using the SCT method in Seurat version 5.0.3 [185]. We further subsetted this dataset for just secretory progenitors and EECs and re-integrated and clustered it to generate the dataset shown in Fig 1B and analyzed in this manuscript. This final dataset included 2069 cells from the adult dataset and 1891 cells from the larval dataset. Multiple resolutions were evaluated and a resolution of 0.4 was ultimately used (S16 Fig). We used monocle3 (version 1.3.4) to perform pseudotime analysis where use_partition was set to TRUE and root_cells were set to cluster 7 [58]. Module scores were calculated using the AddModuleScore() function. The list of genes included in the regional modules score was derived from the enriched genes of the pharynx, anterior enterocyte, anterior LRE, posterior LRE, and cloaca clusters in [109] and are listed in the Supplemental Materials (S10 Fig, S1 Table). The integrated secretory progenitor and EEC dataset is available on Zenodo at at DOI 10.5281/zenodo.17342282.

Of note, the zebrafish *gastrin* gene is currently named either *LOC100536965* or *CR556712.1* in danRer11 and is annotated in Ensembl as a noncoding lncRNA (ENSDARG00000117418) for unknown reasons. However, multiple phylogeny studies have identified the exact genetic coordinates of *LOC100536965/CR556712.1* as zebrafish *gastrin* [186–188] and peptides we detected derived from this gene align with RefSeq XM_021479754.1, which is a coding prediction. We therefore refer to the gene as *gastrin*. When searching for expression of gastrin in our scRNA-seq dataset, we found expression under the gene name *CR556712.1* in the larval cells, but no expression of either gene name in the adult cells. Searching using either gene name in the complete published adult dataset [49] also did not return any results, suggesting the gene is either not annotated in that dataset, annotated with a different name, or else not expressed. Detection of *gastrin*-derived peptides in our adult peptidomics samples (S2 Table) suggests that *gastrin* is indeed expressed in adult EECs and that incomplete gene annotation may contribute to this discrepancy.

Further, what we annotated as *tac4b* is currently named *si:ch211-131k2.2* in danRer11 (Ensembl ID ENSDARG00000096645). One of the listed protein names for its corresponding Uniprot entry (R4GDR9), however, is Protachykinin-1 isoform X. To evaluate if this gene may be a member of the tachykinin family, we used Ensembl and NCBI to identify orthologs of zebrafish tachykinin genes (*tac1, tac3a, tac3b, tac4, si:ch211-131k2.2*) in common carp (*Cyprinus carpio carpio*), catfish (*Ictalurus punctatus*), rainbow trout (*Oncorhynchus mykiss*), spotted gar (*Lepisosteus oculatus*), elephant shark (*Callorhinchus milii*), pig (*Sus scrofa*), mouse (*Mus musculus*), and human (*Homo sapiens*). We then performed a protein alignment and phylogenetic analysis using MEGA 12 to produce the phylogenetic tree shown in S15 Fig. This tree showed two *tac4* paralogs in all the teleost fish species included in our analysis, but only one *tac4* gene in gar. This pattern is commonly seen due to the third round (3R) of whole genome duplication in the teleost lineage (S6A Fig) [189,190]. Altogether, our analysis suggests that *si:ch211-131k2.2* is a *TAC4* ortholog. While prior studies had suggested zebrafish has two *tac4* paralogs [191], gene annotation has remained obscure. Based on this alignment, we provisionally renamed zebrafish *tac4* as *tac4a* and *si:ch211-131k2.2* as *tac4b*.

To test the generalizability of our EEC subtype annotations, we downloaded scRNA-seq data from [56] available on the Broad Institute Single Cell Portal. Using the same markers described above, we identified an EEC cluster and subsetted and visualized it using UMAP dimensionality reduction to identify EEC subtypes (S3 Fig).

### Statistical analysis

For the scRNA-seq analysis, statistical analyses for determination of the cluster-enriched markers were calculated using the FindConservedMarkers() function of the Seurat package in R with a Wilcoxon rank sum test. For all other experiments, statistical analysis was performed using unpaired *t* test, or one-way or two-way analysis of variance (ANOVA) with Tukey’s multiple comparisons test with GraphPad Prism. *P* < 0.05 was defined as statistically significant.

### EEC FACs sorting

All media were made fresh the morning of the experiment except for the 6M guanidine hydrochloride, for which the same solution was used throughout the two-week collection period. For larval samples, *Tg(neurod1:RFP); TgBAC(cldn15la:GFP)* adult breeders were crossed and embryos were collected at roughly 6 hours post-fertilization and maintained in egg water [192] at approximately 1 embryo/mL density. Unfertilized embryos were removed at 1 dpf and larvae were anesthetized and sorted for presence of both reporters at 5 dpf. The GentleMACS disassociator (Miltenyi, 130-093-235) was placed in the 4°C room to come to temperature for the following day’s experiment. At 6 dpf, 75 larvae were euthanized in tricaine and pooled into a single sample with 9 samples being processed concurrently with single positive and non-transgenic controls. Larvae were transferred with minimal media to Gentle MACS C tubes (Miltenyi 130-096-334) containing 2 mL of freshly made disassociation buffer [10 mg/mL cold protease (Sigma, P5380-1G), 10 μM ROCK inhibitor (VWR, S1049), 2.5 mg/mL DNase (Sigma, DN25) in Dulbecco’s PBS]. Samples were processed 5 times with protocol C_01 on GentleMACS disassociator with 10 min of incubation on shaker at 4°C after each round. Ten mL of FACS media [0.1% BSA (Fisher, BP1600-100), 10 μM ROCK inhibitor (VWR, S1049) in HBSS (Sigma, H9394-1L)] was added and the contents were poured through a 30 micrometer strainer (Miltenyi 130-098-458) into a fresh 50 mL conical tube. The strainer was washed with an additional 10 mL of FACS media and samples were spun down at 250*g* for 5 min at 4°C, decanted, and resuspended in 1 mL of FACS media. Samples and controls were then transferred to FACS tubes (Corning, 352052) with 5 μL of 7AAD (Sigma, A9400-1MG) to stain dead cells. Cells were then immediately subjected to FACS at the Duke Cancer Institute Flow Cytometry Shared Resource. *neurod1:RFP; cldn15la:GFP* double positive, 7AAD negative cells from each sample were sorted into a single 1.5 mL LoBind tube (Sigma, Z666505) with 500 μL of 6M guanidine hydrochloride for a total of roughly 30,000 cells. This constituted a single sample for downstream peptidomic analysis. Nontransgenic and single transgenic controls (pools of 50 fish per genotype) were prepared as above and used for gating and compensation. A total of three larval peptidomic samples were collected on separate days, all within 1 week of each other.

For adult samples, procedures were largely similar. Male and female *Tg(neurod1:RFP); TgBAC(cldn15la:GFP)* adults were euthanized at 18 months post fertilization and their intestines were dissected, cut along the longitudinal axis to open the gut, and placed in chilled epithelial buffer with 10 μM ROCK inhibitor (5.6 mM Na_2_HPO_4_, 8 mM KH_2_HPO_4,_ 96.2 mM NaCl, 1.6 mM KCl, 43.4 mM sucrose, 54.9 mM d-Sorbitol, 10 μM ROCK inhibitor). Samples rocked gently at 4°C for 15 min before being transferred to fresh epithelial buffer with ROCK inhibitor. Samples were shaken at roughly 150 shakes per minute to remove mucus and fecal debris. Intestines were then transferred to gentle MACS C tubes with 2 mL of disassociation buffer, as above. Intestines were digested with 3 rounds of protocol C_01 with 10 min of rocking at 4°C after each round. Identical to above, 10 mL of FACS media was then added, and contents were passed through a 30 micrometer strainer into a fresh 50 mL conical tube. The strainer was then washed with an additional 10 mL of FACS media and the contents were spun down, decanted, and resuspended in 1 mL of FACs media for immediate sorting. *neurod1:RFP; cldn15la:GFP* double positive, 7AAD negative cells from each of nine samples run in parallel were sorted into a single 1.5 mL LoBind tube (Sigma Z666505) with 500 μL of 6M guanidine hydrochloride for a total of roughly 50,000 cells. This constituted a single sample for downstream peptidomic analysis. Nontransgenic and single transgenic controls (1 dissected intestine per genotype) were prepared as above and used for gating and compensation. A total of three adult peptidomic samples were collected on separate days, all within 1 week of each other. All samples were stored at −80°C until they were transferred to the Duke Proteomics and Metabolomics Core Facility and processed for downstream peptidomics as described below.

### LC–MS/MS analysis of peptidome samples

Samples sorted into 500 μL of 6M guanidine hydrochloride were subjected to five rounds of 30-s bath sonication (Branson) at 30% power with cooling in between. Samples were then diluted with 500 μL of 0.1% formic acid and centrifuged at 12,000 rpm to remove cellular debris. Lysates were loaded directly onto a 10 mg Oasis HLB SPE cartridge (Waters), washed with 2 × 1 mL of 2% acetonitrile/0.1% formic acid, and eluted with 250 μL of 50% acetonitrile/0.1% formic acid. Eluents were speed-vacuumed to dryness. Samples were then resuspended in 50 μL of 50 mM ammonium bicarbonate with 10 mM dithiolthreitol and heated at 70°C for 30 min, alkylated with 25 mM iodoacetamide for 30 min at room temperature and then spiked with 2 fmol/μL of pre-digested yeast alcohol dehydrogenase (Waters MassPrep standard).

LC/MS/MS was performed using an EvoSep One UPLC coupled to a Thermo Orbitrap Astral high-resolution accurate mass tandem mass spectrometer (Thermo). Briefly, each sample loaded EvoTip was eluted onto a 1.5 µm EvoSep 150 μm ID × 15 cm performance (EvoSep) column using the SPD30 gradient at 55°C. Data collection on the Orbitrap Astral mass spectrometer was performed in a data-dependent acquisition mode of acquisition with a *r* = 120,000 (@ *m*/*z* 200) full MS scan from *m*/*z* 300–2,000 in the OT with a target AGC value of 300% and max accumulation time of 50 ms. Data-dependent MS/MS scans in the Astral were performed on charge states 2–7 from *m*/*z* 110–2,000 at a target AGC value of 200% and max accumulation time of 20 ms. HCD collision energy setting of 30% was used for all MS2 scans. Data were imported into PEAKS studio. The software was set to 10 ppm mass accuracy on MS1 and 0.02 Da on MS2 data with no enzyme rules selected. De novo assisted searching was allowed. The MS/MS data was searched against a custom *Danio rerio* database along with a common contaminant/spiked protein database (bovine albumin, bovine casein, yeast ADH, etc.), and an equal number of reversed-sequence “decoys” for false discovery rate determination. Database search parameters included fixed modification on Cys (carbamidomethyl), variable modification on Met (oxidation), C-terminal amide, and N-terminal acetylation and pyroglutamate. We queried our raw data against a custom database of zebrafish proteins from TrEMBL [193] and Ensembl [97]. To account for genomic polymorphisms within the Ekwill zebrafish strain used here, we supplemented the combined TrEMBL/Ensembl database with protein sequences predicted with customProDB [194] from genetic variants observed in previous RNA sequencing from the same strain [195]. Spectral annotation was set at a maximum 1% peptide false discovery rate based on q-value calculations. Manual verification of spectra of interest were confirmed using targeted extraction/selected ion chromatograms with Skyline (University of Washington, MacCoss Laboratory) [196] including considerations for signal to noise, peak shape, mass errors across isotopologues, and retention time relative to database identifications. The mass spectrometry proteomics data have been deposited to the ProteomeXchange Consortium via the PRIDE partner repository with the dataset identifier PXD058654 [197]. In addition, we have shared screenshots of Skyline data for an example of a manually reviewed peptide in S5C and S5D Fig.

### Ghrelin acylation search

To identify acylated ghrelin peptides, we performed a PEAKS search on a custom database just containing the F1QKX9 ghrelin sequences from Uniprot and selected octanoylation and decanoylation as variable posttranslational modifications in addition to the modifications in our original search. Spectral matches and chromatogram peaks of the identified peptides are included in the supplemental materials (S5A and S5B Fig).

### Zebrafish *secretin* peptide search

As the putative zebrafish *secretin* homolog we identified (*si:zfos-2372e4.1*) did not have a TrEMBL entry, it was not listed in our reference dataset, and, as such, was not identified in our initial annotation of our peptidomics dataset. We first ran a preliminary search of the data with a custom dataset that just contained the protein sequences available on Ensembl for *si:zfos-2372e4.1.* We saw several hits, indicating peptides from that gene were indeed present in our sample. As searching with a drastically smaller reference dataset can inflate the possibility of identifying significant peptides, we added those two *si:zfos-2372e4.1* protein entries to our pre-existing custom zebrafish reference dataset, and re-ran the larger search. In our final annotation (Fig 2F), we only included those peptides that met significance in this more stringent second search.

### Protein alignments

Protein sequences for *Danio rerio*, *Carassius auratus*, *Ictalurus punctatus*, *Salmo salar*, *Oncorhynchus mykiss*, Aquarana catesbeiana, *Gallus gallus*, *Rattus norvegicus*, *Mus musculus*, *Homo sapiens*, *Cyprinus carpio carpio*, *Tetraodon nigroviridis*, *Xenopus tropicalis*, *Gasterosteus aculeatus aculeatus*, *Dicentrarchus labrax*, *Myripristis murdjan*, *Clupea harengus*, *Rana temporaria*, *Oreochromis niloticus* were downloaded from UniProt [193]. When more than one sequence was available, the sequence designated as canonical in UniProt was used. If there were multiple relevant entries, a representative one was used for ease of visualization. Sequences were aligned using MUSCLE [198] and visualized with JalView [199]. Percent identity settings were used to color residues.

For tachykinin analysis (S15 Fig), Molecular Evolutionary Genetics Analysis (MEGA) version 12 [200] was used to align protein sequences (method: MUSCLE [198]) and generate a phylogenetic tree shown in S15 Fig (method: Neighbor-Joining [201] with bootstrapping).

## Supporting information

S1 Data Underlying data for quantitative panels. (XLSX)

S1 Table EEC scRNA-seq markers. (XLSX)

S2 Table Zebrafish EEC peptidomics. (XLSX)

S3 Table *SECRETIN* synteny genome coordinates. (XLSX)

S4 Table Reagent sequences. (XLSX)

S1 Fig Creation of joint larval and adult intestinal secretory cell scRNA-seq dataset. **(A)** scRNA-seq data of the adult zebrafish intestine from [49] was clustered and evaluated for expression of secretory cell markers. Clusters showing strong expression of these markers and intestinal epithelial markers but minimal pancreatic endocrine cell markers were selected for subsequent analysis and are outlined with red boxes. **(B)** We similarly processed scRNA-seq data of the larval zebrafish intestine from [48] and selected clusters outlined in red for subsequent analysis. **(C)** UMAP of the joint adult and larval dataset generated by integrating and re-clustering cells identified in panels A and B. Clusters circled with a dashed line were identified as EECs and secretory progenitors based on expression shown in **(D)** and were selected for subsequent integration and re-clustering to form the dataset shown in Fig 1.
(TIF)

S2 Fig Additional characterization of joint adult and larval secretory progenitor and EEC scRNA-seq dataset. **(A)** Expression dotplot showing secretory progenitor markers are enriched in clusters 7–10. **(B)** UMAP colored by dataset of origin for each cell with adult cells in green and larval cells in blue. **(C)** Stacked bar plot showing the percentage of cells in each EEC cluster from the adult, larval, or combined dataset. **(D)** Heatmap showing the z-scored expression of the 10 most highly enriched markers for each EEC cluster. **(E)** Heatmap showing the z-scored expression of genes annotated as receptors by [202] that were significantly enriched in both larval and adult cells in each cluster. **(F)** Heatmap showing the z-scored expression of genes annotated as ligands by [202] that were significantly enriched in both larval and adult cells in each cluster. **(G)** Table of major differences between the samples that generated the larval and adult scRNA-seq datasets. Underlying data can be found in S1 Data.
(TIF)

S3 Fig Clustering of EEC subtypes in additional scRNA-seq dataset. **(A)** scRNA-seq data of the larval zebrafish digestive tract from [56] was clustered and evaluated for expression of enteroendocrine cell (EEC) markers. The cluster showing strong expression of these markers and intestinal epithelial markers, but minimal pancreatic endocrine cell markers was selected for subsequent analysis and is outlined with a red box on the dotplot and red circle on the UMAP. **(B)** Those selected EECs were then re-clustered to identify EEC subtypes by examining EEC marker expression. **(C)** Seurat Feature Plot with simultaneous visualization of *ghrl* (red) and *pyyb* (blue) expression using the blend function, where overlapping expression would be shown in pink according to the relative expression scale shown. These plots show that, while both genes are expressed in the same cluster in this analysis, they label distinct populations of cells within that cluster. Underlying data can be found in S1 Data.
(TIF)

S4 Fig Zebrafish EEC peptide atlas. In all panels, the primary amino acid sequence of the gene of interest is shown at the top in black with any identified missense variants indicated in red above. Blue horizontal lines below the amino acid sequence represent the unique peptides detected in our study with small black vertical lines denoting the stop and start of each peptide. Abutting peptides share a single vertical line to represent the stop of the preceding peptide and the start of the subsequent one. Different colored squares represent various posttranslational modification detected. Shading labels regions aligning to Uniprot-annotated peptides in humans. In cases where multiple peptides are known to be processed from the same sequence, dashed lines indicate different cleavage sites. Below the detected peptides is a multispecies protein alignment where amino acids are color-coded based on their percent identity match across all the reported species with darker coloring indicating a more conserved residue. Human processed peptide annotations taken from Uniprot are labeled below with horizontal lines. The color of these lines corresponds to the color of the shading of the aligned zebrafish amino acids above. Note that many of the peptide cleavage sites occur at dibasic residues (i.e., RR/KR/KK), consistent with cleavage by prohormone convertase enzymes [148]. More detailed information about the peptides shown in this figure are available in S2 Table. **(A)** Zebrafish *adcyap1a-*derived peptides detected in EECs. Alignment of the primary amino acid sequence of Pituitary adenylate cyclase-activating polypeptide protein includes zebrafish (Uniprot Q98TU3), trout (Uniprot A0A8C7QBD4), goldfish (Uniprot A0A6P6RC36), catfish (Uniprot Q90XZ4), chicken (Uniprot Q58FG9), mouse (Uniprot O70176), rat (Uniprot A6KFB2), human (Uniprot P18509). **(B)** Zebrafish *gcga-* and *gcgb-*derived peptides detected in EECs. As *gcga* and *gcgb* are both orthologs of human *GCG*, they are reported together. Peptides aligning to multiple isoforms of *gcga* were detected. Alignment of the primary amino acid sequence of Pro-glucagon protein includes zebrafish *gcga* isoform 2 (Uniprot F1RD10), zebrafish *gcga* isoform 1 (Uniprot A0A0R4IS85), seabass *gcga* (Uniprot A0A8C4IA77), soldierfish *gcga* (Uniprot A0A667XLC6), herring *gcga* (Uniprot A0A6P3WDK7), zebrafish *gcgb* (Uniprot B0R1C3), seabass *gcgb* (Uniprot A0A8P4K489), soldierfish *gcgb* (Uniprot A0A668AYA2), herring *gcgb* (Uniprot A0A6P3W7W6), mouse *Gcg* (Uniprot A2AS86), rat *Gcg* (Uniprot A6HLV7), human (Uniprot P01275). Of note, the arginine 36 residue in GLP-1 is commonly amidated [144,145], as seen in the *gcgb*-derived peptide reported here. **(C)** Zebrafish *calca*-derived peptides detected in EECs. Alignment of the primary amino acid sequence of Calcitonin protein includes zebrafish (Uniprot F1QIK6), catfish (Uniprot A0A2D0RV9), goldfish (Uniprot A0A6P6MFE7), salmon (Uniprot A0A1S3KMJ8), chicken (Uniprot P07660), mouse (Uniprot P70160), rat (Uniprot P01257), and human (Uniprot P01258). **(D)** Zebrafish *ccka-* and *gast-*derived peptides detected in EECs. As Cholecystokinin (CCK) and Gastrin are closely related, they were aligned together, but are displayed separately for ease of visualization. Of note, the zebrafish *gastrin* gene is currently named as *CR556712.1* or *LOC100536965*, but due to evidence from published phylogenetic studies [186–188] and protein sequence alignment, we refer to it as *gastrin* (see Methods). Alignment of the primary amino acid sequence of CCK and Gastrin includes zebrafish *ccka* (Uniprot E9QEB3), carp *ccka* (Uniprot A0A9R1SKH2), tetraodon *ccka* (Uniprot Q8AXP6), xenopus *cck* (Uniprot A0A803J912), chicken *cck* (Uniprot Q9PU41), mouse *Cck* (Uniprot P09240), human *CCK* (Uniprot P06307), zebrafish *gast* (Uniprot A0A8M6Z182), carp *gast* (Uniprot A0A8C1B8C9), tetraodon *gast* (Uniprot Q8AXP5), stickleback *gast* (Ensembl ENSGACT00000024275.2), xenopus *gast* (Uniprot F6W277), chicken *gast* (Ensembl ENSGALT00000043859.2), mouse *Gast* (Uniprot P48757), human *GAST* (Uniprot P01350). **(E)** Zebrafish *galn*-derived peptides detected in EECs. Alignment of the primary amino acid sequence of Galanin includes zebrafish (Uniprot E7EZ53), goldfish (Uniprot Q7ZT91), catfish (Uniprot A0A2D0RH49), salmon (Uniprot A0A1S3KNE4), trout (Uniprot A0A060WZ82), chicken (Uniprot A0A8V0ZZ93), mouse (Uniprot P47212), rat (Uniprot A6HYK9), human (Uniprot P22466). **(F)** Zebrafish *gip*-derived peptides detected in EECs. Alignment of the primary amino acid sequence of Gastric inhibitory polypeptide includes zebrafish (Uniprot A1DPK4), trout (Uniprot A0A060W6J5), salmon (Uniprot A0A1S3NHD2), chicken (Uniprot A0A8V1AAS2), bullfrog (Uniprot A0A2G9RZ64), mouse (Uniprot P48756), rat (Uniprot A6HIC4), human (Uniprot P09681). **(G)** Zebrafish *insl5a-* and *insl5b-*derived peptides detected in EECs. As *insl5a* and *insl5b* are both orthologs of human *INSL5*, they are reported together. Alignment of the primary amino acid sequence of Insulin-like peptide 5 includes zebrafish *insl5a* (Uniprot Q2VT44), goldfish *insl5a* (Uniprot 0A6P6KC48), carp *insl5a* (Uniprot A0A8C1HD46), zebrafish *insl5b* (Uniprot A0ZYT5), goldfish *insl5b* (Uniprot A0A6P6JU13), carp *insl5b* (Uniprot A0A9J8CMM8), xenopus *insl5* (Uniprot A0A6I8SQK1), mouse *Insl5* (Uniprot Q9WUG6), human *INSL5* (Uniprot Q9Y5Q6). **(H)** Zebrafish *mlnl*-derived peptides detected in EECs. Alignment of the primary amino acid sequence of Promotilin includes zebrafish (Uniprot E9QFU1), trout (Uniprot A0A060WHA4), chicken (Uniprot A0A8V1AED3), rat (Uniprot A8IRI0), human (Uniprot P12872). **(I)** Zebrafish *nmbb*-derived peptides detected in EECs. Alignment of the primary amino acid sequence of Neuromedin B includes zebrafish (Uniprot B3DFU2), goldfish (Uniprot A0A6P6PMY7), trout (Uniprot A0A8K9XPV7), chicken (Uniprot A0A8V0ZQC9), mouse (Uniprot Q9CR53), rat (Uniprot A6JCD9), human (Uniprot P08949). **(J)** Zebrafish *pdyn*-derived peptides detected in EECs. Alignment of the primary amino acid sequence of Proenkephalin B includes zebrafish (Uniprot Q6JT77), goldfish (Uniprot A0A6P6NSK2), catfish (Uniprot E3TFZ0), salmon (Uniprot B5X928), trout (Uniprot A0A060WJX8), chicken (Uniprot A0A8E7KML8), mouse (Uniprot O35852), rat (Uniprot F1M7S3), human (Uniprot P01213). **(K)** Zebrafish *penka*-derived peptides detected in EECs. Alignment of the primary amino acid sequence of Proenkephalin A includes zebrafish (Uniprot A8E7S2), goldfish (Uniprot A0A6P6MMG9), trout (Uniprot A0A8C7NZE4), salmon (Uniprot B5X739), chicken (Uniprot E1C652), mouse (Uniprot P22005), rat (Uniprot A6JFM8), human (Uniprot P01210). **(L)** Zebrafish *pyyb*-derived peptides detected in EECs. Alignment of the primary amino acid sequence of Peptide YY (PYY) includes zebrafish (Uniprot E7F0L6), goldfish (Uniprot A0A6P6PFJ3), catfish (Uniprot W5UQI6), trout (Uniprot A0A060XNM3), salmon (Uniprot A0A1S3S6S3), chicken (Uniprot A0A0S3UPI6), bullfrog (Uniprot A0A2G9P7P3), mouse (Uniprot H3BK86), rat (Uniprot F1LSR6), human (Uniprot P10082). **(M)** Zebrafish *sst2-* and *sst1.2*-derived peptides detected in EECs. As both *sst2* and *sst1.2* are orthologs of human *SST*, they are reported together. *sst2* is considered to be part of SST family 4 and *sst1.2* is considered to be part of SST family 3 [203]. Alignment of the primary amino acid sequence of Somatostatin includes zebrafish *sst2* (Uniprot Q9DDE4), goldfish *sst2* (Uniprot A0A6P6P309), carp *sst2* (Uniprot A0A8C1E251), zebrafish *sst1.2* (Uniprot E7FFY9), carp *sst1.2* (Uniprot A0A8C1BWP9), catfish *sst1.2* (Uniprot W5UDS0), frog *sst* (RefSeq XP_040205526.1), chicken *sst* (Uniprot P33094), mouse *Sst* (Uniprot P60041), human *SST* (Uniprot P61278). **(N)** Zebrafish *tac3a*-derived peptides detected in EECs. Alignment of the primary amino acid sequence of Tachykinin 3 includes zebrafish (Uniprot E9QE01), goldfish (Uniprot T2CZA2), catfish (Uniprot I4IY93), trout (Uniprot A0A8C7RBE8), salmon (Uniprot I4IY94), mouse (Uniprot P55099), rat (Uniprot A6HQX0), human (Uniprot Q9UHF0). **(O)** Zebrafish *vipb-*derived peptides detected in EECs. Peptides aligning to multiple isoforms of *vipb* were detected. Alignment of the primary amino acid sequence of Vasoactive intestinal peptides protein include zebrafish isoform 1 (Uniprot A0A8M9Q6Y9), zebrafish isoform 2 (Uniprot B0LF71), goldfish (Uniprot A0A6P6NBR0), catfish (Uniprot A0A2D0RS13), salmon (Uniprot A0A1S3PEV), trout (Uniprot A0A060X5U6), chicken (Uniprot P48143), mouse (Uniprot A0A0R4J003), rat (Uniprot A0A090AX27), human (Uniprot P01282). **(P)** Zebrafish *ins*-derived peptides detected in EECs. Alignment of the primary amino acid sequences of Insulin protein include zebrafish (Uniprot B2GSI0), stickleback (Ensembl ENSGACT00000066960.1), tilapia (Uniprot I3IUZ1), herring (Ensembl ENSCHAT00020053570.1), xenopus (Uniprot F6QRS1), chicken (Uniprot P67970), mouse (Uniprot P01326), rat (Uniprot P01323), human (Uniprot P01308). **(Q)** Zebrafish *pcsk1nl*-derived peptides detected in EECs. **(R)** Zebrafish *pcsk1*-derived peptides detected in EECs. **(S)** Zebrafish *pcsk2*-derived peptides detected in EECs. **(T)** Zebrafish *scg2a*-derived peptides detected in EECs. **(U)** Zebrafish *scg2b*-derived peptides detected in EECs. **(V)** Zebrafish *scg3-*derived peptides detected in EECs. **(W)** Zebrafish *scg5*-derived peptides detected in EECs. **(X)** Zebrafish *scgn*-derived peptides detected in EECs.
(PDF)

S5 Fig Example raw peptidomic data of acylated ghrelin and manually curated peptides. **(A)** Fragmentation spectrum identifying octanolylated ghrelin peptide. **(B)** Fragmentation spectrum identifying decanolylated ghrelin peptide. **(C)** Chromatogram data showing overlapping peaks for double, triple, and quadruple charged YGGFLRKFGPK peptide manually identified from the Pdyn protein. **(D)** Chromatogram data showing overlapping peaks for isotopologues M, M + 1, and M + 2 of the +3 charged peptide. Peak areas are quantified below and mass error for each is shown. **(E)** Expression in the joint larval and adult EEC dataset of hormone genes commonly enriched in the pancreatic islet. Underlying data can be found in S1 Data.
(TIF)

S6 Fig Synteny of *SECRETIN* loci across vertebrates. **(A)** Two rounds of whole genome duplication (labeled 1R and 2R) preceded the divergence of vertebrates [204–207]. The most ancient split within vertebrates is between the jawless Cyclostomi, such as lamprey, and the remaining Gnathostomata. The jawed vertebrates can be divided into those with primarily cartilaginous (Chondrichthyes) or bony (Osteichthyes) skeletons. A final round of whole genome duplication (labeled 3R) occurred in teleost fishes [189,190]. **(B)** The arrangement of genes along human chromosome 11, elephant shark scaffold 17, gar Chromosome 21, and zebrafish chromosome 25 are shown with genes ordered by ascending start location. Genes were identified by searching the ~250,000 base pairs upstream and downstream of human *SCT* and cross-checking with orthologs in zebrafish. Any gap equal to or greater than 0.5 Mega bases is annotated. The location of *SCT* and its direct neighbors are traced between each species with lines. Exact location of each gene is shown in S3 Table.
(TIF)

S7 Fig Creation of *ghrl:QF2* and *neurog3:QF2* lines. **(A)** Schematic representation of cloning approach to generating *neurog3:QF2* and *ghrl:QF2* reporters. **(B)** mVISTA [178,179] alignment of the *ghrl* locus in six closely related *Danio* species. *Danio rerio ghrl* annotation is shown at the top and is used as the reference. Dashed lines mark the highly conserved 647 base pair region upstream of the *ghrl* transcriptional start site that was cloned for the *ghrl:QF2* reporter. **(C)** Schematic of where representative cross sections were taken to evaluate the *ghrl:QF2* reporter in adult intestines in panels E and F. **(D)** Schematic of Swiss roll preparation and sectioning of full-length intestine from *ghrl:QF2* reporter adults in panel G. **(E)** Representative image of a proximal intestinal section showing *ghrl*+; *neurod1*+ cells with classical EEC morphology. **(F)** Representative image of *ghrl*+; *neurod1+* cell with classical EEC morphology in a distal intestinal section. **(G)** Section of Swiss-rolled adult *ghrl:QF2* reporter intestine where the proximal gut is closest to the center. Several *ghrl*+; *neurod1*+ cells are highlighted. **(H)** Quantification of *ghrl*+ cells overlap with the pan-EEC reporter *neurod1:RFP* across larval development. Each dot represents an individual fish. **(I)** A representative image of a 5 dpf *ghrl:QF2* fish with a highlighted example of a *ghrl*+; *neurod1-* cell. **(J)** Staining of 6 dpf larvae with anti-Ghrelin primary antibody and appropriate secondary antibody co-labels cells with *ghrl:QF2* reporter in the pancreatic islet and intestine. While staining is not very strong, it is clearly stronger than negative controls run with **(K)** non-specific anti-IgG primary antibody and appropriate secondary antibody or **(L)** anti-Ghrelin primary antibody and no secondary antibody. **(M)** *ghrl:QF2* reporter co-labels with hybridization chain reaction (HCR) probes targeting *ghrl* mRNA in the islet and intestine. HCR probes targeting *cubn,* a gene enriched in the lysosome-rich enterocytes (LREs) in the distal intestine, serve as a positive control for the HCR technique. **(N)** The *ghrl:QF2* reporter and *sst2:DsRed* reporter both label EECs in the intestine. While they overlap in many cells, some cells are only *ghrl* reporter+ and some are only *sst2* reporter+, particularly in the distal intestine. **(O)** UMAP of secretory progenitors and EECs from larval and adult zebrafish shown in Fig 1. **(P)** Seurat Feature Plot with simultaneous visualization of *ghrl* (red) and *sst2* (blue) expression using the blend function, where overlapping expression is shown in pink according to the relative expression scale shown. **(Q)** Seurat Feature Plot of only *ghrl* expression, derived from the blend-plot described in P. **(R)** Seurat Feature Plot of only *sst2* expression, derived from the blend-plot described in P. **(S)** A representative image of a 6 dpf *neurog3:QF2* reporter fish with a highlighted example of both a *neurog3*+; *neurod1-* and *neurog3*+; *neurod1+  *cell. **(T)** Quantification of *neurog3*:*QF2* reporter overlap with *neurod1:RFP* reporter across larval development. Each dot represents an individual fish. All scale bars are 100 μm. Underlying data can be found in S1 Data.
(TIF)

S8 Fig EEC subtype reporter imaging across larval development. Representative images showing subtype distribution and overlap with pan-EEC *neurod1* reporter at 3, 4, and 5 dpf for **(A)** *neurog3* reporter, **(B)** *ghrl* reporter, **(C)** anti-PYY antibody, **(D)** *gcga* reporter, **(E)** anti-CCK antibody, **(F)** *trpa1b* reporter. Scale bars are 100 μm.
(TIF)

S9 Fig EEC subtype reporter cell counts across larval development. The percent of *neurod1*+ cells that label with the subtype reporter and the raw counts of subtype numbers per each equal-length quarter of the gut is shown for 3, 4, 5, and 6 days post fertilization (dpf) fish in the **(A)** *neurog3* reporter, **(B)** *ghrl* reporter, **(C)** anti-PYY antibody, **(D)** *gcga* reporter, **(E)** anti-CCK antibody, and **(F)** *trpa1b* reporter. Each dot represents a fish. Underlying data can be found in S1 Data.
(TIF)

S10 Fig Regional patterns in EEC subtypes. **(A)** Cartoon of larval zebrafish intestine. Region-specific genes along the intestinal epithelium were determined using the scRNA-seq dataset from [109]. Horizontal bars below the cartoon represent the approximated anteroposterior location of a given regional cell type and are marked with the panel letter that examines expression of genes specific to that region. Module scores in our combined larval and adult scRNA-seq dataset of the expression of the 25 most enriched genes in the **(B)** pharynx, **(C)** anterior enterocytes, **(D)** ileocytes, **(E)** anterior lysosome-rich enterocytes (LREs), **(F)** posterior LREs, and **(G)** cloaca are shown. Underlying data can be found in S1 Data.
(TIF)

S11 Fig Ablation validation and alterations in regional distribution. **(A)** Schematic of *neurog3*+ ablation combined with *neurog3*+, *neurod1*+ cell labeling to determine *neurog3*+ ablation efficiency. **(B)** Total and regional counts of *neurog3*+ cells in *neurog3+* ablation. **(C)** Total and regional counts of *neurod1*+ cells in control, *neurog3*+, and *neurod1*+ ablation. **(D)** Total and regional counts of *trpa1b*+ cells in control, *neurog3*+, and *neurod1*+ ablation. **(E)** Total EEC and subtype counts in control and *neurog3*+ ablated fish when qrt4 cells are excluded. Counts that include qrt4 are shown in Fig 4C–4H. **(F)** Total EEC and subtype counts in control and *ghrl*-ablated fish when qrt4 cells are excluded. Counts that include qrt4 are shown in Fig 5B–5F. Underlying data can be found in S1 Data.
(TIF)

S12 Fig *neurog3:Cre* induced ablation leads to similar loss of *neurod1*+ cells as *neurog3:QF2; QUAS:Cre.* **(A)** Schematic of *neurog3:Cre* induced *neurog3+* cell ablation. **(B)** Total counts and regional counts of *neurod1*+  cells in the *neurog3:Cre*-ablated and control fish. Each dot represents a 6-day post-fertilization fish. Statistical significance was calculated by unpaired *t* test for total cell numbers and by two-way ANOVA for regional analysis. Significance annotations are as follows: ns (*p* > 0.05), * (*p* < 0.05), ** (*p* < 0.01), *** (*p* < 0.001), **** (*p* < 0.0001). Underlying data can be found in S1 Data.
(TIF)

S13 Fig Loss of *ghrl* or *neurog3* does not impact *neurod1*+ EEC numbers. **(A)** Schematic of the *ghrl* locus with arrowheads marking the sites targeted with CRISPR guide RNAs. **(B)** Genotyping and control gels run with 1 kb+ NEB ladder and 3 *ghrl* mutant and 3 *ghrl* wildtype samples. Amplification with genotyping primers (red arrows) across the 804 base pair region results in a 300 base pair band due to deletion in mutants (lanes 1–3). Wildtype samples (lanes 4–6) do not amplify due to the highly repetitive nature of intron 2, but all samples amplified with control primers amplifying at a non-affected locus (*mttp* gene). **(C)** Total counts and regional counts of *neurod1+* cells in *ghrl* wildtype (heterozygous) and *ghrl* mutant (homozygous) fish. **(D)** Total counts of *neurod1*+ cells in *neurog3* wildtype (homozygous) and *neurog3* mutant (homozygous) fish. Each dot represents a 5–6-day post-fertilization fish. Statistical significance was calculated by unpaired *t* test for total cell numbers and by two-way ANOVA for regional analysis. Underlying data can be found in S1 Data.
(TIF)

S14 Fig Impact of ablation on adult growth and survival. **(A)** Schematic of adult experiments demonstrating when survival counts and standard length measurements were taken. **(B)** Schematic of standard length measurements performed at the endpoint of each experiment. **(C)** *neurod1*-ablated versus sibling control survival and growth data from two independent experiments, each carried out over 6 weeks. Survival curves are significantly different by Mantel-Cox log-rank test (*P* = 0.0006). **(D)** *neurog3*-ablated versus sibling controls followed for 6 weeks showed no significant difference in survival or growth. **(E)** *neurog3*-ablated versus sibling controls followed for 12 weeks again showed no significant differences in survival or growth. **(F)** *ghrl*-ablated versus sibling controls followed for 6 weeks showed no significant difference in survival or growth. **(G)** *ghrl*-ablated versus sibling controls followed for 12 weeks again showed no significant differences in survival or growth. Survival analyses were performed on the pooled results across the number of tanks specified in each panel. Statistical significance was calculated by Mantel-Cox log-rank test for survival curves and by unpaired *t* test for standard length. Underlying data can be found in S1 Data.
(TIF)

S15 Fig Phylogeny of tachykinin family. The analytical procedure encompassed 33 amino acid sequences of tachykinin proteins identified from zebrafish (*Danio* rerio), common carp (*Cyprinus carpio carpio*), catfish (*Ictalurus punctatus*), rainbow trout (*Oncorhynchus mykiss*), spotted gar (*Lepisosteus oculatus*), elephant shark (*Callorhinchus milii*), pig (*Sus scrofa*), mouse (*Mus musculus*), and human (*Homo sapiens*). The gene name and accession number are shown for each branch. Proposed tachykinin family names are shown for those genes without one. The evolutionary history was inferred using the Neighbor-Joining method [201]. The optimal tree with the sum of branch length = 11.694 is shown. The percentage of replicate trees in which the associated taxa clustered together in the bootstrap test (100 replicates) are shown next to the branches [208]. The tree is drawn to scale, with branch lengths in the same units as those of the evolutionary distances used to infer the phylogenetic tree. The evolutionary distances were computed using the Poisson correction method [209] and are in the units of the number of amino acid substitutions per site. The pairwise deletion option was applied to all ambiguous positions for each sequence pair resulting in a final data set comprising 226 positions. Evolutionary analyses were conducted in MEGA12 [200,210] utilizing up to 4 parallel computing threads.
(TIF)

S16 Fig Resolution of combined larval and adult scRNA-seq dataset. Clustering for the scRNA-seq dataset presented in Fig 1 is shown here at resolution 0.1–0.8. The resolution of 0.4 was used for this study.
(TIF)

S1 Raw Images Raw images of gels used in publication. (TIF)

## Abbreviations

ANOVA: analysis of variance
DTA: diphtheria toxin subunit A
EECs: enteroendocrine cells
gRNAs: guide RNAs
INSL5,: insulin-like 5;
PBSTX: phosphate buffer saline-Triton X-100
scRNA-seq: single-cell RNA sequencing
