# Supplementary figures and images for: Identification of intestinal enteroendocrine cell subtypes and their associated hormones in zebrafish
Source: PLoS Biol. 2025 Dec 18;23(12):e3003522. doi: 10.1371/journal.pbio.3003522 (PMC12714231; doi:10.1371/journal.pbio.3003522)

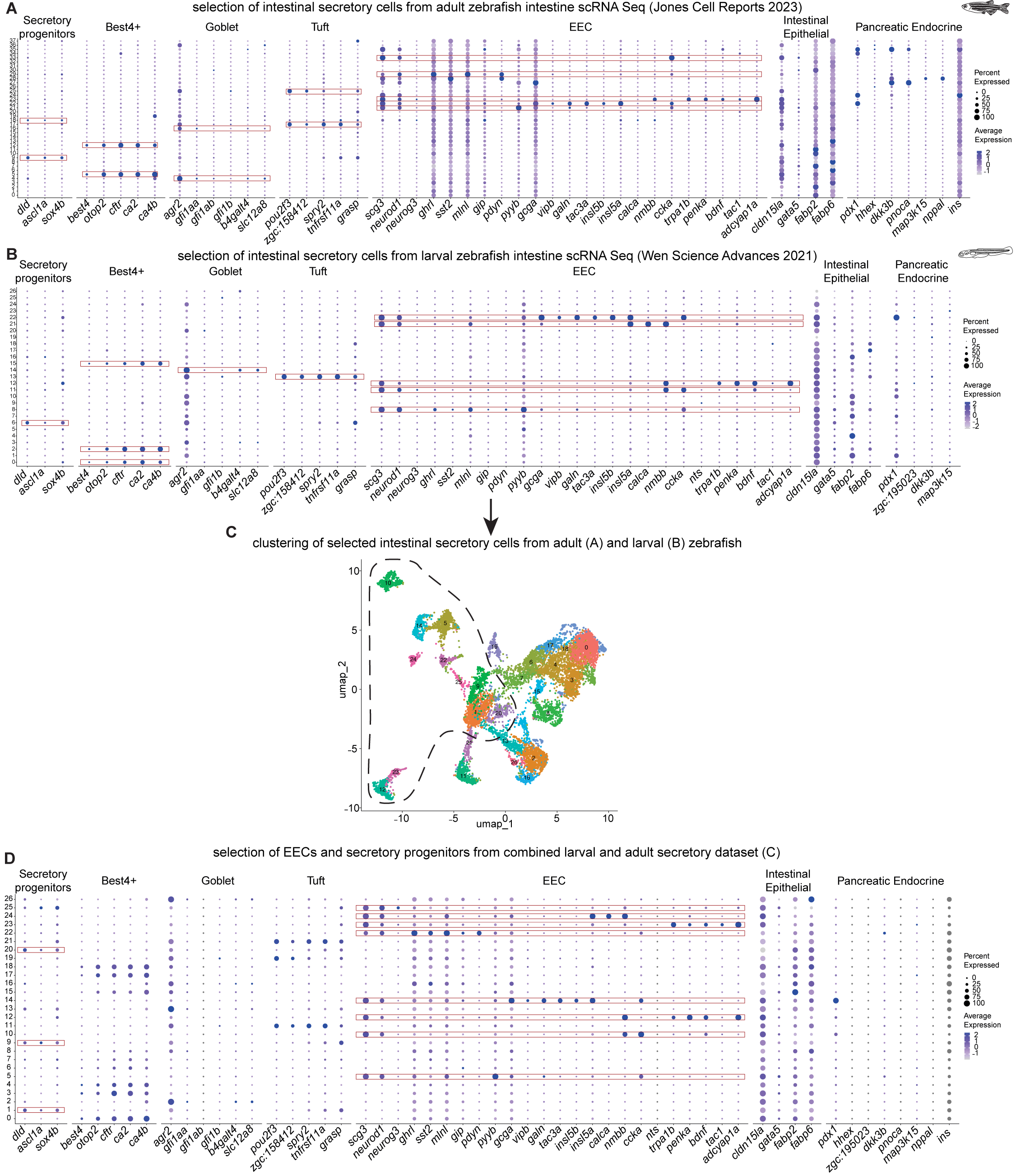

Supplement: S1 Fig — (A) scRNA-seq data of the adult zebrafish intestine from [49] was clustered and evaluated for expression of secretory cell markers. Clusters showing strong expression of these markers and intestinal epithelial markers but minimal pancreatic endocrine cell markers were selected for subsequent analysis and are outlined with red boxes. (B) We similarly processed scRNA-seq data of the larval zebrafish intestine from [48] and selected clusters outlined in red for subsequent analysis. (C) UMAP of the joint adult and larval dataset generated by integrating and re-clustering cells identified in panels A and B. Clusters circled with a dashed line were identified as EECs and secretory progenitors based on expression shown in (D) and were selected for subsequent integration and re-clustering to form the dataset shown in Fig 1. (TIF) [file pbio.3003522.s006.tif]

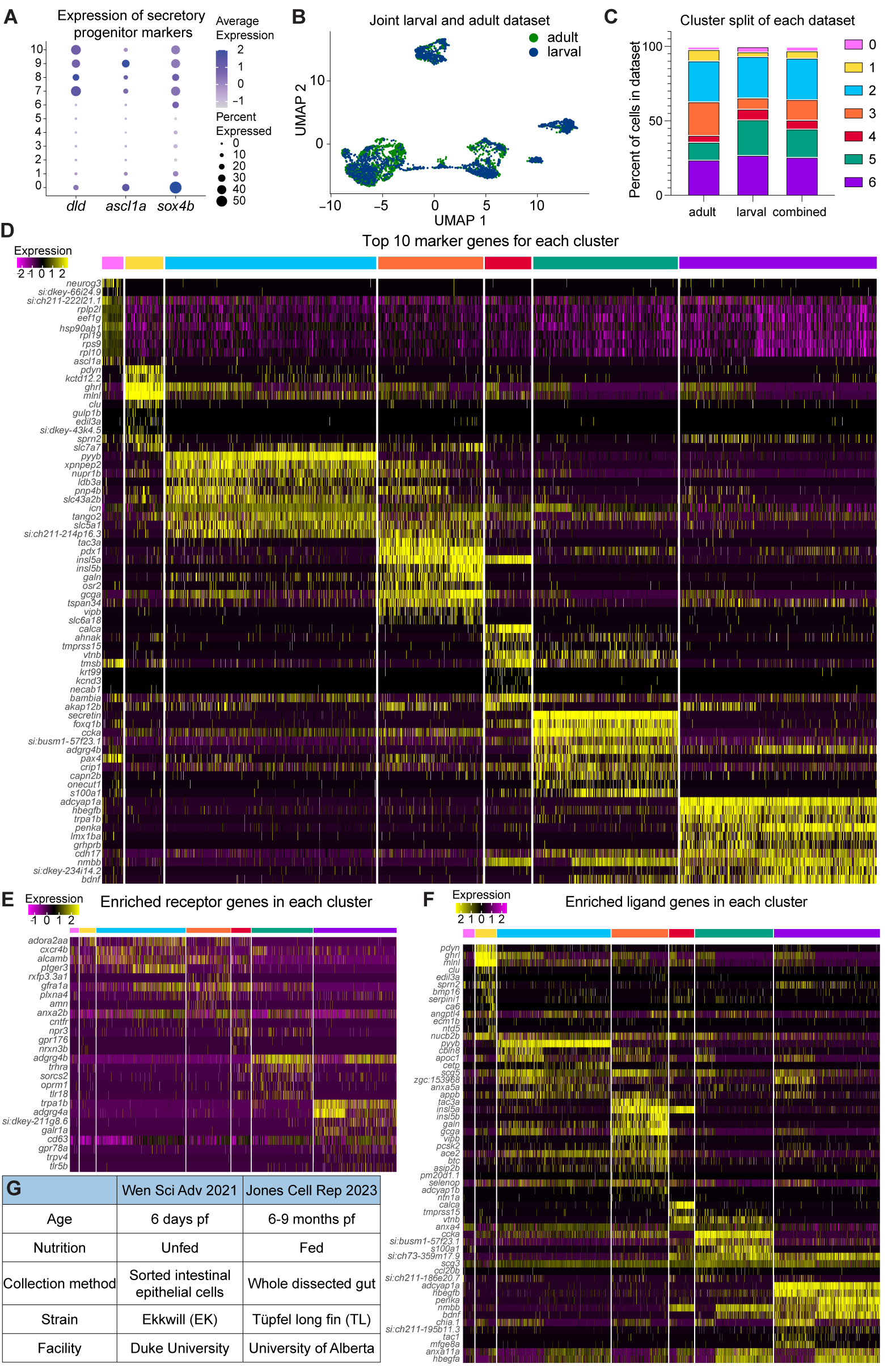

Supplement: S2 Fig — (A) Expression dotplot showing secretory progenitor markers are enriched in clusters 7–10. (B) UMAP colored by dataset of origin for each cell with adult cells in green and larval cells in blue. (C) Stacked bar plot showing the percentage of cells in each EEC cluster from the adult, larval, or combined dataset. (D) Heatmap showing the z-scored expression of the 10 most highly enriched markers for each EEC cluster. (E) Heatmap showing the z-scored expression of genes annotated as receptors by [202] that were significantly enriched in both larval and adult cells in each cluster. (F) Heatmap showing the z-scored expression of genes annotated as ligands by [202] that were significantly enriched in both larval and adult cells in each cluster. (G) Table of major differences between the samples that generated the larval and adult scRNA-seq datasets. Underlying data can be found in S1 Data. (TIF) [file pbio.3003522.s007.tif]

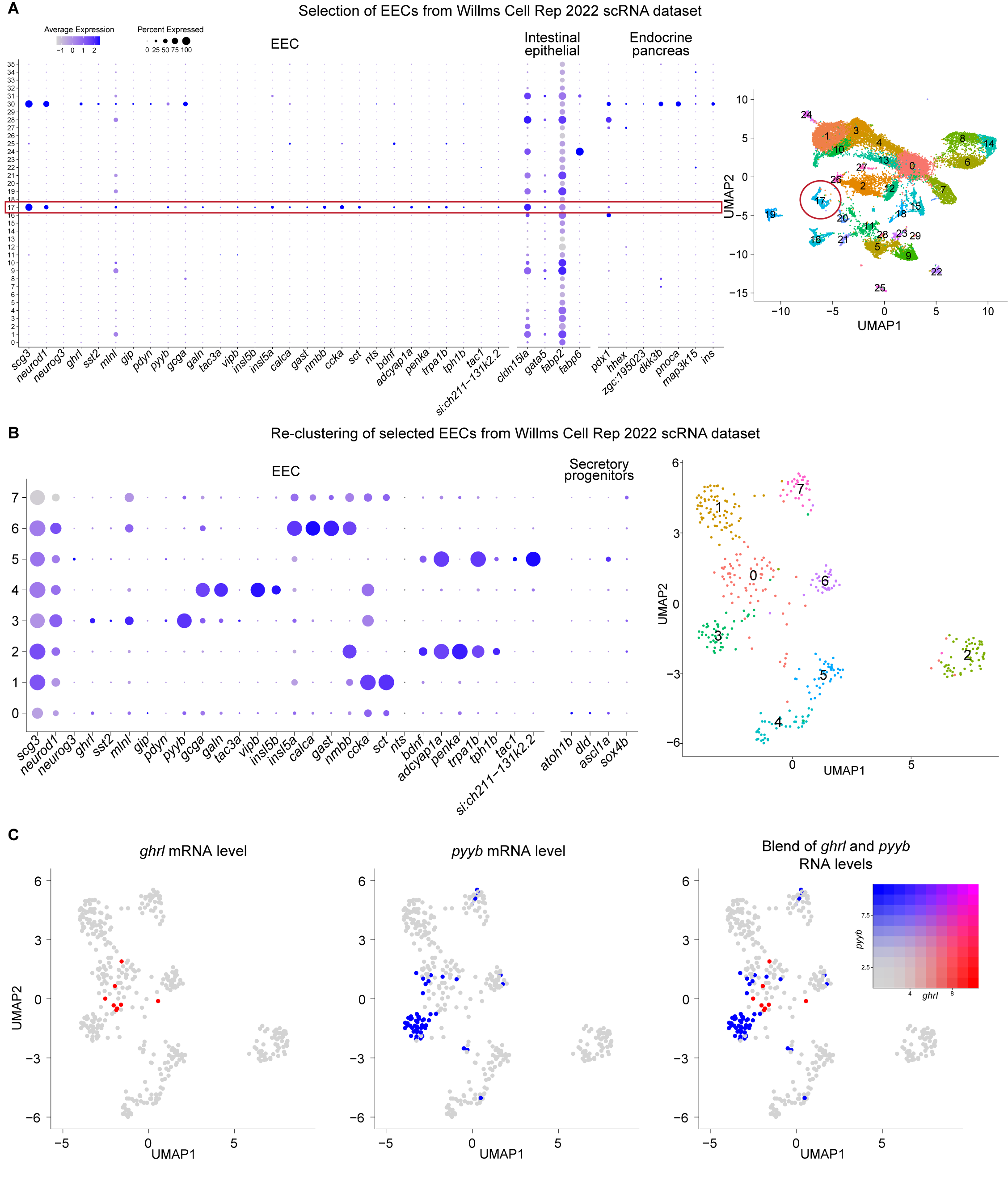

Supplement: S3 Fig — (A) scRNA-seq data of the larval zebrafish digestive tract from [56] was clustered and evaluated for expression of enteroendocrine cell (EEC) markers. The cluster showing strong expression of these markers and intestinal epithelial markers, but minimal pancreatic endocrine cell markers was selected for subsequent analysis and is outlined with a red box on the dotplot and red circle on the UMAP. (B) Those selected EECs were then re-clustered to identify EEC subtypes by examining EEC marker expression. (C) Seurat Feature Plot with simultaneous visualization of ghrl (red) and pyyb (blue) expression using the blend function, where overlapping expression would be shown in pink according to the relative expression scale shown. These plots show that, while both genes are expressed in the same cluster in this analysis, they label distinct populations of cells within that cluster. Underlying data can be found in S1 Data. (TIF) [file pbio.3003522.s008.tif]

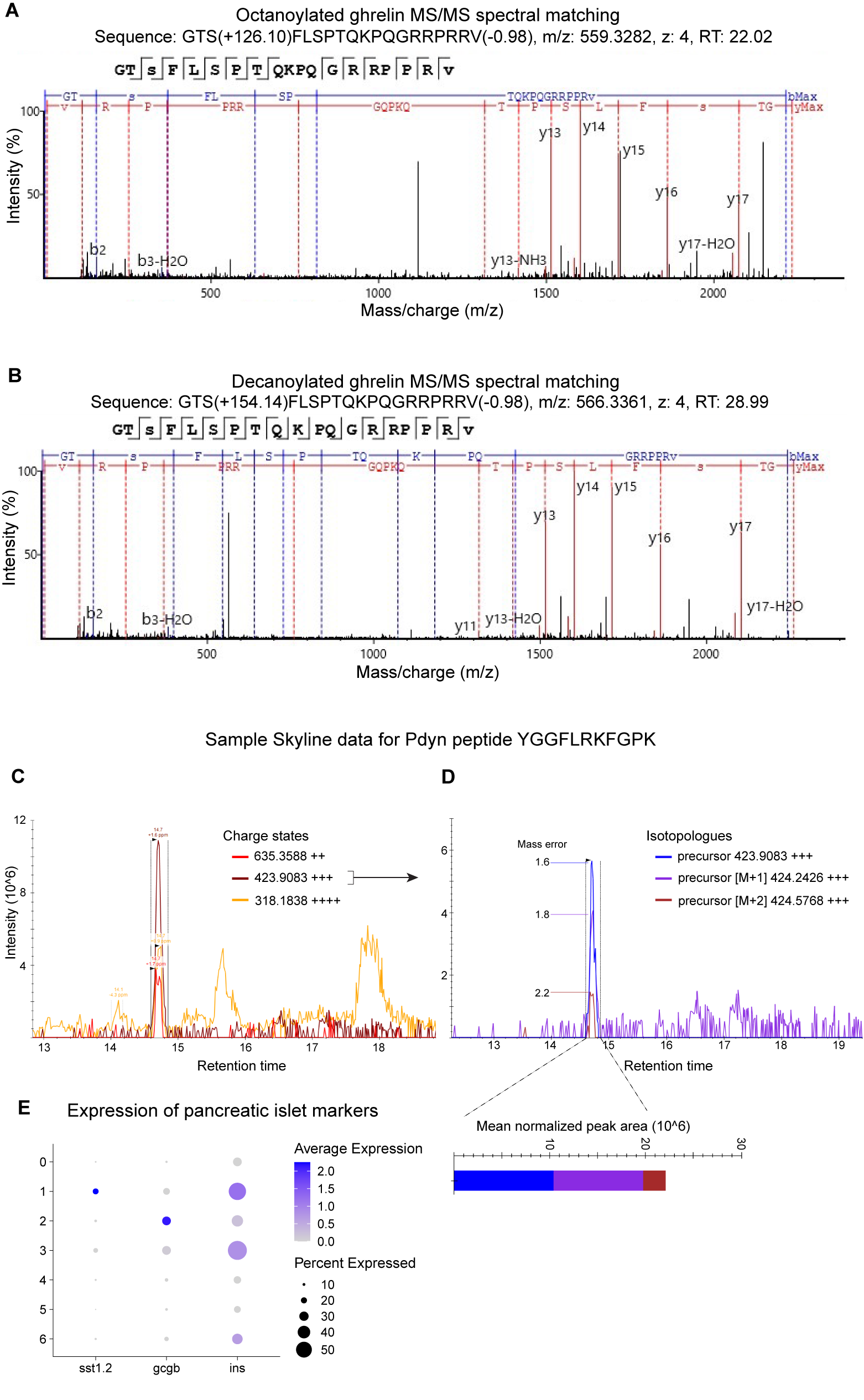

Supplement: S5 Fig — (A) Fragmentation spectrum identifying octanolylated ghrelin peptide. (B) Fragmentation spectrum identifying decanolylated ghrelin peptide. (C) Chromatogram data showing overlapping peaks for double, triple, and quadruple charged YGGFLRKFGPK peptide manually identified from the Pdyn protein. (D) Chromatogram data showing overlapping peaks for isotopologues M, M + 1, and M + 2 of the +3 charged peptide. Peak areas are quantified below and mass error for each is shown. (E) Expression in the joint larval and adult EEC dataset of hormone genes commonly enriched in the pancreatic islet. Underlying data can be found in S1 Data. (TIF) [file pbio.3003522.s010.tif]

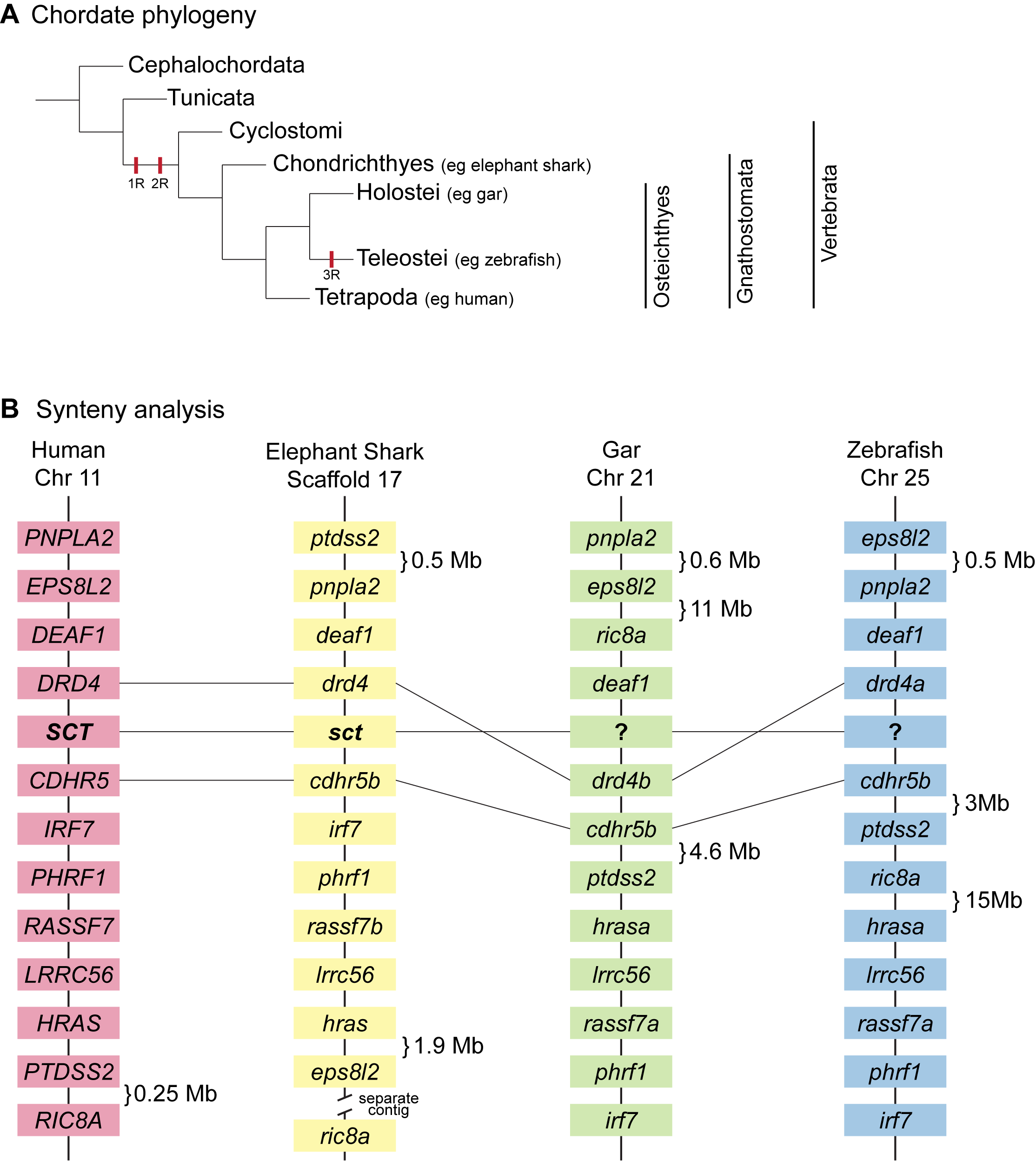

Supplement: S6 Fig — (A) Two rounds of whole genome duplication (labeled 1R and 2R) preceded the divergence of vertebrates [204–207]. The most ancient split within vertebrates is between the jawless Cyclostomi, such as lamprey, and the remaining Gnathostomata. The jawed vertebrates can be divided into those with primarily cartilaginous (Chondrichthyes) or bony (Osteichthyes) skeletons. A final round of whole genome duplication (labeled 3R) occurred in teleost fishes [189,190]. (B) The arrangement of genes along human chromosome 11, elephant shark scaffold 17, gar Chromosome 21, and zebrafish chromosome 25 are shown with genes ordered by ascending start location. Genes were identified by searching the ~250,000 base pairs upstream and downstream of human SCT and cross-checking with orthologs in zebrafish. Any gap equal to or greater than 0.5 Mega bases is annotated. The location of SCT and its direct neighbors are traced between each species with lines. Exact location of each gene is shown in S3 Table. (TIF) [file pbio.3003522.s011.tif]

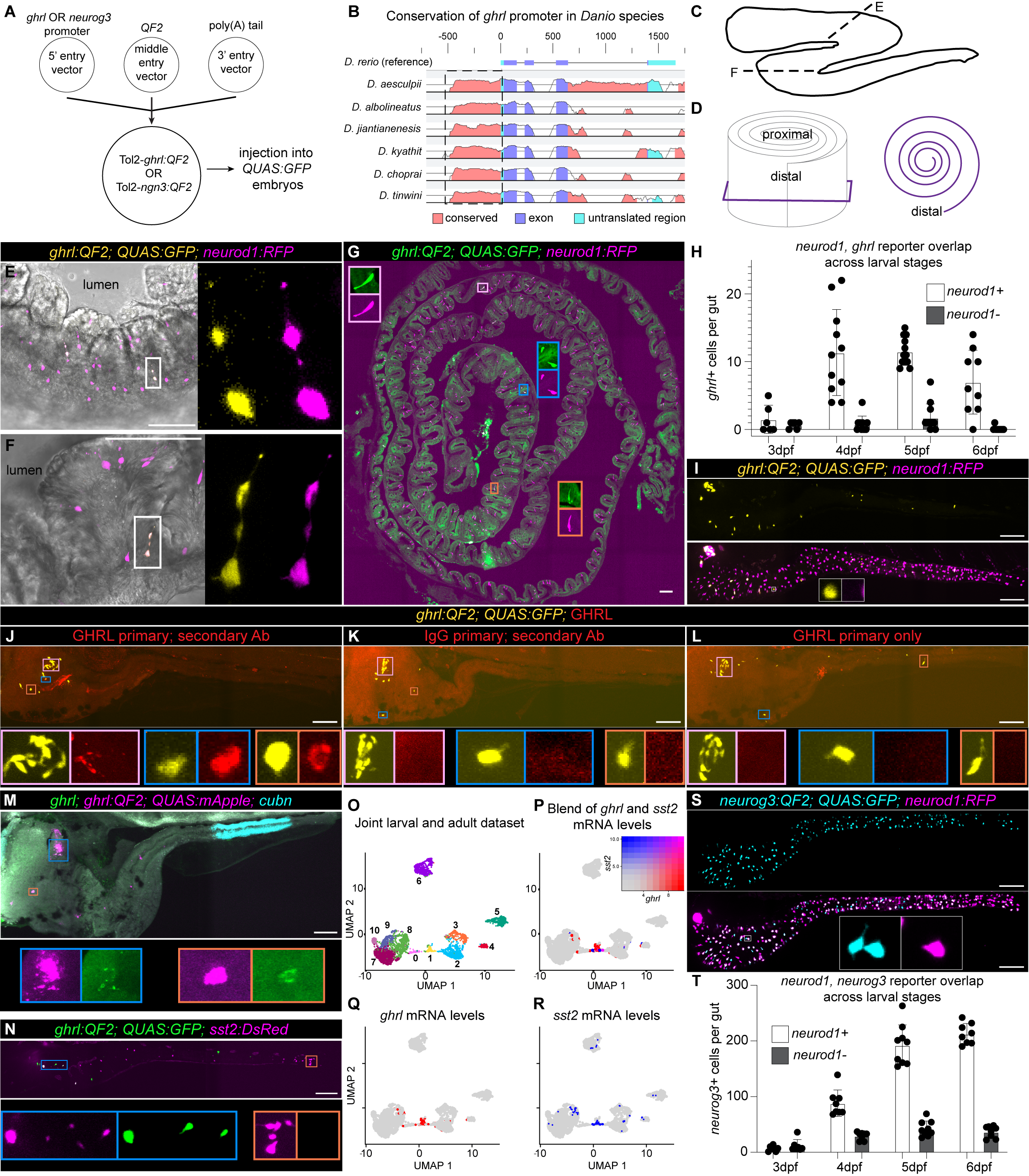

Supplement: S7 Fig — (A) Schematic representation of cloning approach to generating neurog3:QF2 and ghrl:QF2 reporters. (B) mVISTA [178,179] alignment of the ghrl locus in six closely related Danio species. Danio rerio ghrl annotation is shown at the top and is used as the reference. Dashed lines mark the highly conserved 647 base pair region upstream of the ghrl transcriptional start site that was cloned for the ghrl:QF2 reporter. (C) Schematic of where representative cross sections were taken to evaluate the ghrl:QF2 reporter in adult intestines in panels E and F. (D) Schematic of Swiss roll preparation and sectioning of full-length intestine from ghrl:QF2 reporter adults in panel G. (E) Representative image of a proximal intestinal section showing ghrl+; neurod1+ cells with classical EEC morphology. (F) Representative image of ghrl+; neurod1+ cell with classical EEC morphology in a distal intestinal section. (G) Section of Swiss-rolled adult ghrl:QF2 reporter intestine where the proximal gut is closest to the center. Several ghrl+; neurod1+ cells are highlighted. (H) Quantification of ghrl+ cells overlap with the pan-EEC reporter neurod1:RFP across larval development. Each dot represents an individual fish. (I) A representative image of a 5 dpf ghrl:QF2 fish with a highlighted example of a ghrl+; neurod1- cell. (J) Staining of 6 dpf larvae with anti-Ghrelin primary antibody and appropriate secondary antibody co-labels cells with ghrl:QF2 reporter in the pancreatic islet and intestine. While staining is not very strong, it is clearly stronger than negative controls run with (K) non-specific anti-IgG primary antibody and appropriate secondary antibody or (L) anti-Ghrelin primary antibody and no secondary antibody. (M) ghrl:QF2 reporter co-labels with hybridization chain reaction (HCR) probes targeting ghrl mRNA in the islet and intestine. HCR probes targeting cubn, a gene enriched in the lysosome-rich enterocytes (LREs) in the distal intestine, serve as a positive control for the HCR t [file pbio.3003522.s012.tif]

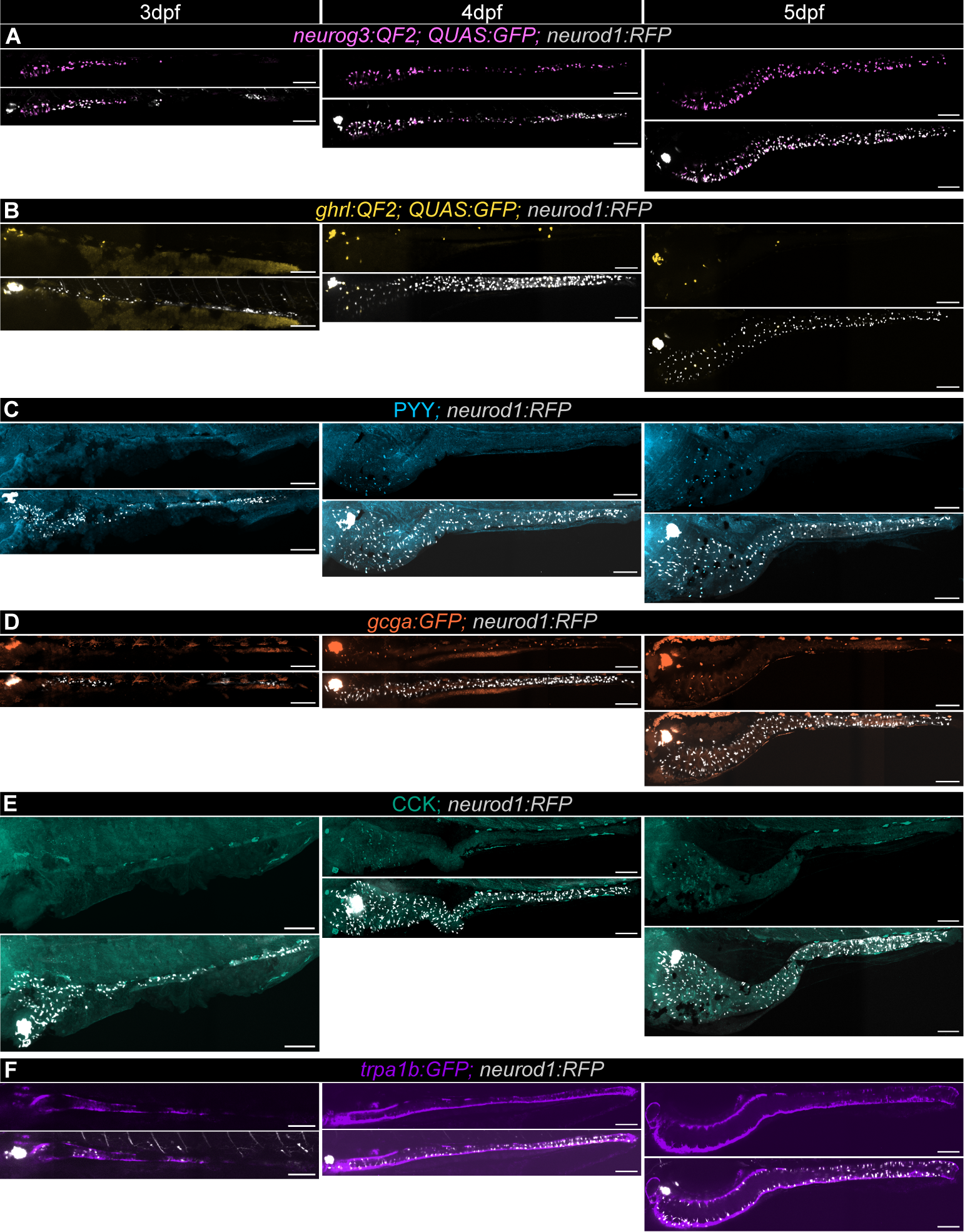

Supplement: S8 Fig — Representative images showing subtype distribution and overlap with pan-EEC neurod1 reporter at 3, 4, and 5 dpf for (A) neurog3 reporter, (B) ghrl reporter, (C) anti-PYY antibody, (D) gcga reporter, (E) anti-CCK antibody, (F) trpa1b reporter. Scale bars are 100 μm. (TIF) [file pbio.3003522.s013.tif]

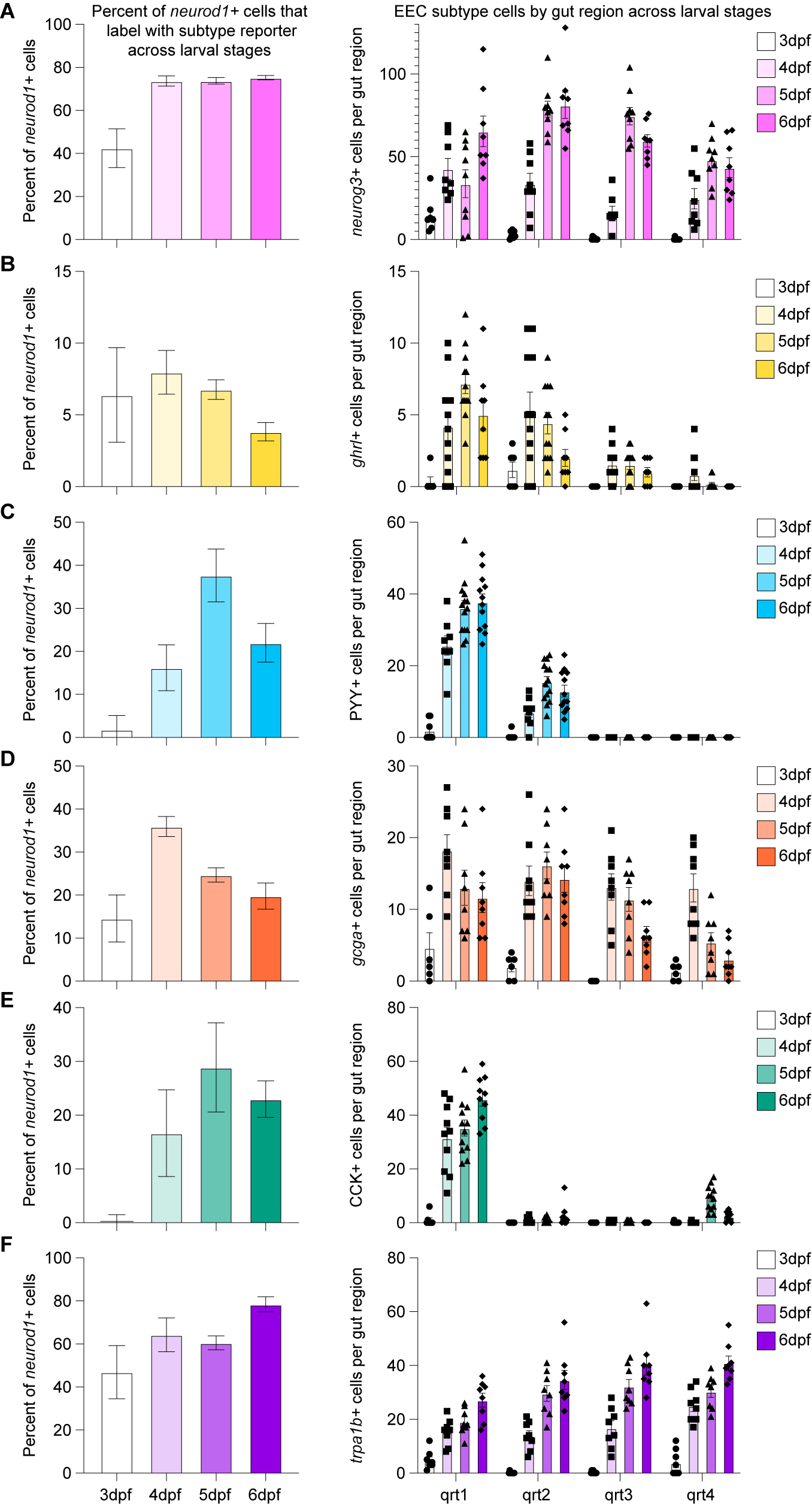

Supplement: S9 Fig — The percent of neurod1+ cells that label with the subtype reporter and the raw counts of subtype numbers per each equal-length quarter of the gut is shown for 3, 4, 5, and 6 days post fertilization (dpf) fish in the (A) neurog3 reporter, (B) ghrl reporter, (C) anti-PYY antibody, (D) gcga reporter, (E) anti-CCK antibody, and (F) trpa1b reporter. Each dot represents a fish. Underlying data can be found in S1 Data. (TIF) [file pbio.3003522.s014.tif]

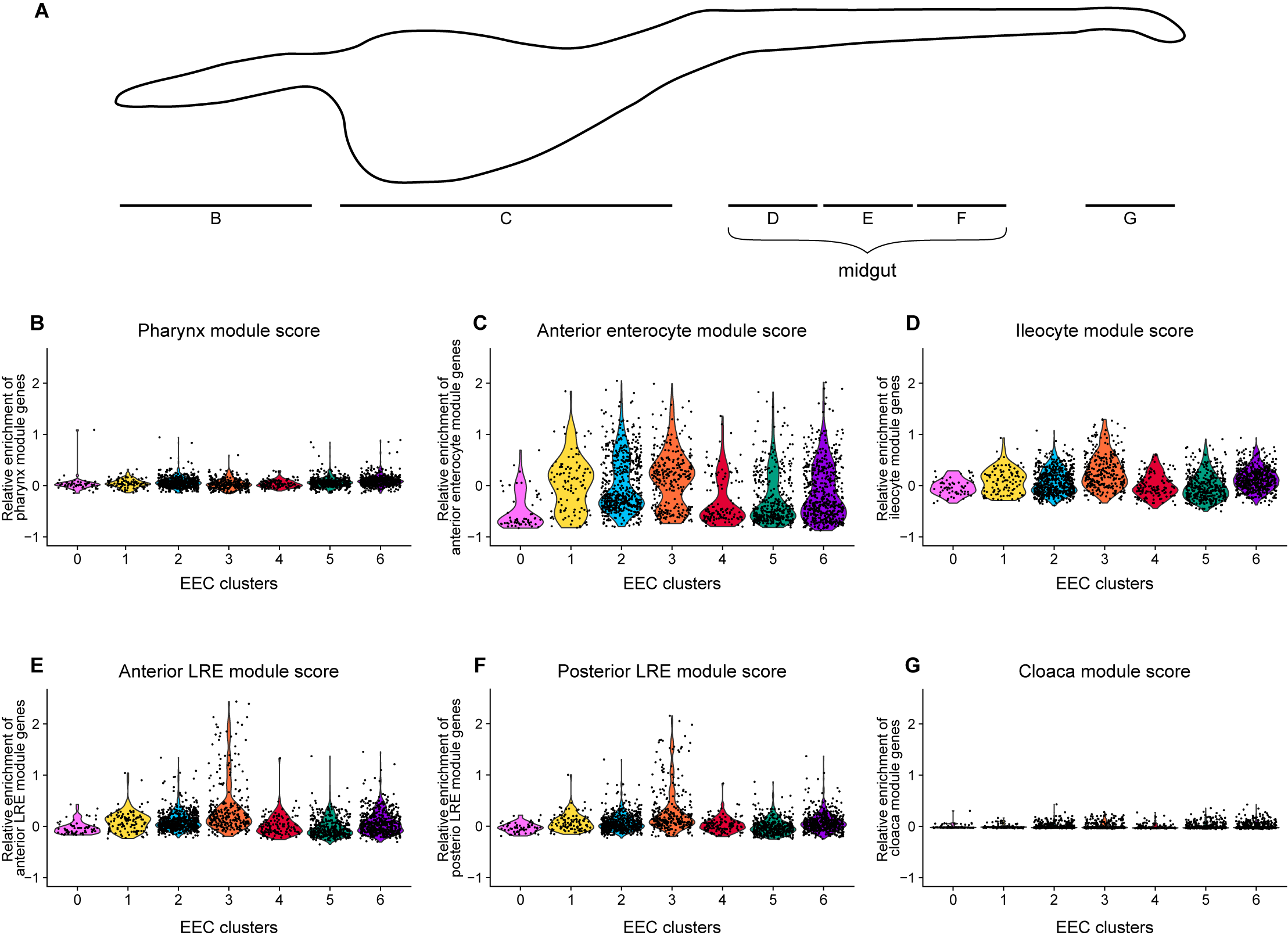

Supplement: S10 Fig — (A) Cartoon of larval zebrafish intestine. Region-specific genes along the intestinal epithelium were determined using the scRNA-seq dataset from [109]. Horizontal bars below the cartoon represent the approximated anteroposterior location of a given regional cell type and are marked with the panel letter that examines expression of genes specific to that region. Module scores in our combined larval and adult scRNA-seq dataset of the expression of the 25 most enriched genes in the (B) pharynx, (C) anterior enterocytes, (D) ileocytes, (E) anterior lysosome-rich enterocytes (LREs), (F) posterior LREs, and (G) cloaca are shown. Underlying data can be found in S1 Data. (TIF) [file pbio.3003522.s015.tif]

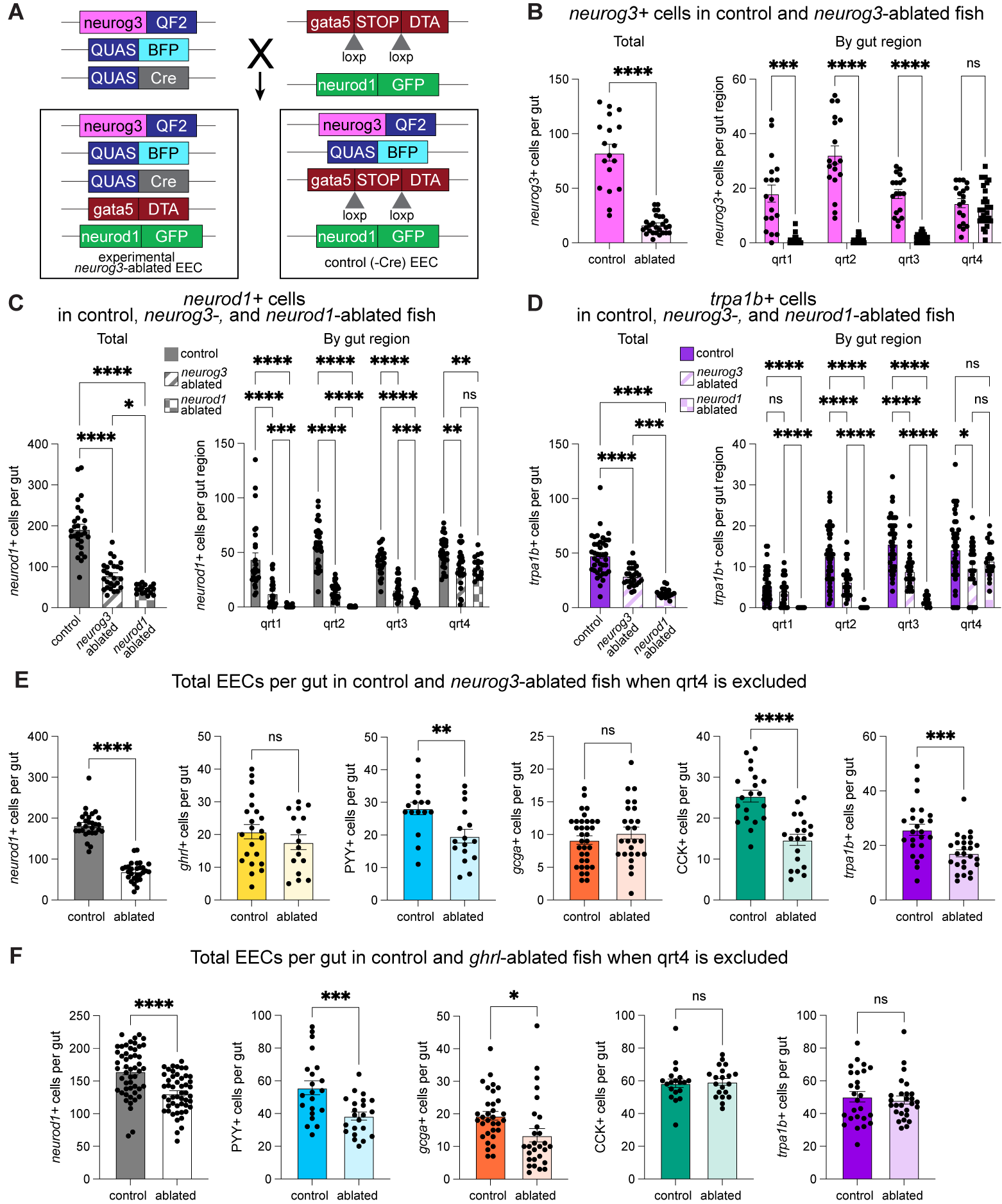

Supplement: S11 Fig — (A) Schematic of neurog3+ ablation combined with neurog3+, neurod1+ cell labeling to determine neurog3+ ablation efficiency. (B) Total and regional counts of neurog3+ cells in neurog3+ ablation. (C) Total and regional counts of neurod1+ cells in control, neurog3+, and neurod1+ ablation. (D) Total and regional counts of trpa1b+ cells in control, neurog3+, and neurod1+ ablation. (E) Total EEC and subtype counts in control and neurog3+ ablated fish when qrt4 cells are excluded. Counts that include qrt4 are shown in Fig 4C–4H. (F) Total EEC and subtype counts in control and ghrl-ablated fish when qrt4 cells are excluded. Counts that include qrt4 are shown in Fig 5B–5F. Underlying data can be found in S1 Data. (TIF) [file pbio.3003522.s016.tif]

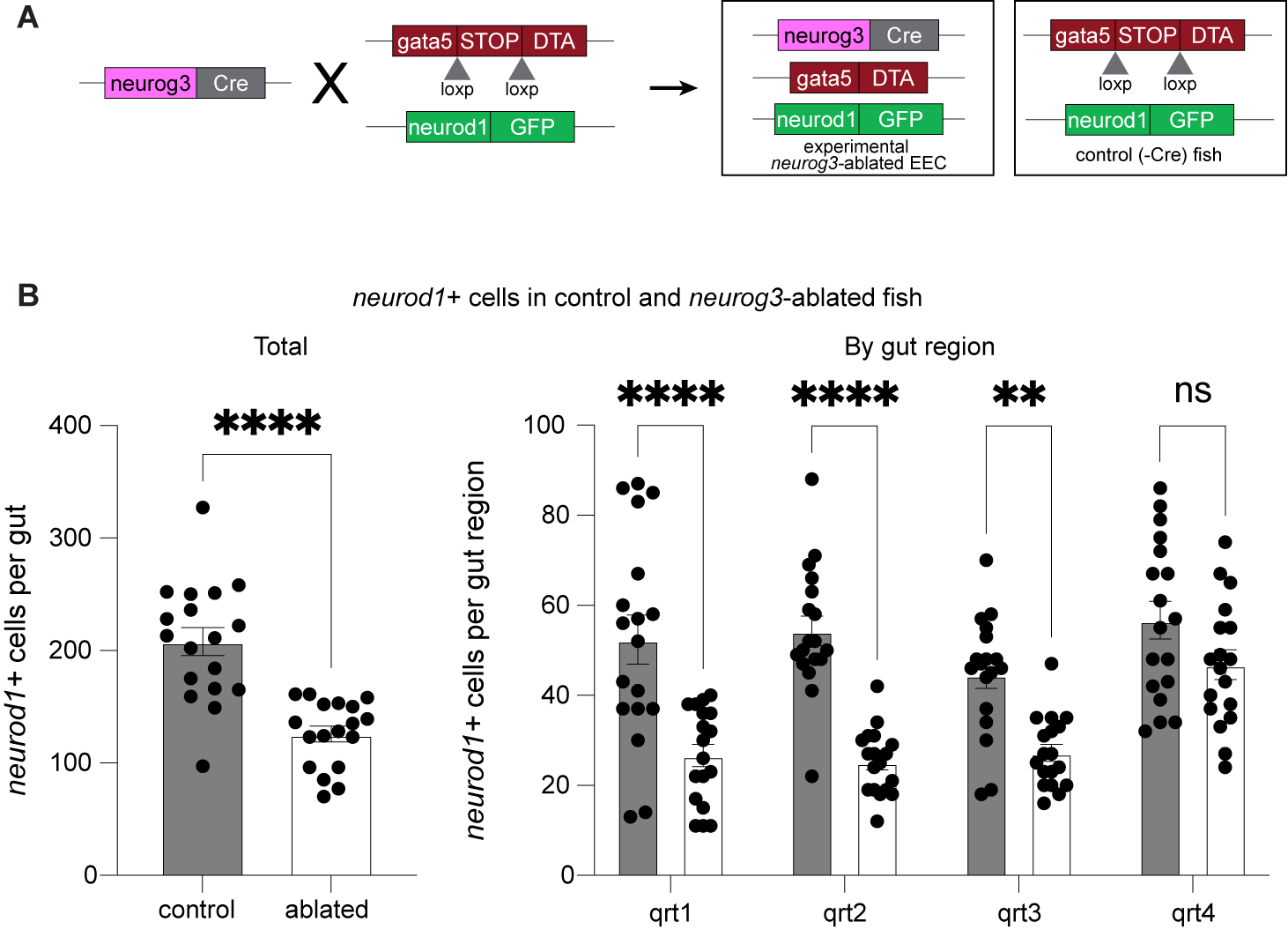

Supplement: S12 Fig — (A) Schematic of neurog3:Cre induced neurog3+ cell ablation. (B) Total counts and regional counts of neurod1+ cells in the neurog3:Cre-ablated and control fish. Each dot represents a 6-day post-fertilization fish. Statistical significance was calculated by unpaired t test for total cell numbers and by two-way ANOVA for regional analysis. Significance annotations are as follows: ns (p > 0.05), * (p < 0.05), ** (p < 0.01), *** (p < 0.001), **** (p < 0.0001). Underlying data can be found in S1 Data. (TIF) [file pbio.3003522.s017.tif]

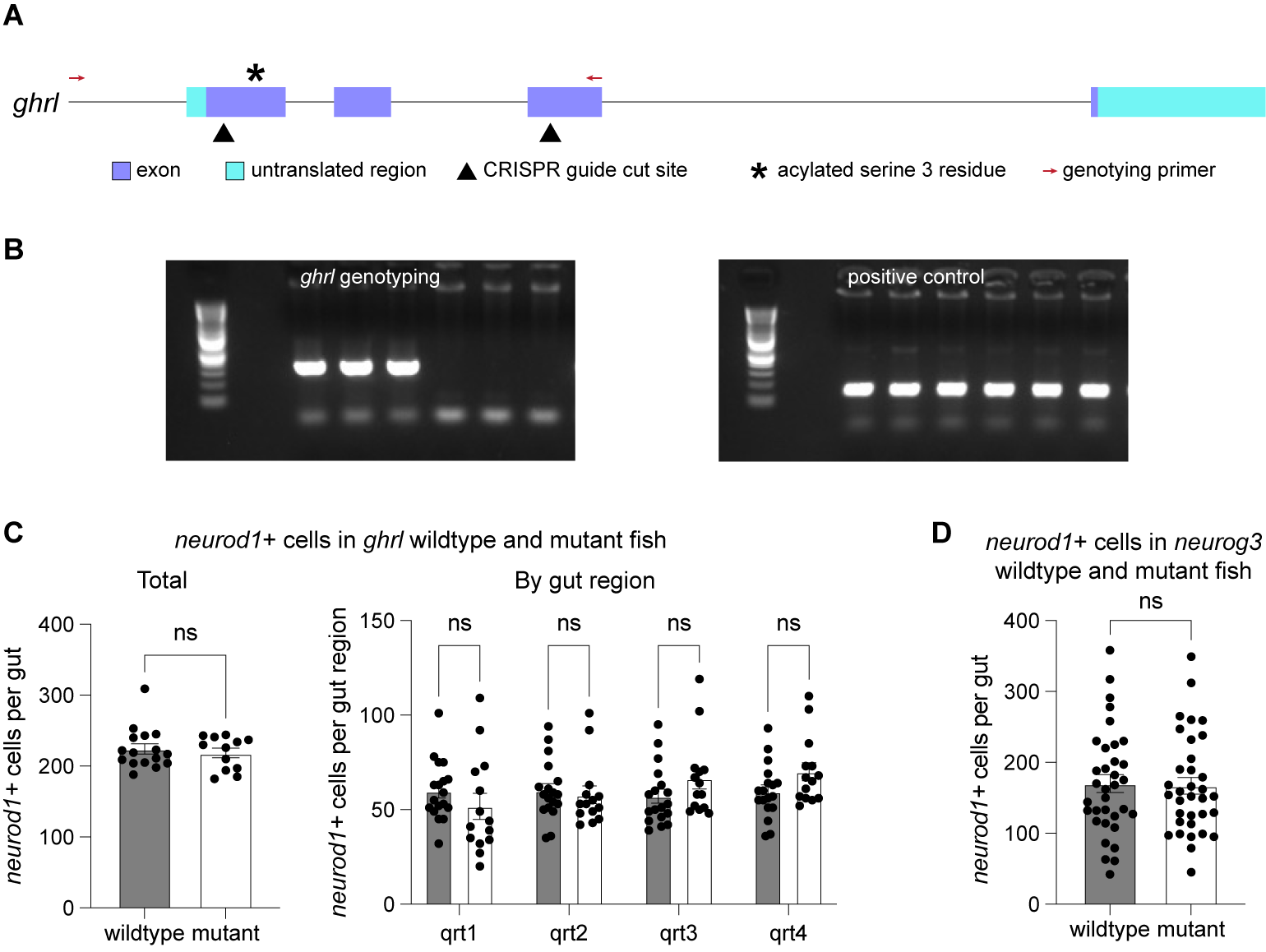

Supplement: S13 Fig — (A) Schematic of the ghrl locus with arrowheads marking the sites targeted with CRISPR guide RNAs. (B) Genotyping and control gels run with 1 kb+ NEB ladder and 3 ghrl mutant and 3 ghrl wildtype samples. Amplification with genotyping primers (red arrows) across the 804 base pair region results in a 300 base pair band due to deletion in mutants (lanes 1–3). Wildtype samples (lanes 4–6) do not amplify due to the highly repetitive nature of intron 2, but all samples amplified with control primers amplifying at a non-affected locus (mttp gene). (C) Total counts and regional counts of neurod1+ cells in ghrl wildtype (heterozygous) and ghrl mutant (homozygous) fish. (D) Total counts of neurod1+ cells in neurog3 wildtype (homozygous) and neurog3 mutant (homozygous) fish. Each dot represents a 5–6-day post-fertilization fish. Statistical significance was calculated by unpaired t test for total cell numbers and by two-way ANOVA for regional analysis. Underlying data can be found in S1 Data. (TIF) [file pbio.3003522.s018.tif]

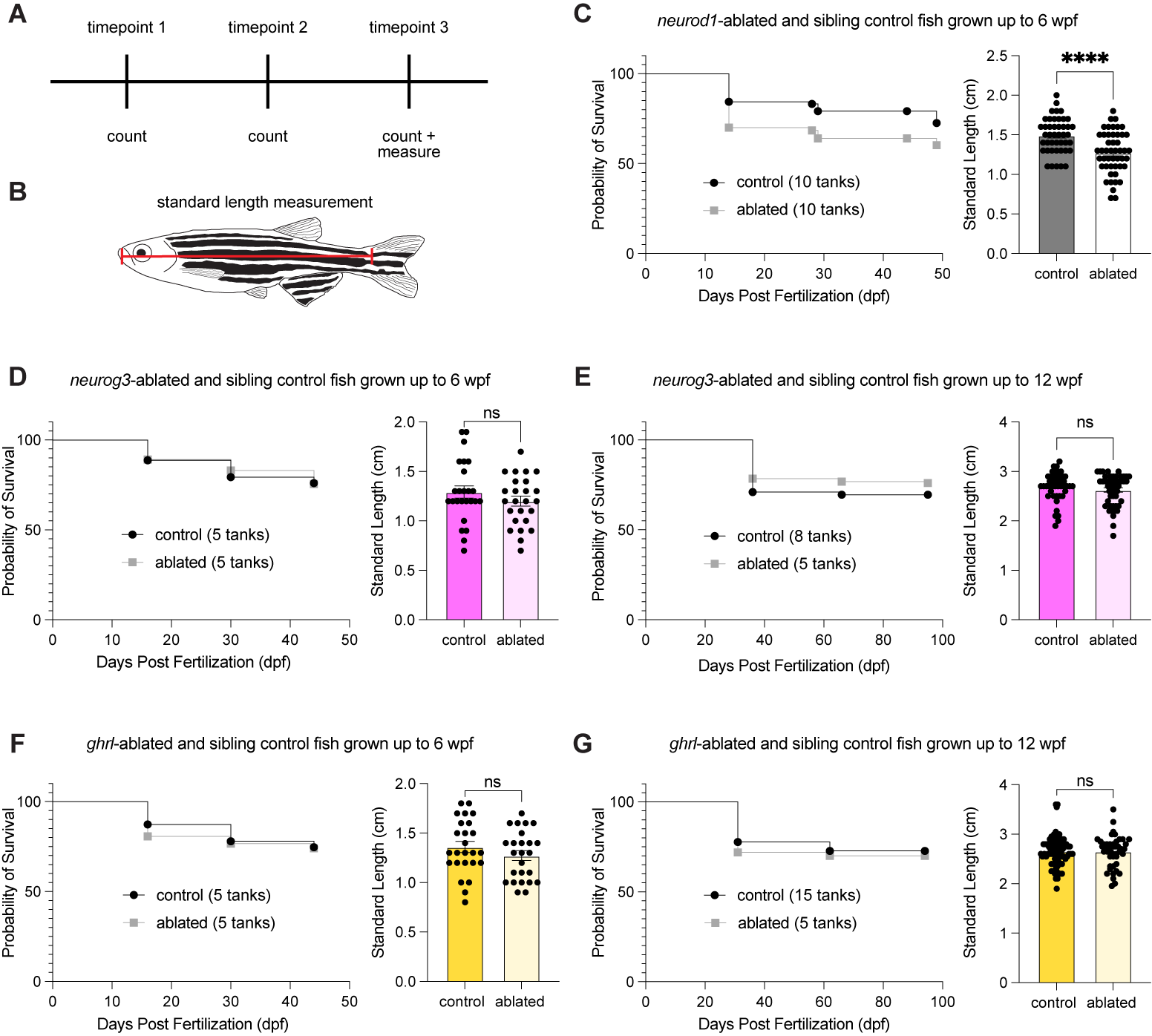

Supplement: S14 Fig — (A) Schematic of adult experiments demonstrating when survival counts and standard length measurements were taken. (B) Schematic of standard length measurements performed at the endpoint of each experiment. (C) neurod1-ablated versus sibling control survival and growth data from two independent experiments, each carried out over 6 weeks. Survival curves are significantly different by Mantel-Cox log-rank test (P = 0.0006). (D) neurog3-ablated versus sibling controls followed for 6 weeks showed no significant difference in survival or growth. (E) neurog3-ablated versus sibling controls followed for 12 weeks again showed no significant differences in survival or growth. (F) ghrl-ablated versus sibling controls followed for 6 weeks showed no significant difference in survival or growth. (G) ghrl-ablated versus sibling controls followed for 12 weeks again showed no significant differences in survival or growth. Survival analyses were performed on the pooled results across the number of tanks specified in each panel. Statistical significance was calculated by Mantel-Cox log-rank test for survival curves and by unpaired t test for standard length. Underlying data can be found in S1 Data. (TIF) [file pbio.3003522.s019.tif]

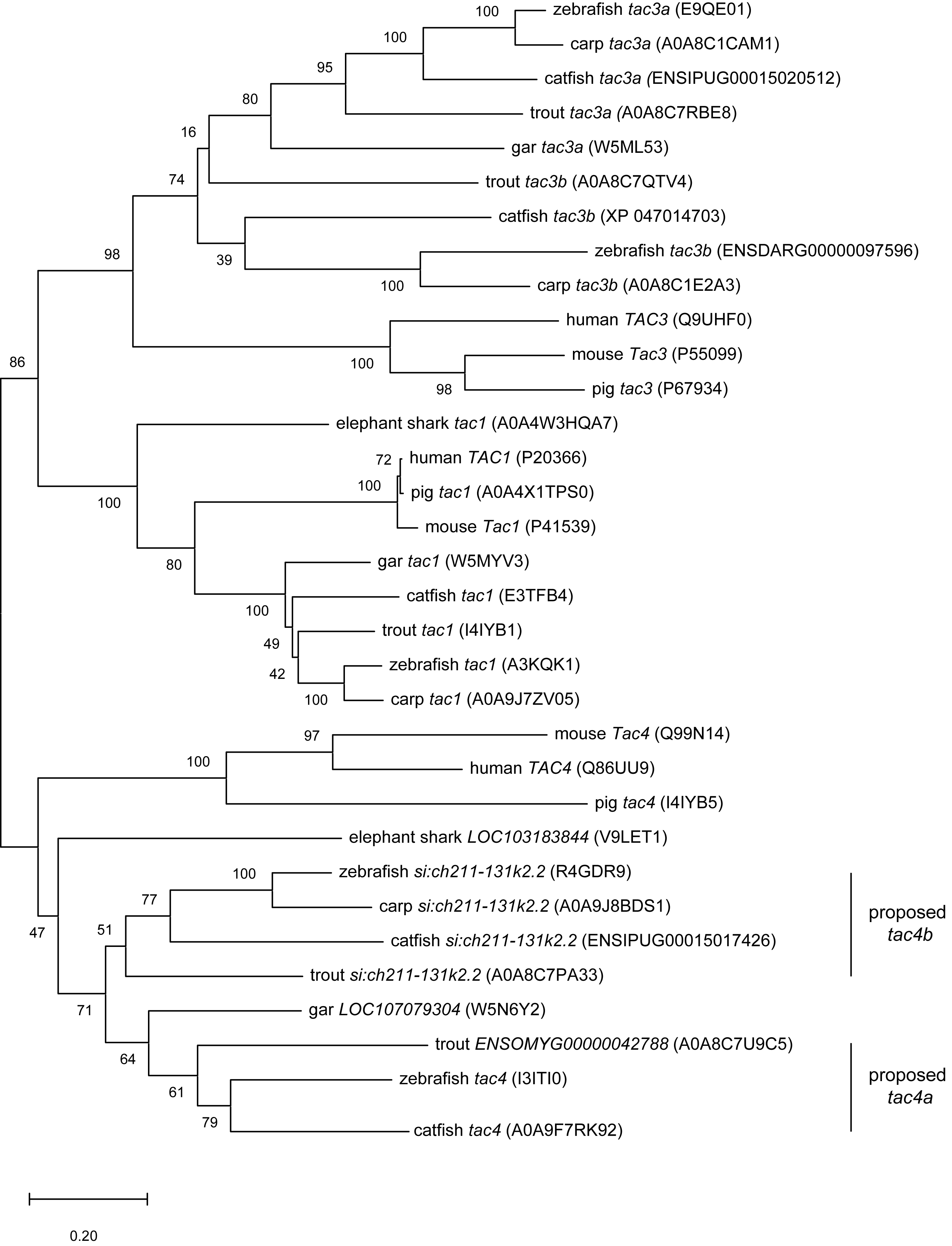

Supplement: S15 Fig — The analytical procedure encompassed 33 amino acid sequences of tachykinin proteins identified from zebrafish (Danio rerio), common carp (Cyprinus carpio carpio), catfish (Ictalurus punctatus), rainbow trout (Oncorhynchus mykiss), spotted gar (Lepisosteus oculatus), elephant shark (Callorhinchus milii), pig (Sus scrofa), mouse (Mus musculus), and human (Homo sapiens). The gene name and accession number are shown for each branch. Proposed tachykinin family names are shown for those genes without one. The evolutionary history was inferred using the Neighbor-Joining method [201]. The optimal tree with the sum of branch length = 11.694 is shown. The percentage of replicate trees in which the associated taxa clustered together in the bootstrap test (100 replicates) are shown next to the branches [208]. The tree is drawn to scale, with branch lengths in the same units as those of the evolutionary distances used to infer the phylogenetic tree. The evolutionary distances were computed using the Poisson correction method [209] and are in the units of the number of amino acid substitutions per site. The pairwise deletion option was applied to all ambiguous positions for each sequence pair resulting in a final data set comprising 226 positions. Evolutionary analyses were conducted in MEGA12 [200,210] utilizing up to 4 parallel computing threads. (TIF) [file pbio.3003522.s020.tif]

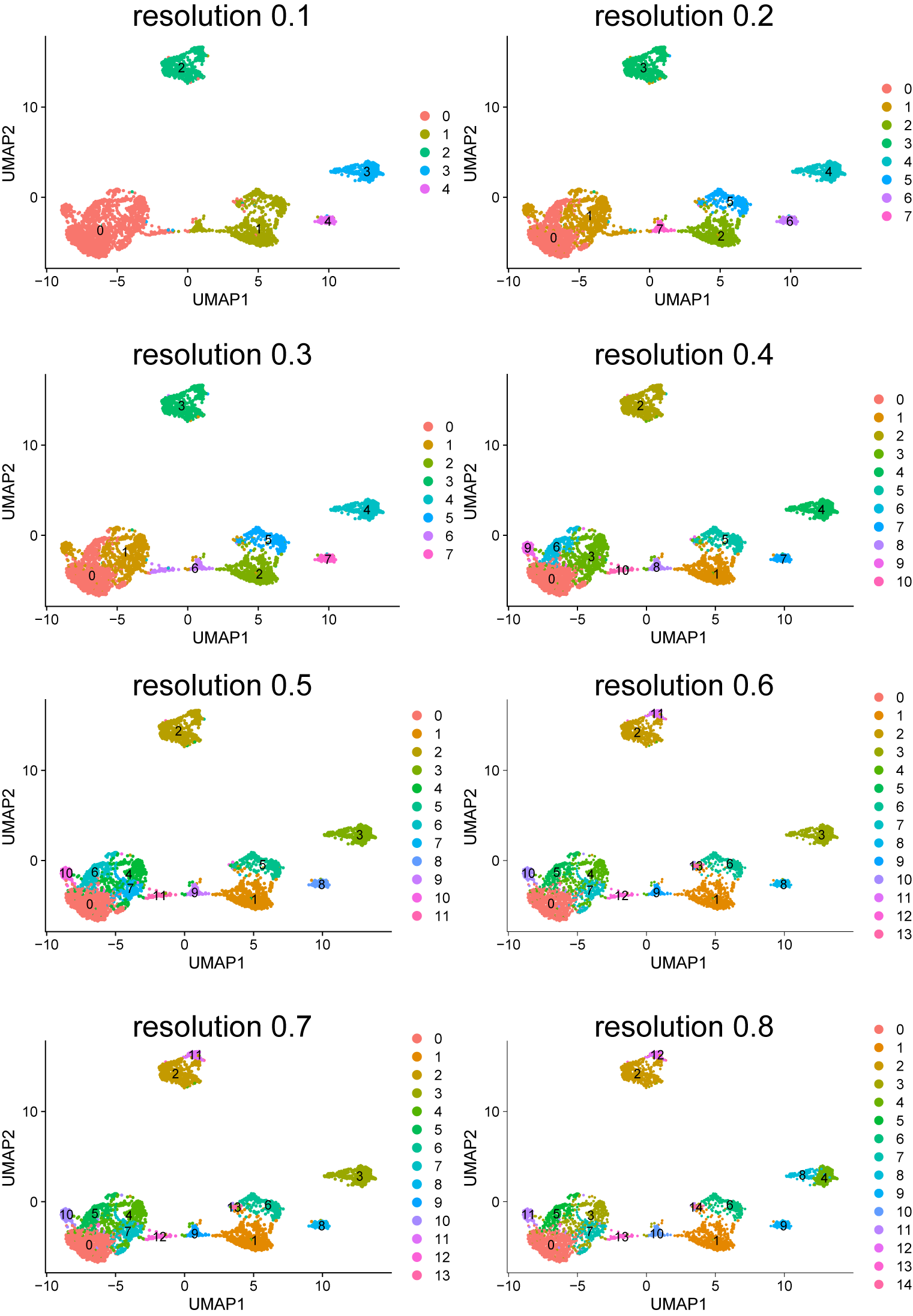

Supplement: S16 Fig — Clustering for the scRNA-seq dataset presented in Fig 1 is shown here at resolution 0.1–0.8. The resolution of 0.4 was used for this study. (TIF) [file pbio.3003522.s021.tif]

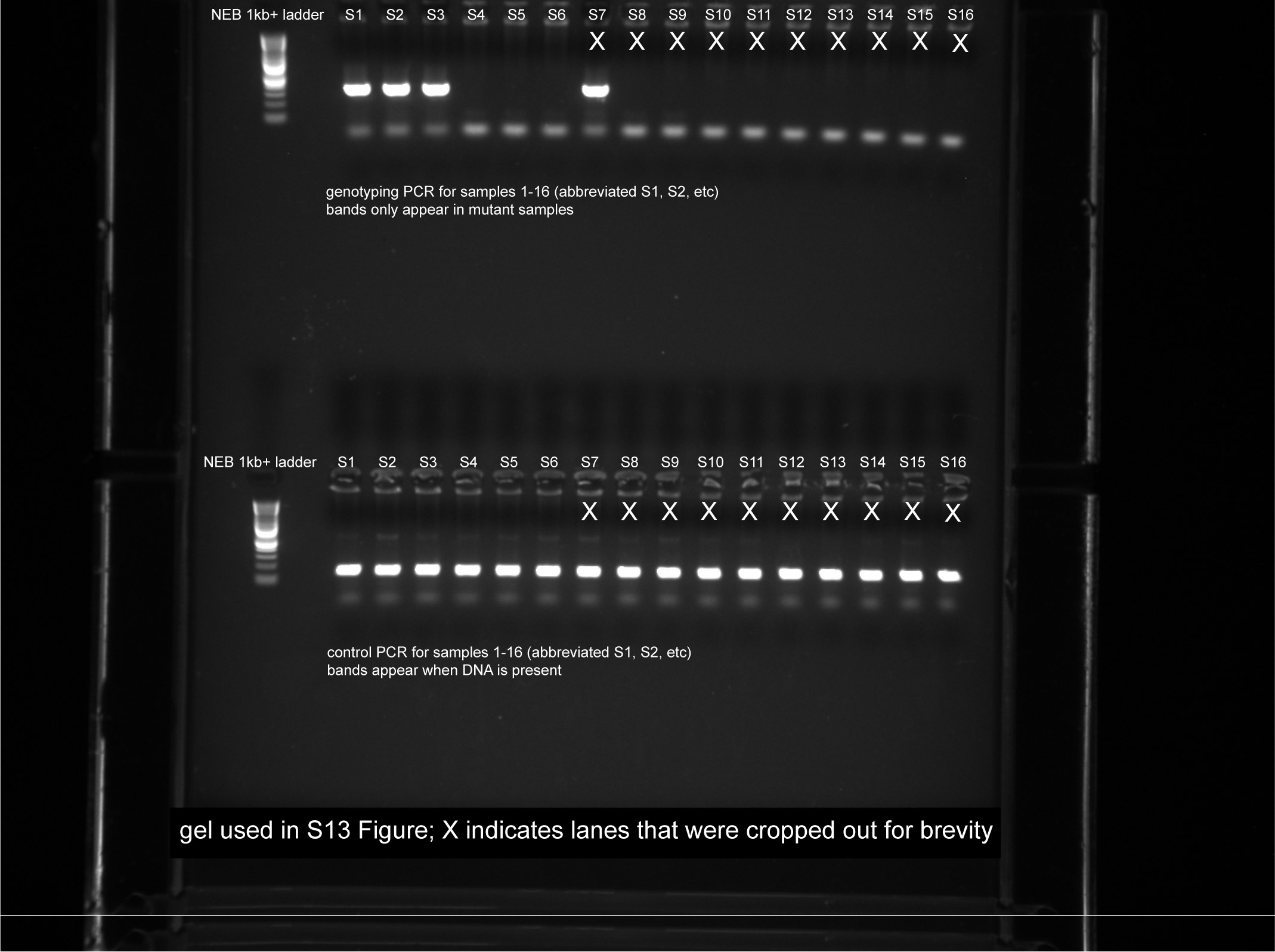

Supplement: S1 Raw Images — (TIF) [file pbio.3003522.s022.tif]
