# Supplementary material for: Identification of intestinal enteroendocrine cell subtypes and their associated hormones in zebrafish
Source: PLoS Biol. 2025 Dec 18;23(12):e3003522. doi: 10.1371/journal.pbio.3003522 (PMC12714231; doi:10.1371/journal.pbio.3003522)
Supplement: S4 Fig — In all panels, the primary amino acid sequence of the gene of interest is shown at the top in black with any identified missense variants indicated in red above. Blue horizontal lines below the amino acid sequence represent the unique peptides detected in our study with small black vertical lines denoting the stop and start of each peptide. Abutting peptides share a single vertical line to represent the stop of the preceding peptide and the start of the subsequent one. Different colored squares represent various posttranslational modification detected. Shading labels regions aligning to Uniprot-annotated peptides in humans. In cases where multiple peptides are known to be processed from the same sequence, dashed lines indicate different cleavage sites. Below the detected peptides is a multispecies protein alignment where amino acids are color-coded based on their percent identity match across all the reported species with darker coloring indicating a more conserved residue. Human processed peptide annotations taken from Uniprot are labeled below with horizontal lines. The color of these lines corresponds to the color of the shading of the aligned zebrafish amino acids above. Note that many of the peptide cleavage sites occur at dibasic residues (i.e., RR/KR/KK), consistent with cleavage by prohormone convertase enzymes [148]. More detailed information about the peptides shown in this figure are available in S2 Table. (A) Zebrafish adcyap1a-derived peptides detected in EECs. Alignment of the primary amino acid sequence of Pituitary adenylate cyclase-activating polypeptide protein includes zebrafish (Uniprot Q98TU3), trout (Uniprot A0A8C7QBD4), goldfish (Uniprot A0A6P6RC36), catfish (Uniprot Q90XZ4), chicken (Uniprot Q58FG9), mouse (Uniprot O70176), rat (Uniprot A6KFB2), human (Uniprot P18509). (B) Zebrafish gcga- and gcgb-derived peptides detected in EECs. As gcga and gcgb are both orthologs of human GCG, they are reported together. Peptides aligning to multiple isof [file pbio.3003522.s009.pdf]

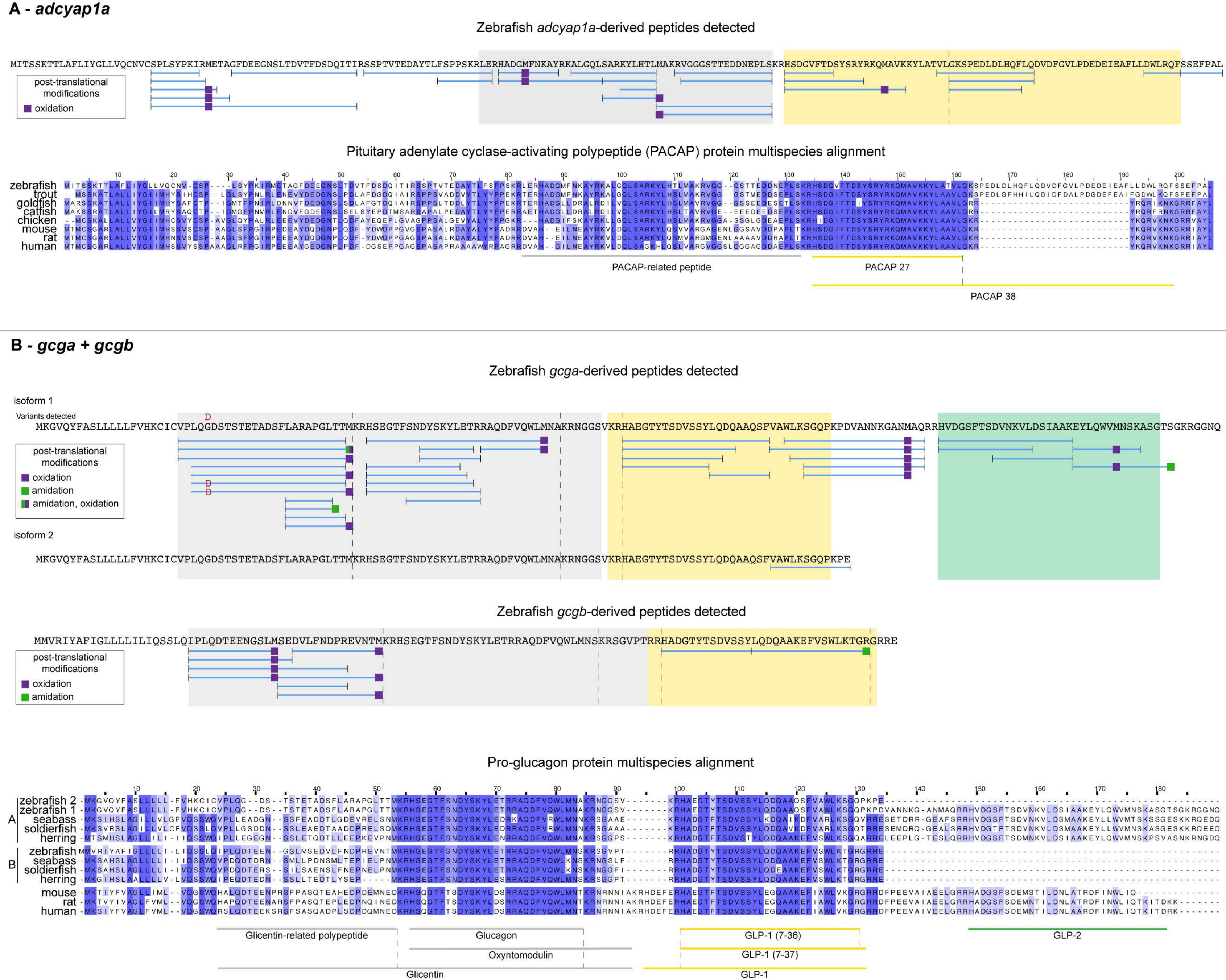
A - adcyap1a
Zebrafish adcyap1a-derived peptides detected
MITSSKTTLAFLIYGLLVQCNVCSPLSYPKIRMETAGFDEEGNSLTDVTFDSDQITIRSSPTVTEDAYTLFSPPSKRLERHADGMFNKAYRKALGQLSARKYLHTLMAKRVGGGSTTEDDNEPLSKRHSDGVFTDSYSRYRKQMAVKKYLATVLGKSPEDLDLHQFLQDVDFGVLPDEDEIEAFLLDWLRQFSSEFPAL
post-translational modifications
oxidation
Pituitary adenylate cyclase-activating polypeptide (PACAP) protein multispecies alignment
zebrafish
trout
goldfish
catfish
chicken
mouse
rat
human
PACAP-related peptide
PACAP 27
PACAP 38
B - gcga + gcgb
Zebrafish gcga-derived peptides detected
isoform 1
Variants detected
MKGVQYFASLLLLLFVHKCICVPLQGDSTSTETADSFLARAPGLTTMKRHSEGTFSNDYSKYLETRRAQDFVQWLMNAKRNGGSVKRHAEGTYTSDVSSYLQDQAAQSFVAWLKSGQPKPDVANNKGANMAQRRHVDGSFTSDVNKVLDSIAAKEYLQWVMNSKASGTSGKRGGNQ
post-translational modifications
oxidation
amidation
amidation, oxidation
isoform 2
MKGVQYFASLLLLLFVHKCICVPLQGDSTSTETADSFLARAPGLTTMKRHSEGTFSNDYSKYLETRRAQDFVQWLMNAKRNGGSVKRHAEGTYTSDVSSYLQDQAAQSFVAWLKSGQPKPE
Zebrafish gcgb-derived peptides detected
MMVRIYAFIGLLLLILIQSSLQIPLQDTEENGSLMSEDVLFNDPREVNTMKRHSEGTFSNDYSKYLETRRAQDFVQWLMNSKRSGVPTRRHADGTYTSDVSSYLQDQAAKEFVSWLKTGRGRRE
post-translational modifications
oxidation
amidation
Pro-glucagon protein multispecies alignment
A
zebrafish 2
zebrafish 1
seabass
soldierfish
herring
B
zebrafish
seabass
soldierfish
herring
mouse
rat
human
Glicentin-related polypeptide
Glucagon
Oxyntomodulin
Glicentin
GLP-1 (7-36)
GLP-1 (7-37)
GLP-1
GLP-2

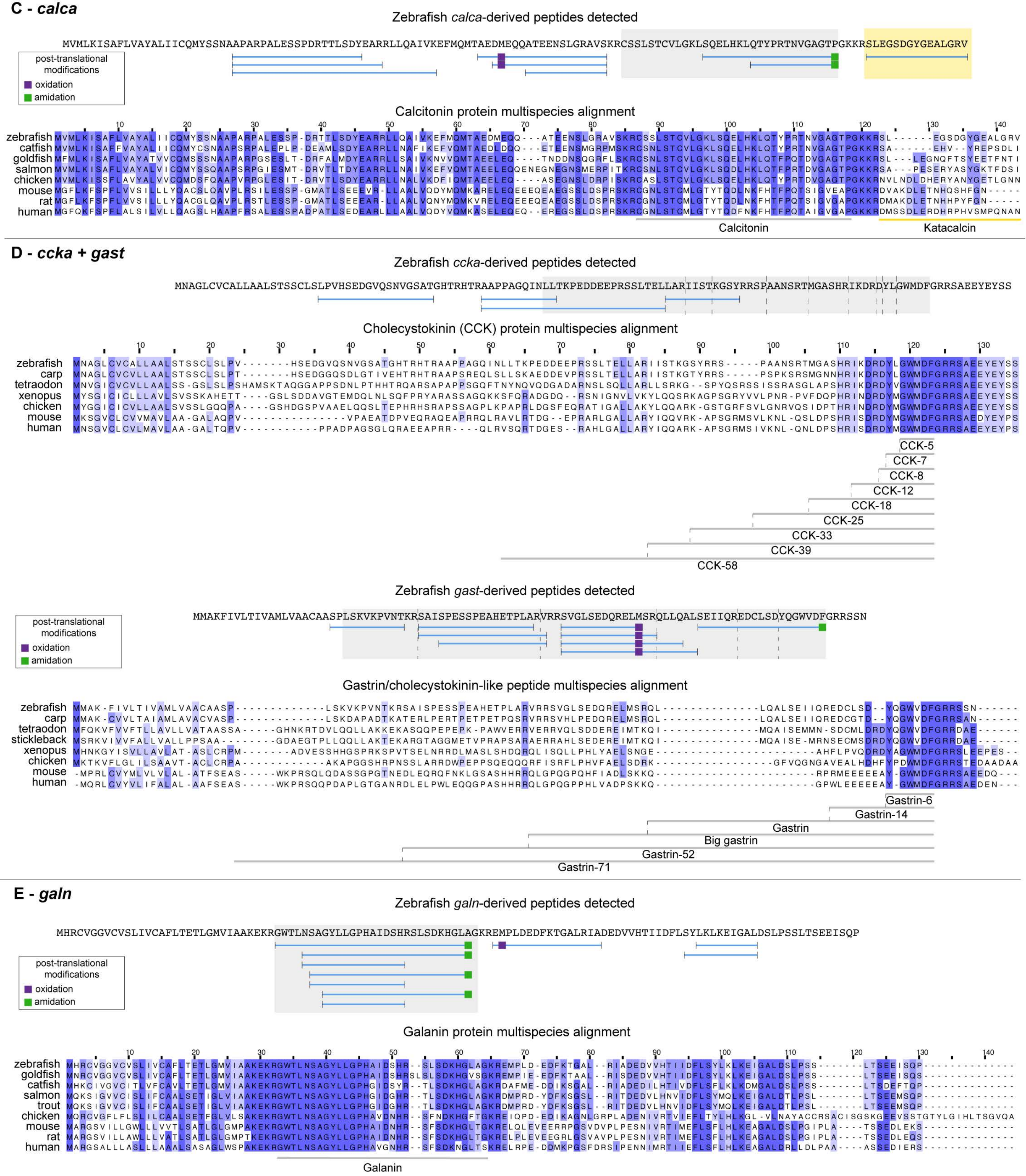
C - calca
Zebrafish calca-derived peptides detected
MVMLKISAFLVAYALIICQMYSSNAAPARPALESSPDRTTLSDYEARRLLQAIVKEFMQMTAEDMEQQATEENSLGRAVSKRCSSLSTCVLGKLSQELHKLQTYPRTNVGAGTPGKKRSLEGSDGYGEALGRV
post-translational modifications
oxidation
amidation
Calcitonin protein multispecies alignment
zebrafish
catfish
goldfish
salmon
chicken
mouse
rat
human
Calcitonin
Katacalcin
D - ccka + gast
Zebrafish ccka-derived peptides detected
MNAGLCVCALLAALSTSSCLSLPVHSEDGVQSNVGSATGHTRHTRAAPPAGQINLLTKPEDDEEPRSSLTELLARIISTKGSYRRSPAANSRTMGASHRIKDRDYLGWMDFGRRSAEEYEYSS
Cholecystokinin (CCK) protein multispecies alignment
zebrafish
carp
tetraodon
xenopus
chicken
mouse
human
CCK-5
CCK-7
CCK-8
CCK-12
CCK-18
CCK-25
CCK-33
CCK-39
CCK-58
Zebrafish gast-derived peptides detected
MMAKFIVLTIVAMLVAACAASPLSKVKPVNTKRSAISPESSPEAHETPLARVRRSVGLSEDQRELMSRQLLQALSEIIQREDCLSDYQGWVDFGRRSSN
post-translational modifications
oxidation
amidation
Gastrin/cholecystokinin-like peptide multispecies alignment
zebrafish
carp
tetraodon
stickleback
xenopus
chicken
mouse
human
Gastrin-6
Gastrin-14
Gastrin
Big gastrin
Gastrin-52
Gastrin-71
E - galn
Zebrafish galn-derived peptides detected
MHRCVGGVCVSLIVCAFLTETLGMVIAAKEKRGWTLNSAGYLLGPHAIDSHRSLSDKHGLAGKREMPLDEDFKTGALRIADEDVVHTIIDFLSYLKLKEIGALDSLPSSLTSEEISQP
post-translational modifications
oxidation
amidation
Galanin protein multispecies alignment
zebrafish
goldfish
catfish
salmon
trout
chicken
mouse
rat
human
Galanin

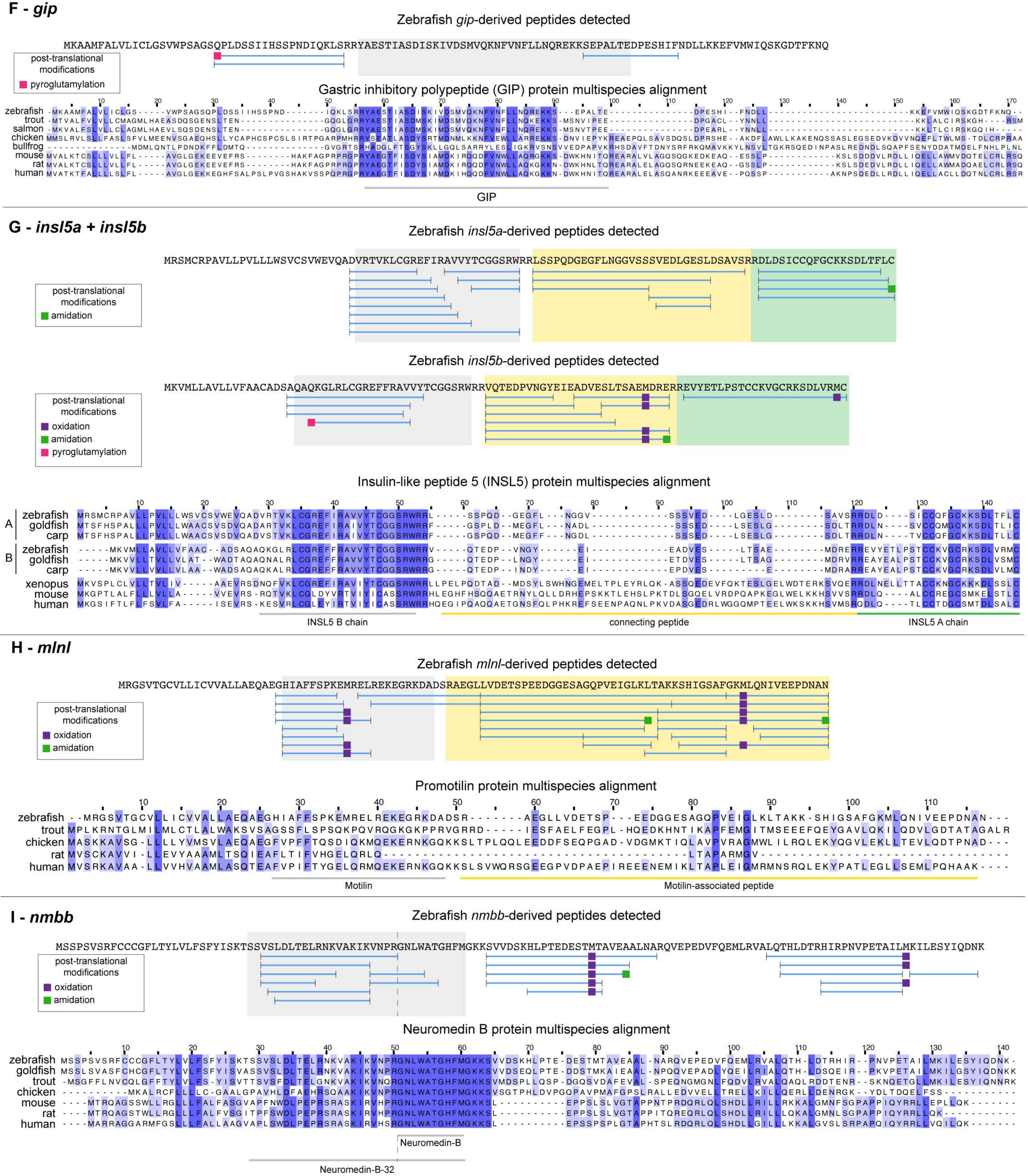
F - gip
Zebrafish gip-derived peptides detected
MKAAMFALVLICLGSVWPSAGSQPLDSSIIHSSPNDIQKLSRRYAESTIASDISKIVDSMVQKNFVNFLLNQREKKSEPALTEDPESHIFNDLLKKEFVMWIQSKGDTFKNQ
post-translational modifications
pyroglutamylation
Gastric inhibitory polypeptide (GIP) protein multispecies alignment
zebrafish
trout
salmon
chicken
bullfrog
mouse
rat
human
GIP
G - insl5a + insl5b
Zebrafish insl5a-derived peptides detected
MRSMCRPAVLLPVLLLWSVCSVWEVQADVRTVKLCGREFIRAVVYTCGGSRWRRLSSPQDGEGFLNGGVSSSVEDLGESLDSAVSRRDLDSICCQFGCKKSDLTFLC
post-translational modifications
amidation
Zebrafish insl5b-derived peptides detected
MKVMLLAVLLVFAACADSAQAQKGLRLCGREFFRAVVYTCGGSRWRRVQTEDPVNGYEIEADVESLTSAEMDRERREVYETLPSTCCKVGCRKSDLVRMC
post-translational modifications
oxidation
amidation
pyroglutamylation
Insulin-like peptide 5 (INSL5) protein multispecies alignment
A
zebrafish
goldfish
carp
B
zebrafish
goldfish
carp
xenopus
mouse
human
INSL5 B chain
connecting peptide
INSL5 A chain
H - mlnl
Zebrafish mlnl-derived peptides detected
MRGSVTGCVLLICVVALLAEQAEGHIAFFSPKEMRELREKEGRKDADSRAEGLLVDETSPEEDGGESAGQPVEIGLKLTAKKSHIGSAFGKMLQNIVEEPDNAN
post-translational modifications
oxidation
amidation
Promotilin protein multispecies alignment
zebrafish
trout
chicken
rat
human
Motilin
Motilin-associated peptide
I - nmbb
Zebrafish nmbb-derived peptides detected
MSSPSVSRFCCCGFLTYLVLFSFYISKTSSVSLDLTELRNKVAKIKVNPRGNLWATGHFMGKKSVVDSKHLPTEDESTMTAVEAALNARQVEPEDVFQEMLRVALQTHLDTRHIRPNVPETAILMKILESYIQDNK
post-translational modifications
oxidation
amidation
Neuromedin B protein multispecies alignment
zebrafish
goldfish
trout
chicken
mouse
rat
human
Neuromedin-B
Neuromedin-B-32

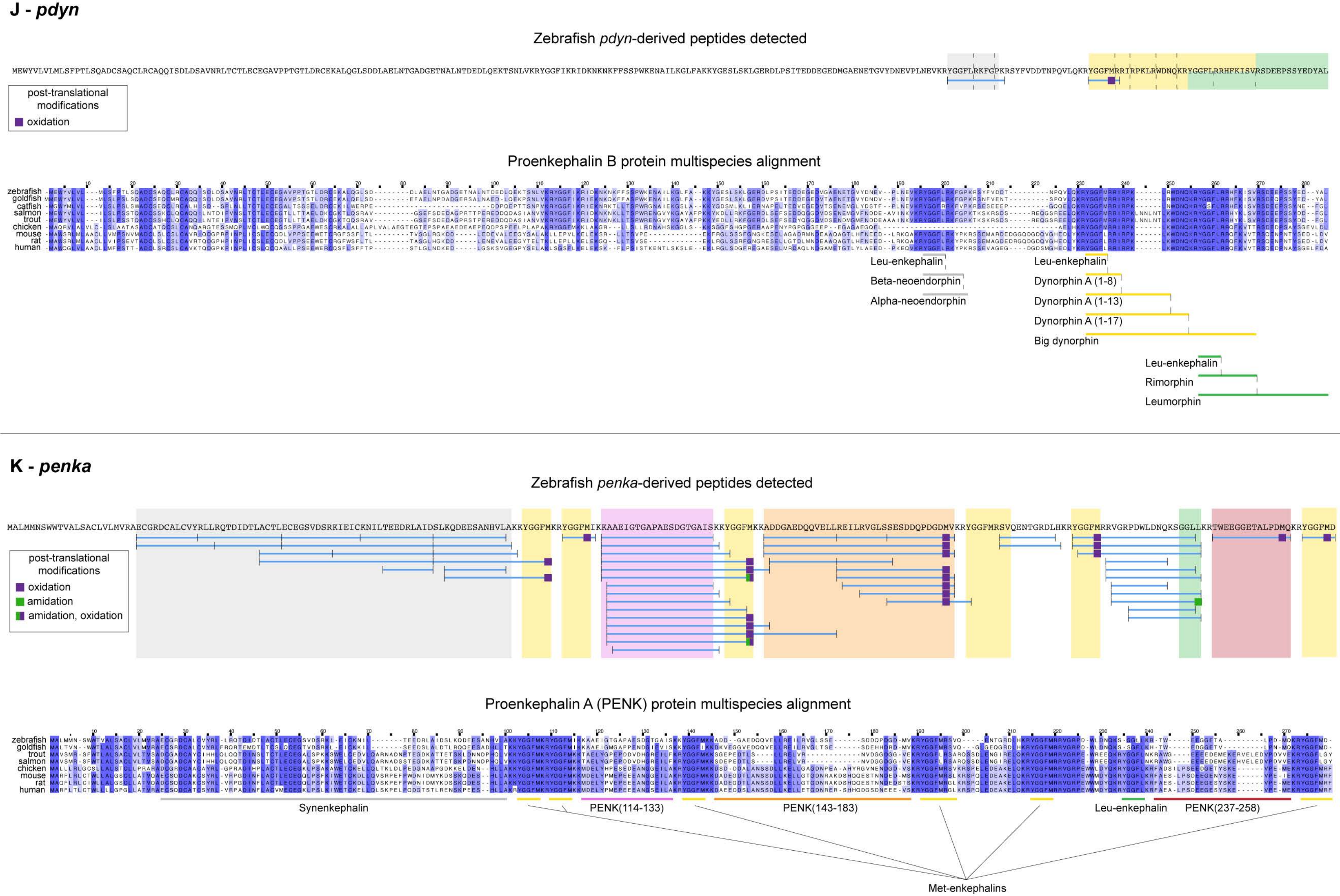

J - pdyn
Zebrafish pdyn-derived peptides detected
post-translational modifications
oxidation
Proenkephalin B protein multispecies alignment
zebrafish
goldfish
catfish
salmon
trout
chicken
mouse
rat
human
Leu-enkephalin
Beta-neoendorphin
Alpha-neoendorphin
Leu-enkephalin
Dynorphin A (1-8)
Dynorphin A (1-13)
Dynorphin A (1-17)
Big dynorphin
Leu-enkephalin
Rimorphin
Leumorphin
K - penka
Zebrafish penka-derived peptides detected
post-translational modifications
oxidation
amidation
amidation, oxidation
Proenkephalin A (PENK) protein multispecies alignment
zebrafish
goldfish
trout
salmon
chicken
mouse
rat
human
Synenkephalin
PENK(114-133)
PENK(143-183)
Leu-enkephalin
PENK(237-258)
Met-enkephalins

L - *pyyb*

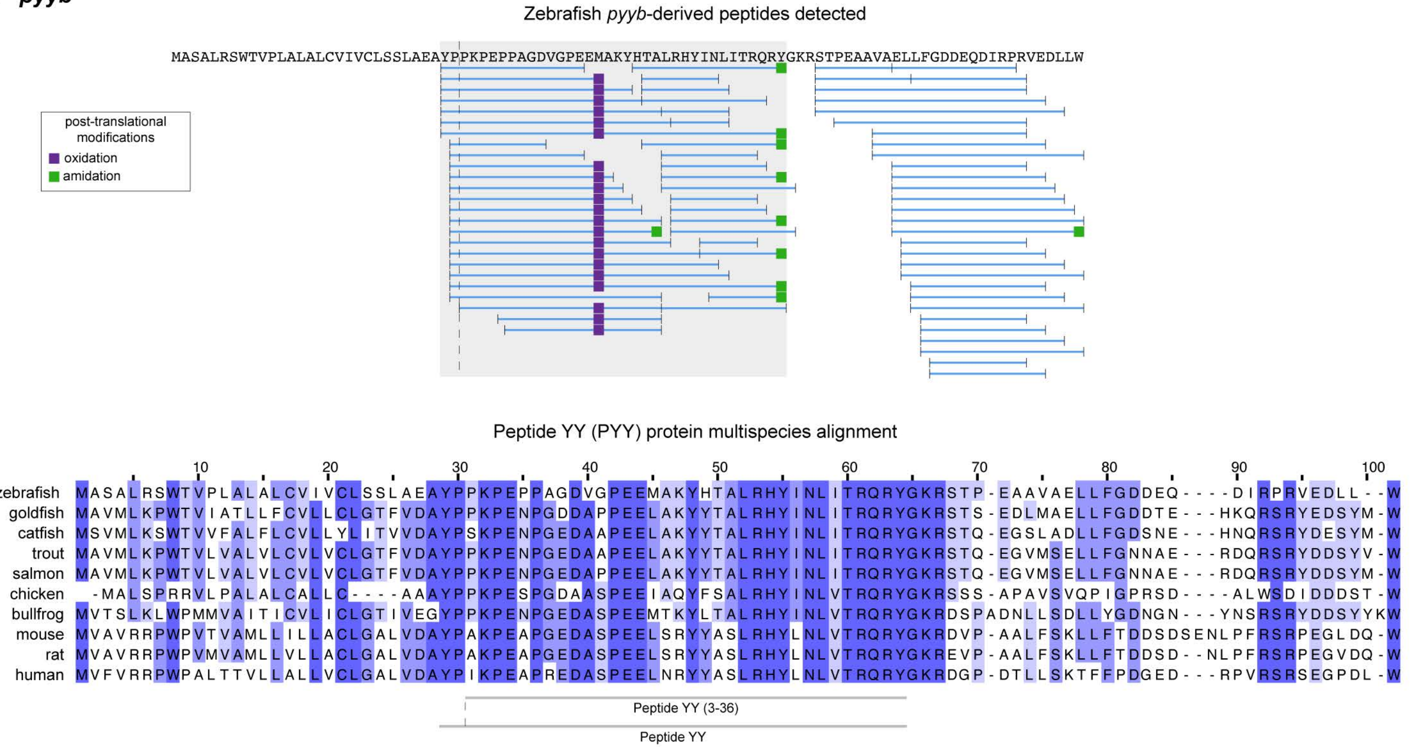

M - *sst2* + *sst1.2*

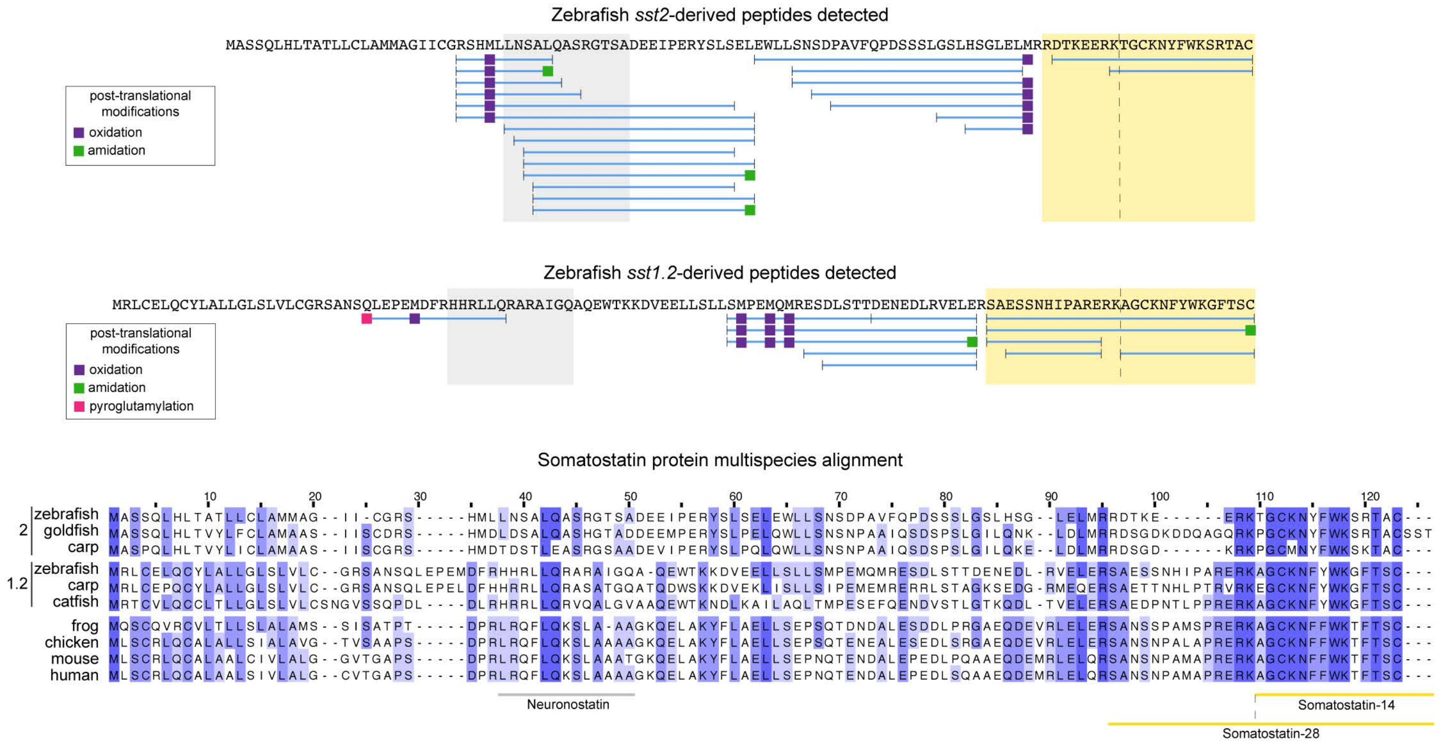

N - *tac3a*

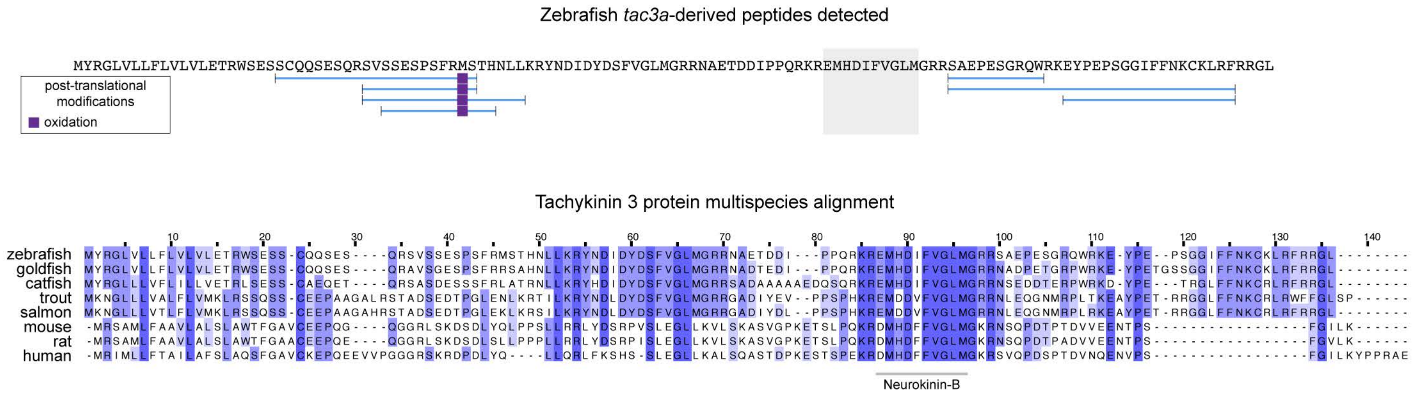

O - *vipb*

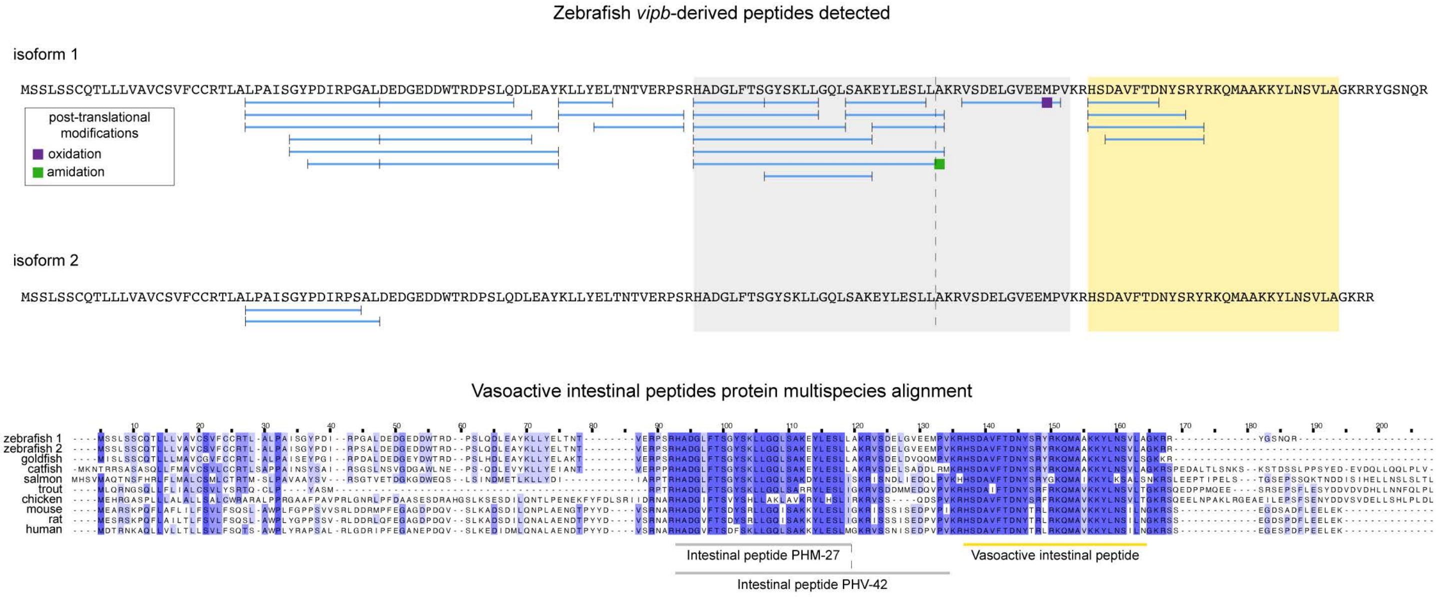

P - *ins*

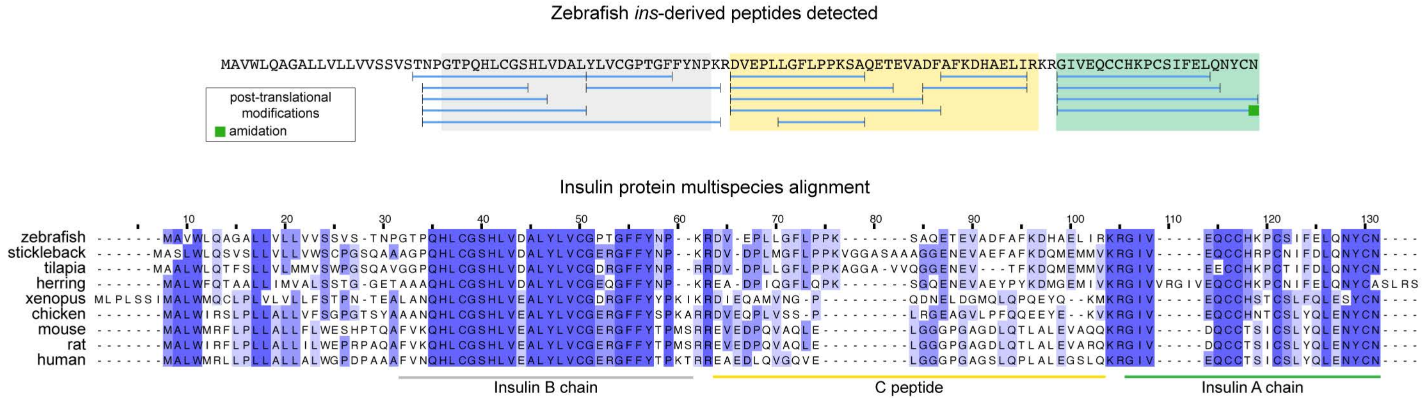

Q - *pcsk1nl*

Zebrafish *pcsk1nl*-derived peptides detected

MSGLLSSSLLFFLSLTLFQIHTPEAKPLSAMRGGVRSYDVPVRLRRELRDSVPYEAQMISYPSADFKSRSNDYYPSEVLRAQGLGQALQRLVESDQRREQEAAYLASMLRLLNEAQGTGQGRAGDQEEEGGDFQGPYPPDYDETEQA

VSMAKPQASWQGLLDPQLTQALLNRYKQERLVQAGLAPAANRIPEREQDKDQEMLRYLVEKILSSLASGGNQGSSSNPRAKRDLSAVSSIERPIKPALKRSRRSLDSAPGPQAEASLLRVKRLDDDDDVDEDAVAGQSNGTPHIGLQ

RMKRIDTDLPPPKKHSRKRRALSYDPALIAQHILQYLPA

R - *pcsk1*

Zebrafish *pcsk1*-derived peptides detected

MEERCRPMLLCSVLALVCALVSSAVDRQYLNEWAVEIPGGVQNARSIADEFGYQLVRQIGALENHYLFKRHSHPSRTKRSADHITKRLSEDDRVSWAEQQYEKRRAKRAPLGVECKDCSVDKLFDDPMWNQQWYLQDTRTSSSLPKL

DLHVIPVWKKGITGKGVVITVLDDGLEWNHTDIYPNYDPAASYDFNDNDPDPFPRYDSTNENKHGTRCAGEIAMQADNNKCGVGVAYNSKVGGIRMLDGIVTDAIEASSIGYNPDHVDIYSASWGPNDDGKTVEGPGRLAQKAFEYG

IQKGRGGKGSIFVWASGNGGRQGDNCDCDGYTDSLYTISISSASQQGLSPWYAEKCSSTLATAYSSGDYTDQRITSADLHNECTETHTGTSASAPLAAGIFALALEQNPDLTWRDLQHLVVWTSEFDPLANNPGWKRNGAGLMVNSR

FGFGLLNAKALVDLADPKVWKHVPEKKQCIVRDETFQPRPLKAAGEISIEIPTKACAGQANSVMSLEHVQVEVSIEYTRRGDLHITLTSPSGTTTVLLAERERDTSSNGFRNWAFMSVHTWGENPTGTWILKITDTSGRMENEGQII

SWKLILHGTSEKPEHMKKARVYTPYNAVQNDRRGVEPMEDMMKQEAPTPALKPEIPNQAIPDSPPSTASMSIPEVNSGPSTANLALLRLLQSAFNRQMPAAPPKSFQQERIPPQKLYQALDLLNRYRASQNGLFNDYSDNFYRAQPY

RHRDDRLLQALFDMLRDDQQ

S - *pcsk2*

post-translational modifications
■ oxidation

Zebrafish *pcsk2*-derived peptides detected

MRRFRGHHRTAPALFLTLIGALMMSAAAEDELFITGHFLVQMREAAPEDAQKLALEHGFESARKLPFGEDLFHFYPLEMPKTRRKRSLHQQHHRLASDHRVKNVFPQEGFGRQKRGYRDLPNTDVNMSDPLFTKQWYLINTGQADGT

PGLDLNVAEAWSLGFTGKGVTIAIMDDGIDYLHPDLASNYNAEASFDFSSNDPYPYPRYTDDWFNSHGTRCAGEVSAVSNNNICGVGVAYNSKVAGIRMLDQPFMTDIIEASSISHMPQVIDIYSASWGPTDDGKTVDGPRELTLQA

MADGVNKGRGGKGSIYVWASGDGGSYDDCNCDGYASSMWTISINSAINDGRTALYDESCSSTLASTFSNGRKRNPEAGVATTDLYGNCTLRHSGTSAAAPEAAGVFALALEANPNLTWRDLQHLTVLTSKRNKLHDEVHQWRRNGVG

LEFNHLFGYGVLDAGGMVKLARDWKTVPERFHCVAGSMQDIHKIQSGNKLLLSISTDACQGKDNFVRYLEHVQAVITVNASRRGDLNINMTSPMGTKSILLSRRPRDDDAKVGFDKWPFMTTHTWGEDPRGPWLLEVGFQSQSDMQS

GLLKEWTLMLHGTQSAPYIDQVVRDYQSKLAMSKKEELEEELDEAVERSLKSLLSKNN

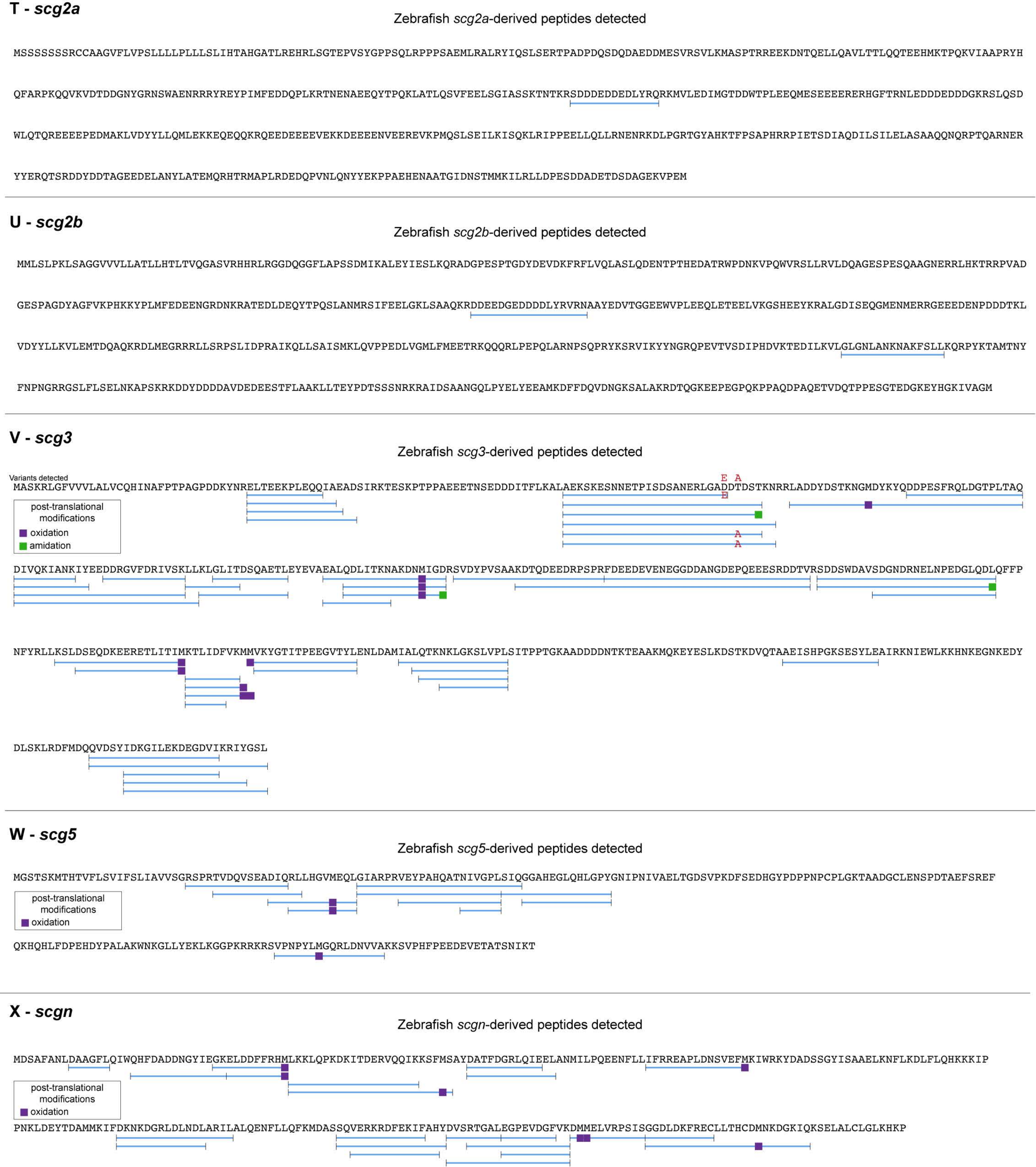

T - scg2a
Zebrafish scg2a-derived peptides detected
MSSSSSSSRCCAAGVFLVPSLLLLPLLLSLIHTAHGATLREHRLSGTEPVSYGPPSQLRPPPSAEMLRALRYIQSLSERTPADPDQSDQDAEDDMESVRSVLKMASPTRREEKDNTQELLQAVLTTLQQTEEHMKTPQKVIAAPRYH
QFARPKQQVKVDTDDGNYGRNSWAENRRRYREYPIMFEDDQPLKRTNENAEEQYTPQKLATLQSVFEELSGIASSKTNTKRSDDDEDDEDLYRQRKMVLEDIMGTDDWTPLEEQMESEEEERERHGFTRNLEDDDEDDDGKRSLQSD
WLQTQREEEEPEDMAKLVDYYLLQMLEKKEQEQQKRQEEDEEEEVEKKDEEEENVEEREVKPMQSLSEILKISQKLRIPPEELLQLLRNENRKDLPGRTGYAHKTFPSAPHRRPIETSDIAQDILSILELASAAQQNQRPTQARNER
YYERQTSRDDYDDTAGEEDELANYLATEMQRHTRMAPLRDEDQPVNLQNYYEKPPAEHENAATGIDNSTMMKILRLLDPESDDADETDSDAGEKVPEM
U - scg2b
Zebrafish scg2b-derived peptides detected
MMLSLPKLSAGGVVVLLATLLHTLTVQGASVRHHRLRGGDQGGFLAPSSDMIKALEYIESLKQRADGPESPTGDYDEVDKFRFLVQLASLQDENTPTHEDATRWPDNKVPQWVRSLLRVLDQAGESPESQAAGNERRLHKTRRPVAD
GESPAGDYAGFVKPHKKYPLMFEDEENGRDNKRATEDLDEQYTPQSLANMRSIFEELGKLSAAQKRDDEEDGEDDDDLYRVRNAAYEDVTGGEEWVPLEEQLETEELVKGSHEEYKRALGDISEQGMENMERRGEEEDENPDDDTKL
VDYYLLKVLEMTDQAQKRDLMEGRRRLLSRPSLIDPRAIKQLLSAISMKLQVPPEDLVGMLFMEETRKQQQRLPEPQLARNPSQPRYKSRVIKYYNGRQPEVTVSDIPHDVKTEDILKVLGLGNLANKNAKFSLLKQRPYKTAMTNY
FNPNGRRGSLFLSELNKAPSKRKDDYDDDDAVDEDEESTFLAAKLLTEYPDTSSSNRKRAIDSAANGQLPYELYEEAMKDFFDQVDNGKSALAKRDTQGKEEPEGPQKPPAQDPAQETVDQTPPESGTEDGKEYHGKIVAGM
V - scg3
Zebrafish scg3-derived peptides detected
Variants detected
E A
MASKRLGFVVVLALVCQHINAFPTPAGPDDKYNRELTEEKPLEQQIAEADSIRKTESKPTPPAEEETNSEDDDITFLKALAEKSKESNNETPISDSANERLGADDTDSTKNRRLADDYDSTKNGMDYKYQDDPESFRQLDGTPLTAQ
E
A
A
post-translational modifications
oxidation
amidation
DIVQKIANKIYEEDDRGVFDRIVSKLLKLGLITDSQAETLEYEVAEALQDLITKNAKDNMIGDRSVDYPVSAAKDTQDEEDRPSPRFDEEDEVENEGGDDANGDEPQEEESRDDTVRSDDSWDAVSDGNDRNELNPEDGLQDLQFFP
NFYRLLKSLDSEQDKEERETLITIMKTLIDFVKMMVKYGTITPEEGVTYLENLDAMIALQTKNKLGKSLVPLSITPPTGKAADDDDNTKTEAAKMQKEYESLKDSTKDVQTAAEISHPGKSESYLEAIRKNIEWLKKHNKEGNKEDY
DLSKLRDFMDQQVDSYIDKGILEKDEGDVIKRIYGSL
W - scg5
Zebrafish scg5-derived peptides detected
MGSTSKMTHTVFLSVIFSLIAVVSGRSPRTVDQVSEADIQRLLHGVMEQLGIARPRVEYPAHQATNIVGPLSIQGGAHEGLQHLGPYGNIPNIVAELTGDSVPKDFSEDHGYPDPPNPCPLGKTAADGCLENSPDTAEFSREF
post-translational modifications
oxidation
QKHQHLFDPEHDYPALAKWNKGLLYEKLKGGPKRRKRSVPNPYLMGQRLDNVVAKKSVPHFPEEDEVETATSNIKT
X - scgn
Zebrafish scgn-derived peptides detected
MDSAFANLDAAGFLQIWQHFDADDNGYIEGKELDDFFRHMLKKLQPKDKITDERVQQIKKSFMSAYDATFDGRLQIEELANMILPQEENFLLIFRREAPLDNSVEFMKIWRKYDADSSGYISAAELKNFLKDLFLQHKKKIP
post-translational modifications
oxidation
PNKLDEYTDAMMKIFDKNKDGRLDLNDLARILALQENFLLQFKMDASSQVERKRDFEKIFAHYDVSRTGALEGPEVDGFVKDMMELVRPSISGGDLDKFRECLLTHCDMNKDGKIQKSELALCLGLKHKP
